# Role of Gene Length in Control of Human Gene Expression: Chromosome-Specific and Tissue-Specific Effects

**DOI:** 10.1155/2021/8902428

**Published:** 2021-02-13

**Authors:** Jay C. Brown

**Affiliations:** Department of Microbiology, Immunology and Cancer Biology, University of Virginia School of Medicine, Charlottesville, Virginia 22908, USA

## Abstract

This study was carried out to pursue the observation that the level of gene expression is affected by gene length in the human genome. As transcription is a time-dependent process, it is expected that gene expression will be inversely related to gene length, and this is found to be the case. Here, I describe the results of studies performed to test whether the gene length/gene expression linkage is affected by two factors, the chromosome where the gene is located and the tissue where it is expressed. Studies were performed with a database of 3538 human genes that were divided into short, midlength, and long groups. Chromosome groups were then compared in the expression level of genes with the same length. A similar analysis was performed with 19 human tissues. Tissue-specific groups were compared in the expression level of genes with the same length. Both chromosome and tissue studies revealed new information about the role of gene length in control of gene expression. Chromosome studies led to the identification of two chromosome populations that differ in the expression level of short genes. A high level of expression was observed in chromosomes 2-10, 12-15, and 18 and a low level in 1, 11, 16-17, 19-20, 22, and 24. Studies with tissue-specific genes led to the identification of two tissues, brain and liver, which differ in the expression level of short genes. The results are interpreted to support the view that the level of a gene's expression can be affected by the chromosome and the tissue where the gene is transcribed.

## 1. Introduction

It is now well established that gene length is associated with the level of gene expression. A high level of expression is found in short genes while expression is weaker in longer ones [1–3]. The same association is observed in a wide variety of eukaryotic organisms [4–6], and there is a good reason to expect the linkage should exist. As time is required to complete the transcription of a pre-mRNA molecule, more short molecules are expected to be completed in the same time as fewer longer ones [7, 8]. The expected effect of transcription time is consistent with the experimentally observed higher expression of short genes.

One consequence of the association between gene length and gene expression is that length must exert a measure of control over the level of gene expression. If other factors are the same, then longer genes will be expressed at a lower level than shorter ones. If a long gene is to be expressed at a level higher than that determined by its length, then other mechanisms must be at work to adjust the level. Similarly, nonlength mechanisms need to be invoked if a short gene, determined by its length to be highly expressed, is found to be expressed at a low level. Expression of a gene may therefore be thought of as a background or default level determined by the gene's length overlaid by other mechanisms to adjust the level according to the requirements for the gene product. Such additional mechanisms may involve well-studied factors such as CpG islands, epigenetic signaling, promoters, and transcription factors [9, 10].

Here, I describe the results of a study designed to explore whether expression of a gene as determined by its length may be affected by (1) the chromosome on which the gene is located and (2) the tissue where it is expressed. Chromosome studies seek to determine, for example, whether genes with the same length may vary in their expression depending on the chromosome where the gene is located. Studies with tissues compare the expression of genes with the same length as they occur in different tissues.

The study was carried out with a database of human genes that includes both broadly expressed and tissue-targeted genes. These were divided into three groups, short, midlength, and long, and the presence of each gene group was compared among the 24 human chromosomes. The expression level of genes in chromosome subpopulations was then compared to identify chromosome-specific effects on gene expression. Short, midlength, and long gene populations were also compared for their expression in 19 human tissues. The results are interpreted to clarify the role of tissue-specific effects on the level of gene expression in each gene length group.

## 2. Materials and Methods

### 2.1. Gene Database

Studies were performed with a database of 3538 human genes. Of these, 2413 are tissue-targeted genes and 1125 are broadly expressed (i.e., housekeeping genes). Tissue-targeted genes were identified for the database from a GRO-seq analysis of genes expressed in IMR90 cells [11]. Nearly all the unexpressed genes in this cell line were found to have either selective or highly specific tissue expression, and these were accepted into the database. The database captures a substantial proportion of genes with tissue-targeted expression (i.e., tissue-selective and tissue specifically expressed genes). Estimates of the number of genes with tissue-targeted expression are in the range of 15% of the total number of human genes or ~3000 genes [9, 12], a value consistent with the view that the database (2413 genes) contains a substantial fraction of all tissue-targeted human genes. Broadly tissue-expressed genes in the database were derived from the HRT Atlas 1.0 (http://www.housekeeping.unicamp.br) [13]. Database genes are those judged to be broadly expressed in both the human and mouse genomes [13].

Five parameters were accumulated for each database tissue-targeted gene: (1) the chromosome, (2) the tissue where expression is the highest, (3) whether expression of the gene is tissue-selective or tissue-specific, (4) the level of expression, and (5) whether the gene length is short, midlength, or long. All information was downloaded from the UCSC Genome Browser human genome version hg38 (https://genome.ucsc.edu). Gene expression in a tissue was scored as “specific” if its expression is 10-fold or higher than expression in the tissue with the next highest expression level. Otherwise, the gene was scored as having “selective” expression. The lengths of short, midlength, and long genes were <15 kb, 15 kb–100 kb, and >100 kb, respectively. Parameters collected for broadly expressed genes were the same as those described above for targeted genes except that information about tissue expression was omitted. Only protein-coding and LINC RNA genes were included in the overall database. Pseudogenes and miRNA genes, for instance, were excluded. All gene database information is shown in Appendix A (Tables S1 and S2) and can be downloaded.

### 2.2. Data Handling

Data were manipulated with Word or Excel and rendered graphically with SigmaPlot v13.0.

## 3. Results

### 3.1. Gene Database: Tissue-Targeted and Broadly Expressed Genes

Analyses of gene expression were carried out separately with tissue-targeted and broadly expressed gene populations. As the two populations have quite different properties, it was judged that mixing them might obscure features that would be recognized in the separated groups [14, 15]. This applies especially to the effect of gene length which is reported to be longer in targeted than in broadly expressed gene populations [14, 15]. Handling the two populations separately avoids complications that might result from this feature.

**Figure 1:**
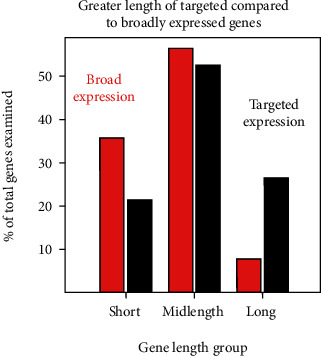
Proportion of short, midlength, and long genes in the databases of broadly expressed (red) and tissue-targeted genes (black). Note that the proportion of long genes is higher in the tissue-targeted database genes while the proportion of short genes is higher in broadly expressed genes.

For analysis, genes in the two expression populations were divided into three groups according to their length. Short, midlength, and long genes were >15 kb, 15 kb–100 kb, and >100 kb, respectively. In agreement with the prior analyses [14, 15], genes in the tissue-targeted population were found to be longer than the broadly expressed ones (Figure 1). Targeted genes were concentrated in the long and midlength groups while broadly expressed genes were concentrated in the short and midlength ranges (Figure 1).

### 3.2. Gene Database: Chromosome Dependence in the Targeted, Long Gene Population

**Figure 2 fig2:**
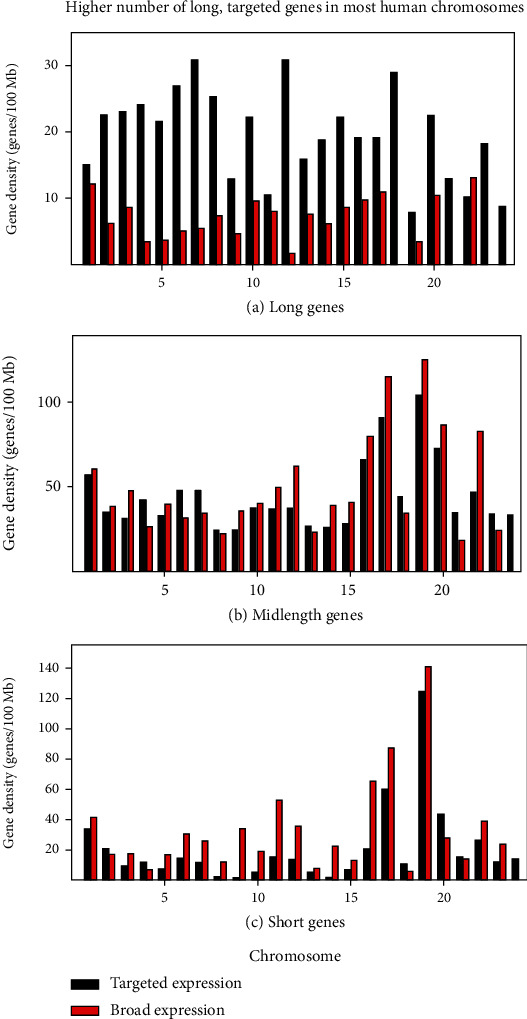
Distribution of short, midlength, and long genes among the 24 human chromosomes. Tissue-targeted genes are shown in black and broadly expressed in red. Note the higher proportion of long genes with tissue-targeted expression. Note also that the greater proportion of long, tissue-targeted genes is found in nearly all chromosomes (all but chromosome 18).

A study was carried out to determine whether the preponderance of long genes in the tissue-targeted compared to the broadly expressed population was found in all 24 human chromosomes or whether it might be limited to a chromosome subset. The study was performed because it was reasoned that the result might lead to the identification of chromosomes or chromosome subsets that favor hosting long genes. The proportion of long genes was therefore determined in each chromosome of the tissue-targeted population and compared to the same proportion in the broadly expressed genes (Figure 2(a)). As a control, the same analysis was performed with the midlength and short genes (Figures 2(b) and 2(c)).

The results showed that the proportion of long genes was higher in targeted compared to broadly expressed genes in nearly every chromosome: all but chromosome 22 (Figure 2(a)). The difference between targeted and broadly expressed genes could be striking; the difference was 3-fold or greater in 9 of the 24 chromosomes (Figure 2(a)). In contrast, midlength and short genes were more mixed in the proportion of targeted compared to broadly expressed short genes (Figures 2(b) and 2(c)). Among the midlength genes, for instance, 15 chromosomes had a higher proportion of broadly expressed genes compared to 9 targeted. For short genes, the ratio was 18 broadly to 6 targeted. The results support the view that the higher proportion of long genes with targeted compared to broad expression is found in most chromosomes and is not limited to a specific subset.

### 3.3. Gene Database: Inverse Relationship between Gene Length and Gene Expression

**Figure 3 fig3:**
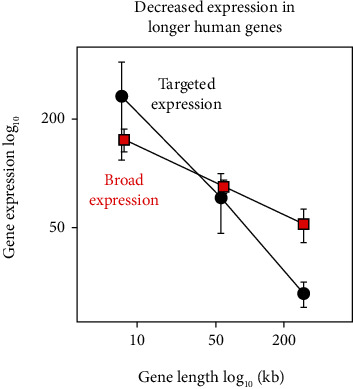
Expression of all database genes in the short, midlength, and long groups. Genes with tissue-targeted expression (RPKM) are shown in black, and those with broad expression (TPM) are shown in red. Note that gene expression is higher in short compared to long genes in both populations. Note also the greater range of expression values in tissue-targeted compared to broadly expressed genes.

It was expected that gene length would be inversely related to gene expression in the database genes, and that was found to be the case for both broadly expressed and tissue-targeted genes (Figure 3). The two gene populations were found to differ, however, in the range of expression values observed; the range was ~12-fold in the case of tissue-targeted genes compared to ~3-fold in the broadly expressed population (Figure 3). The greater range of expression in tissue-targeted genes is suggested to be consistent with the biology of the genes. It is reasonable to expect that the aggregate of functions in all distinct tissues will exceed the functions present in all or most tissues.

### 3.4. Identification of Chromosome-Specific Effects on Gene Expression: Overall Strategy

A two-step strategy was adopted for recognizing a chromosome-specific effect on gene expression. First, chromosomes were divided into subgroups based on clearly defined and unambiguous criteria. Second, pairs of such chromosome groups were examined for their expression level in genes in the same length class. A difference in expression level in the two chromosome groups would indicate that some feature of the chromosomes was influencing the extent of gene expression.

### 3.5. Identification of Chromosome-Specific Effects on Gene Expression: Chromosome Groups 1 and 2

**Figure 4:**
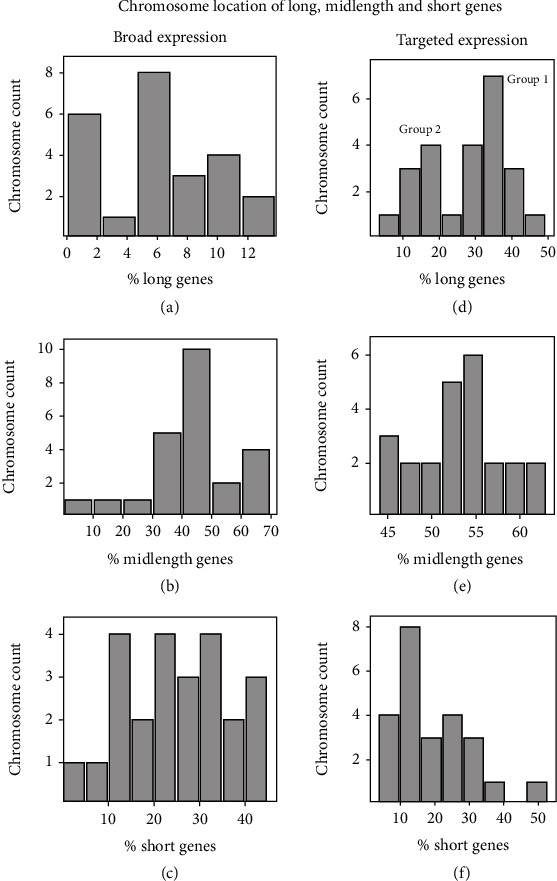
Chromosome count of short, midlength, and long genes as a function of their content in the 24 human chromosomes. Broadly expressed and tissue-targeted database genes are plotted separately. Note that distinct chromosome constellations are observed in all six chromosome populations. Group 1 and group 2 populations as shown in (d) were employed for studies of the effect of chromosome on gene expression (see Results).

Chromosome categories were defined according to their content of short, midlength, and long genes. For each chromosome, the values for the proportional content of short, midlength, and long genes were determined and the results were binned to create the chromosome groups used for analysis. The same operation was performed for the broadly expressed and tissue-targeted databases creating a total of 6 chromosome groups as shown in Figure 4. Chromosome bins were all considered appropriate for comparative analysis of gene expression.

Further examination was carried out with chromosome groups 1 and 2 as shown in Figure 4(d). The two groups differ in the proportion of long genes with group 1 the higher (Figure 4(d)). The two groups contained chromosomes 1, 11, 16-17, 19-20, and 22 (group 1) and chromosomes 2-10, 12-15, and 18 (group 2). Chromosome 13 was considered a border chromosome and was not analyzed.

Analysis of short gene transcription in the two chromosome groups demonstrated a higher level in group 1 (Table 1). The difference was 465.7 RPKM in 198 group 1 genes compared to 155.7 RPKM in 295 in group 2. Control experiments indicated that the results were selective for short gene expression (Table 1). With long genes, for instance, the difference was 23.0 RPKM (490 genes) in group 1 compared to 18.0 RPKM (112 genes) in group 2. In all genes, the results were 109.4 RPKM (1408 genes) in group 1 compared to 98.8 RPKM (887 genes) in group 2. The overall outcome supports the view that something about group 1 chromosomes potentiates the transcription of short genes in a way that is not observed in group 2 chromosomes.

A further issue is clarified by a control study carried out with all short, tissue-targeted genes (Table 1). The mean transcription in this gene population (267.9 RPKM; 518 genes) was found to be intermediate between the group 1 and group 2 levels and not the same as group 1 as expected (Table 1). The observation suggests expression of short genes in group 2 chromosomes may be actively suppressed and not simply transcribed at a default level found in the broader chromosome population.

### 3.6. Tissue Dependence of Gene Length

Tissue effects on gene expression were carried out only with the database of tissue-targeted genes as broadly expressed genes lack tissue targeting. Beginning with all genes in the tissue-targeted database, each gene was grouped according to its association with one of 19 human tissues. Each was also associated with one of the three length classes. The number of genes in each tissue group was then determined, and the counts are shown in Table 2.

It was striking to note that the highest number of short and midlength genes was found in three tissues, testis, brain, and spleen (Table 2). Testis and brain were also the top two in number of long genes. I interpret this result to indicate that testis, brain, and spleen may require the most genes based on the functions the tissues perform. Other tissues may express fewer genes simply because they do not need them. The high number of long genes in brain has been noted previously [16].

**Figure 5:**
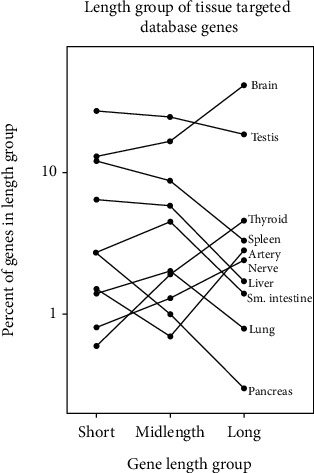
Distribution of database short, midlength, and long genes in ten human tissues. Values shown in plotted form here are selected from the longer list in Table 2. Note that tissues have distinct patterns of short, midlength, and long expressed genes.

Tissues were found in four groups based on their distribution of expressed short, midlength, and long genes. In seven of the 19 tissues examined, short genes were the most abundant and long genes the least (Table 2, group presented in italic). In four tissues, long genes were the most abundant (Table 2, group presented in bold). In the remaining two groups, (a) there was little difference among the short, midlength, and long genes or (b) midlength genes were either the highest or lowest in abundance (Table 2, groups presented in underline and bold italic, respectively). Results for selected tissues are shown graphically in Figure 5.

Reasonable interpretations suggest themselves for some of the results reported in Table 2. For instance, it is expected that tissues involved in synthesizing highly abundant extracellular products would make use of short, highly expressed genes. This is the result observed, for example, with testis, spleen, liver, skin, and pancreas. In contrast, brain depends on the function of long proteins involved in processes such as ion uptake, axon guidance, and cell adhesion, needs that would be served by expression of long, weakly expressed genes (Table 2).

### 3.7. Tissue-Specific Effects on Transcription Level

Tissue-specific effects of gene length on the transcription level were examined beginning with genes in the same length group. The transcription level of each gene was noted, and the results were compared among the panel of tissues. Controls were provided by the expression level of all database genes in the same length group.  Table 3 shows the results obtained with (1) long genes in four different tissues and (2) short genes in brain and liver. The results with long genes show three tissues where the transcription level resembles the control and one (muscle) where transcription is higher (Table 3). The outcome therefore identifies a tissue-specific effect of gene length in the case of muscle. The higher expression of muscle genes is suggested to be due to the high abundance in muscle of sarcomere tissue where all genes including long ones need to be expressed at a high level.

Studies with brain and liver identified tissue-specific effects in both cases. Expression of 33 short database genes in liver is found to be higher than in the control indicating a tissue-specific effect (480.5 RKPM (33 genes) compared to 267.9 RKPM (518 genes); Table 3). Similarly, expression was found to be lower in brain short genes also indicating a tissue-specific effect (Table 3). The higher expression in liver is suggested to result from the high level of proteins made for export from the liver. Synthesis of abundant exported proteins such as albumins and clotting factors is expected to require a higher level of gene expression than that needed for gene products used in the home cell only. The opposite situation is observed in brain. As most brain genes encode proteins used in the producing cell itself, overall gene expression can be low, even in short genes, expected on the basis of their length, to be expressed at a high level.

## 4. Discussion

### 4.1. Chromosome Gene Composition

The compositional differences among the human chromosomes shown here support the view that chromosomes differ significantly in character (Figures 1 [Fig fig2] [Fig fig3]–4). In chromosome 8, for instance, the density of tissue-targeted long genes is 25.3 genes/100 Mb of chromosome compared to 2.1 in the short genes. Such distinctions indicate the chromosomes have experienced quite different natural histories before and after they entered the genomes of human progenitor species. Much more now needs to be learned about chromosome evolution before the results in Figures 1 [Fig fig2] [Fig fig3]–4 can be reliably interpreted. For the present, it is safe to assert that the chromosomes are quite individual in their nature and that other aspects of their individuality are likely to emerge in the future [17, 18].

Among the most intriguing results of the chromosome composition studies has to do with the identification of two distinct populations of chromosomes, populations that differ in the proportion of long genes (Figure 4(d)). Fourteen chromosomes are found in one population and eight in the other. Together, the two populations account for 22 of the 24 human chromosomes suggesting the two groups may differ in a binary property that can be in only one state or the other.

The chromosome composition studies are also of interest because of the distribution identified among tissue-targeted, midlength genes (Figure 4(e)). In eleven of the 24 chromosomes, the proportion of midlength genes was found in a narrow distribution between ~52% and 56% of the total genes present. The narrow distribution suggests there is something about the structure or biochemistry of the eleven chromosomes that selects for midlength gene incorporation and retention in the genome. In contrast, the remaining 13 chromosomes do not demonstrate any similar selection related to midlength genes (Figure 4(e)). Instead, the proportion of midlength genes is found more evenly distributed over a wider range of midlength gene content. The result indicates that the selective force that creates a uniform proportion of midlength genes in eleven chromosomes is lacking in the others creating a wider range of allowed compositional levels.

### 4.2. Chromosome Effect on Gene Expression

The two chromosome populations (i.e., groups 1 and 2 in Figure 4(d)) are also of interest because they differ in the expression of short genes (Table 1). Short genes in the high-density, long chromosome population (group 1) are expressed at a higher level than the low-density population. It is tempting to suggest the population of high-density chromosomes just expresses all genes at a higher level, but this idea is ruled out by the control experiment. High expression is found only among the short and not the long genes (Table 1). The differences between the two chromosome populations may provide the basis for future studies to probe the biochemical properties that underlie their compositional and functional differences.

### 4.3. Tissue-Specific Effects

Tissue-specific effects of database genes were examined at two levels, (1) the proportion of short, midlength, and long genes present in a single tissue and (2) the expression level of genes in the same length class but in different tissues. Studies in the first group address the issue of whereas tissues differ quite significantly in the genes expressed, is this variability accompanied by variability in the distribution of gene lengths? Studies in the second group focus on gene expression. They ask whether genes in the same length group differ in expression when they are present in distinct tissues (Table 3).

Distinctive patterns of expressed gene length were observed in all 19 tissues examined (Table 2 and Figure 5). Although each tissue pattern was distinctive, four pattern groups could be recognized. They are as follows: (A) most genes are short with decreasing abundance of midlength and long genes; (B) most genes are long with decreasing abundance of midlength and short genes; (C) midlength genes are either the highest or lowest in abundance; and (D) short, midlength, and long genes are about equal in abundance. Table 2 shows tissues in the four patterns in italic, bold, bold italic, and underline, respectively.

One way to interpret the above pattern groups is to focus on whether a tissue is involved in synthesizing and exporting a protein product. Such export is found in group A tissues including testis, liver, and pancreas. These tissues are involved in export of sperm, plasma proteins, and digestive enzymes, respectively. As export of such products is expected to require a higher level of gene expression than expression for host cell use only, it is reasonable that short, highly expressed genes should be used. Group B tissues, on the other hand, do not synthesize a high number of proteins for export. These tissues such as brain and thyroid produce protein products for local purposes such as neuron function (brain) and small molecule synthesis (thyroid). It is understandable that such tissues should be enriched in long, weakly expressed genes as reported here (Table 2 and Figure 5).

It was expected that genes of the same length would be found to have quite different expression levels depending on the tissue examined and this was found to be the case (Table 3). It was considered important, however, to establish this issue with genes and tissues from the same database. Expression of long genes in four tissues establishes the point. Mean long gene expression in all four tissues differs from each other and from the mean of all long database genes. A similar result was obtained with short genes from liver and brain. Together, the results support the view that tissue-specific effects influence expression of genes in the same length group.

### 4.4. Sensing Gene Length

The results shown here addressing effects of gene length indicate that there need to be cellular mechanisms able to sense gene length. It would be impossible, for instance, for chromosome 19 to have a high density of short genes (123.8 genes/100 Mb of chromosome; Figure 2(c)) if there was no mechanism for the cell to sense short genes or something that correlates with gene shortness. It is relevant therefore that mechanisms of the expected type have been reported in the case of long genes. Studies with mouse, for instance, have demonstrated that the Mecp2 gene selectively downregulates long gene expression [19]. Mecp2 is found to act by binding selectively to methylated CA-containing DNA sites in long genes. Depletion of the same activity in the human homolog, MECP2, is found to cause Rett syndrome [20].

Two other instances have been reported [21, 22]. The protein encoded by the human SFPQ gene is found to bind to introns in long genes ensuring that transcription will proceed to the end of the gene. Similarly, human topoisomerases TOPI and TOPII have been shown to facilitate the transcription of long genes in neurons [23]. In view of the results reported here, it is reasonable to suggest that there may be similar cellular systems to recognize short and midlength genes.

## Figures and Tables

**Table 1:** Gene expression in group 1 compared to group 2 chromosomes.

Chromosome pool	Long genes	Short genes	All genes
Group 1: high long gene content^a^			
Mean transcription^c^	23.0	465.7	109.4
Std error	2.1	188.2	28.0
No. of genes	490	198	1408
Group 2: low long gene content^b^			
Mean transcription	18.0	155.7	98.8
Std error	3.3	40.2	19.5
No. of genes	112	295	887
All chromosomes			
Mean transcription	21.5	267.9	101.4
Std error	1.7	75.7	17.9
No. of genes	636	518	2413

^a^Group 1: chromosomes 2-10, 12-15, and 18. ^b^Group 2: chromosomes 1, 11, 16-17, 19-20, 22, and 24. ^c^RPKM.

**Table 2:** Tissue specificity of genes in the tissue-targeted database.

	Short genes		Midlength genes		Long genes		All genes	
Tissue	All^a^	%	All	%	All	%	All	%
*Testis*	141	27.2	311	24.7	119	18.7	571	23.7
**Brain**	67	12.9	207	16.5	262	41.2	536	22.2
*Spleen*	62	12.0	112	8.9	21	3.3	195	8.1
*Liver*	33	6.4	73	5.8	11	1.7	117	4.9
*Skin*	27	5.2	61	4.9	9	1.4	97	4.0
***Sm. intestine***	14	2.7	57	4.5	9	1.4	80	3.3
***Esophagus***	14	2.7	46	3.7	11	1.7	71	2.9
***Kidney***	10	1.9	46	3.7	10	1.6	66	2.7
Muscle	12	2.3	33	2.6	16	2.5	61	2.5
Pituitary	15	2.9	31	2.5	16	2.5	62	2.6
*Adipose*	11	2.1	26	2.1	8	1.3	45	1.9
**Heart**	7	1.4	25	2.0	16	2.5	48	2.0
Lung	7	1.4	25	2.0	5	0.8	37	1.5
**Thyroid**	3	0.6	24	1.9	29	4.6	56	2.3
Adrenal	4	0.8	16	1.3	7	1.1	27	1.1
**Nerve**	4	0.8	16	1.3	15	2.4	35	1.5
*Pancreas*	14	2.7	12	1.0	2	0.3	28	1.2
***Artery***	8	1.5	9	0.7	18	2.8	35	1.5
*Stomach*	8	1.5	11	0.9	2	0.3	21	0.9
Other	57	11.0	116	9.2	50	7.9	223	9.2
Total	518	100.0	1257	100.0	636	100.0	2411	100.0

^a^All 24 chromosomes.

**Table 3:** Effect of tissue on expression of genes in the same length group.

Tissue	Gene length group	Mean transcription (RPKM)	Standard error	Number of genes
Spleen	Long	28.2	12.7	21
Muscle	Long	82.5	29.6	16
Pituitary	Long	16.2	5.2	16
Thyroid	Long	39.0	17.4	29
All tissues	Long	21.5	1.7	636
Brain	Short	47.8	11.8	66
Liver	Short	480.5	158.2	33
All tissues	Short	267.9	75.7	518

## Data Availability

All data employed in this study are contained in Tables S1 and S2 of the Supplementary Material from which it can be freely downloaded.

## Supplementary Materials

Supplementary 1 All supplementary data is contained in Tables S1 and S2. Table S1: list of all 2413 genes with tissue-targeted expression used for the analysis described here together with the following information about each gene: gene name, chromosome, tissue of primary expression, whether the gene is expressed in one tissue only (tissue-specific expression) or in several tissues (tissue-selective expression), gene expression level, and gene length group (short, midlength, or long).

Supplementary 2 Table S2: list of all 1125 broadly expressed genes together with the same information described above except for information about tissue expression.
