# Supplementary material for: Role of Gene Length in Control of Human Gene Expression: Chromosome-Specific and Tissue-Specific Effects
Source: Int J Genomics. 2021 Feb 13;2021:8902428. doi: 10.1155/2021/8902428 (PMC7911607; doi:10.1155/2021/8902428)
Supplement: Supplementary 1 — All supplementary data is contained in Tables S1 and S2. Table S1: list of all 2413 genes with tissue-targeted expression used for the analysis described here together with the following information about each gene: gene name, chromosome, tissue of primary expression, whether the gene is expressed in one tissue only (tissue-specific expression) or in several tissues (tissue-selective expression), gene expression level, and gene length group (short, midlength, or long). [file 8902428.f1.docx]

**Supplementary Data**. Table S1: All database genes with tissue targeted expression

TSE/TSP: Tissue SElective Expression/Tissue SPecific Expression. All database genes are biased in their tissue expression. TSP genes are expressed in only a single tissue while TSE genes are expressed in some but not all tissues listed by UCSC Browser.

Tissue: For TSE genes, the tissue listed is the one with the highest expression of those listed by UCSC. sm intestine: small intestine.

Exp: Gene Expression Level. Units are RPKM. Data were downloaded from the UCSC Genome Browser ( <http://genome.ucsc.edu> ).

Gene Length: Short, Mid Length and Long Genes are <15kb, 15kb-100kb and >100kb, respectively. Genes present in both chromosomes 23 and 24 are counted only in chromosome 23.

Chr1: All database tissue targeted genes on chromosome 1

| Index | Gene | Tissue | TSE/TSP | Exp | Length |
| --- | --- | --- | --- | --- | --- |
| 1 | RNF223 | esophagus | E | 14.8 | Short |
| 2 | TTLL10 | testis | P | 31.2 | Short |
| 3 | GABRD | brain | P | 269.2 | Short |
| 4 | MMEL1 | testis | P | 15.8 | Mid |
| 5 | TTC34 | testis | P | 3.2 | Long |
| 6 | ACTRT2 | testis | P | 174.2 | Short |
| 7 | CA6 | skin | P | 7.6 | Mid |
| 8 | LRRC38 | adrenal | E | 10.2 | Mid |
| 9 | FHAD1 | testis | P | 10.9 | Long |
| 10 | C1orf64 | brain | E | 2.4 | Short |
| 11 | PADI2 | muscle | E | 73.5 | Mid |
| 12 | PADI3 | bladder | E | 23.1 | Mid |
| 13 | PADI6 | bone marrow | E | 0.1 | Mid |
| 14 | ACTL8 | testis | P | 0.1 | Mid |
| 15 | PAX7 | muscle | P | 0.7 | Long |
| 16 | PLA2G2A | intestine | E | 412.8 | Short |
| 17 | PLA2G5 | heart | E | 45.7 | Mid |
| 18 | PLA2G2F | bladder | E | 13.1 | Short |
| 19 | UBXN10 | colon | E | 32.3 | Short |
| 20 | FAM43B | adrenal | E | 15.2 | Short |
| 21 | ALPL | lung | E | 49.8 | Mid |
| 22 | CELA2B | pancreas | P | 1080.4 | Mid |
| 23 | EPHA8 | spleen | E | 2.1 | Mid |
| 24 | LACTBL1 | testis | P | 0.3 | Short |
| 25 | CNR2 | spleen | P | 7.0 | Mid |
| 26 | MYOM3 | muscle | E | 23.2 | Mid |
| 27 | IL22RA1 | pancreas | E | 86.2 | Mid |
| 28 | NCMAP | nerve | P | 49.4 | Mid |
| 29 | RUNX3 | spleen | E | 24.7 | Mid |
| 30 | RHCE | pituitary | E | 1.8 | Mid |
| 31 | TRIM63 | muscle | P | 402.3 | Mid |
| 33 | ZNF683 | testis | P | 59.0 | Short |
| 34 | LIN28A | testis | P | 5.5 | Mid |
| 35 | NR0B2 | liver | E | 40.9 | Short |
| 36 | KDF1 | skin | E | 12.5 | Short |
| 37 | FCN3 | lung | E | 456.1 | Short |
| 38 | CD164L2 | skin | E | 3.1 | Short |
| 41 | OPRD1 | brain | E | 0.3 | Mid |
| 44 | LCK | lymph node | E | 46.5 | Mid |
| 45 | ZBTB8B | brain | E | 0.3 | Mid |
| 46 | CSMD2 | brain | E | 0.7 | Long |
| 47 | C1orf94 | testis | P | 19.5 | Mid |
| 48 | GJB5 | skin | E | 63.3 | Short |
| 49 | GJB4 | skin | P | 25.3 | Short |
| 50 | DLGAP3 | brain | E | 22.8 | Mid |
| 51 | CSF3R | spleen | E | 178.4 | Mid |
| 52 | GRIK3 | nerve | E | 10.9 | Long |
| 53 | RSPO1 | uterus | E | 23.7 | Mid |
| 54 | EPHA10 | testis | E | 14.8 | Mid |
| 55 | POU3F1 | nerve | E | 18.2 | Short |
| 56 | BMP8A | thyroid | E | 7.1 | Mid |
| 58 | NT5C1A | muscle | E | 4.8 | Short |
| 59 | HPCAL4 | brain | E | 77.9 | Short |
| 60 | COL9A2 | pituitary | E | 100.5 | Mid |
| 61 | RIMS3 | brain | E | 79.4 | Mid |
| 62 | KCNQ4 | esophagus | E | 10.9 | Mid |
| 63 | GUCA2B | sm intestine | E | 56.6 | Short |
| 64 | GUCA2A | sm intestine | E | 523.7 | Short |
| 65 | RIMKLA | brain | E | 6.3 | Mid |
| 66 | ZMYND12 | testis | E | 25.4 | Mid |
| 67 | CCDC30 | testis | E | 3.1 | Long |
| 68 | CLDN19 | nerve | E | 69.8 | Short |
| 69 | FAM183A | pituitary | E | 10.2 | Short |
| 70 | TMEM125 | lung | E | 37.3 | Short |
| 71 | C1orf210 | colon | E | 21.5 | Short |
| 73 | KLF17 | testis | P | 10.9 | Mid |
| 74 | BEST4 | colon | E | 25.7 | Short |
| 75 | PTCH2 | testis | E | 20.4 | Mid |
| 76 | ZSWIM5 | adrenal | E | 10.3 | Long |
| 78 | DMBX1 | testis | E | 1.2 | Short |
| 79 | CYP4B1 | nerve | E | 67.5 | Mid |
| 81 | CYP4A11 others | liver | E | 155.0 | Short |
| 83 | TRABD2B | artery | E | 29.6 | Long |
| 85 | AGBL4 | brain | E | 1.0 | Long |
| 86 | ELAVL4 | brain | E | 16.5 | Mid |
| 87 | DMRTA2 | testis | E | 2.1 | Short |
| 88 | FAM159A | spleen | E | 2.1 | Mid |
| 89 | DMRTB1 | testis | P | 58.7 | Short |
| 90 | LDLRAD1 | salivary gland | E | 1.0 | Short |
| 91 | TTC22 | esophagus | E | 51.1 | Mid |
| 92 | LEXM | esophagus | E | 6.9 | Mid |
| 93 | BSND | kidney | P | 6.2 | Mid |
| 94 | PCSK9 | liver | E | 14.1 | Mid |
| 95 | C1orf168 | liver | E | 7.3 | Long |
| 96 | C8A | liver | P | 175.8 | Mid |
| 97 | C8B | liver | P | 179.8 | Mid |
| 98 | HOOK1 | testis | E | 25.5 | Mid |
| 99 | CYP2J1 | heart | E | 25.9 | Mid |
| 100 | C1orf87 | testis | P | 3.2 | Mid |
| 101 | KANK4 | nerve | E | 12.1 | Mid |
| 102 | LINC00466 | testis | P | 2.0 | Long |
| 103 | UBE2U | testis | P | 18.6 | Mid |
| 104 | INSL5 | testis | E | 0.4 | Short |
| 105 | C1orf141 | testis | P | 5.6 | Long |
| 106 | IL23R | testis | E | 0.6 | Mid |
| 107 | RPE65 | brain | P | 2.3 | Mid |
| 108 | LRRC7 | brain | E | 2.2 | Long |
| 109 | ERICH3 | testis | E | 14.4 | Long |
| 110 | LHX8 | salivary gland | E | 1.3 | Mid |
| 111 | SLC44A5 | testis | E | 2.9 | Long |
| 112 | ASB17 | testis | P | 37.5 | Short |
| 113 | GIPC2 | adrenal | E | 14.4 | Mid |
| 114 | IFI44L | brain | E | 5.0 | Mid |
| 115 | MCOLN3 | adrenal | E | 21.7 | Mid |
| 116 | CLCA2 | esophagus | E | 71.6 | Mid |
| 117 | CLCA1 | sm intestine | E | 213.2 | Mid |
| 119 | GBP7 | liver | P | 16.5 | Mid |
| 121 | GBP5 others | spleen | E | 9.2 | Short |
| 122 | BARHL2 | brain | E | 34.8 | Short |
| 123 | BRDT | testis | P | 52.0 | Mid |
| 124 | LPPR5 | brain | E | 4.2 | Long |
| 125 | OLFM3 | brain | P | 9.5 | Long |
| 126 | AMY2A | pancreas | P | 4299.3 | Short |
| 127 | AMY1A others | pancreas | P | 1.3 | Short |
| 128 | NBPF4 | testis | E | 1.5 | Mid |
| 129 | NBPF6 | testis | E | 1.4 | Mid |
| 130 | SPATA42 | testis | P | 154.0 | Short |
| 131 | SYPL2 | muscle | E | 70.0 | Mid |
| 132 | GPR61 | pituitary | E | 4.2 | Short |
| 133 | GSTM1 | ovary | E | 33.9 | Short |
| 134 | EPS8L3 | sm intestine | E | 89.3 | Short |
| 135 | PROK1 | ovary | E | 161.0 | Short |
| 136 | KCNA2 | brain | E | 7.6 | Short |
| 137 | CHI3L2 | salivary gland | E | 34.6 | Mid |
| 141 | SYCP1 | testis | P | 21.3 | Long |
| 142 | CASQ2 | heart | E | 331.1 | Mid |
| 143 | MAB21L3 | esophagus | E | 3.3 | Mid |
| 144 | IGSF3 | skin | E | 12.7 | Mid |
| 145 | C1orf137 | lung | E | 0.4 | Short |
| 146 | CD2 | spleen | E | 25.3 | Short |
| 148 | CD101 | spleen | E | 1.7 | Mid |
| 149 | VTCN1 | salivary gland | E | 12.1 | Mid |
| 150 | SPAG17 | testis | E | 12.0 | Long |
| 151 | TBX15 | muscle | E | 36.2 | Long |
| 152 | HAO2 | liver | E | 82.7 | Mid |
| 153 | HSD3B2 | adrenal | E | 884.8 | Short |
| 154 | HSD3B1 others | sm intestine | P | 6.1 | Short |
| 155 | REG4 | sm intestine | E | 100.5 | Mid |
| 159 | HFE2 | muscle | E | 105.1 | Short |
| 160 | ANKRD35 | skin | E | 45.5 | Mid |
| 163 | NBPF11 | skin | E | 15.7 | Mid |
| 164 | GJA5 | placenta | E | 23.7 | Mid |
| 168 | RIAD1 | pituitary | E | 1.2 | Short |
| 169 | RORC | muscle | E | 13.1 | Mid |
| 170 | THHL1 | skin | P | 0.1 | Short |
| 171 | TCHH | spleen | E | 1.7 | Short |
| 172 | RPTN | skin | P | 5.4 | Short |
| 173 | CRNN | esophagus | E | 3027.7 | Short |
| 174 | LCE5A | skin | P | 47.5 | Short |
| 175 | LCE3A others | testis | ? | 0.3 | Short |
| 176 | SMCP | testis | P | 743.8 | Short |
| 177 | SPRR4 other | skin | E | 17.7 | Short |
| 178 | PGLYRP4 | skin | E | 9.1 | Mid |
| 179 | S100A7 | skin | E | 29.5 | Short |
| 180 | NPR1 | artery | E | 95.4 | Mid |
| 181 | TDRD10 | testis | E | 62.0 | Mid |
| 182 | KCNN3 | brain | E | 5.0 | Long |
| 184 | C1orf61 | brain | P | 54.1 | Short |
| 185 | HAPLN2 | brain | E | 275.9 | Short |
| 186 | NTRK1 | prostate | E | 2.4 | Mid |
| 187 | ETV3L | brain | P | 0.4 | Mid |
| 188 | FCRL5 | spleen | E | 13.1 | Mid |
| 189 | FCRL4 others | spleen | E | 0.5 | Mid |
| 190 | CD5L | spleen | E | 264.4 | Short |
| 191 | CD1D others | spleen | E | 22.9 | Short |
| 192 | SPTA1 | testis | E | 0.9 | Mid |
| 193 | MNDA | spleen | E | 51.4 | Mid |
| 194 | PYHIN1 | spleen | E | 4.8 | Mid |
| 195 | AIM2 | spleen | E | 16.1 | Short |
| 196 | CADM3 | brain | E | 218.9 | Mid |
| 197 | FCER1A | skin | E | 6.7 | Mid |
| 198 | KCNJ9 | brain | P | 60.8 | Short |
| 199 | ATP1A4 | testis | P | 13.3 | Mid |
| 200 | SLAMF6 | spleen | E | 28.5 | Mid |
| 201 | CD84 | spleen | E | 8.8 | Mid |
| 202 | SLAMF1 | spleen | E | 3.7 | Mid |
| 203 | CD48 | spleen | E | 43.9 | Mid |
| 204 | SLAMF7 | spleen | E | 22.4 | Mid |
| 205 | LY9 | spleen | E | 8.1 | Mid |
| 206 | CD244 | spleen | E | 9.1 | Mid |
| 207 | ITLN1 | adipose | E | 141.3 | Short |
| 208 | PCP4L1 | brain | E | 119.8 | Mid |
| 209 | FCGR2C | adipose | E | 3.9 | Mid |
| 210 | FCGR2B | spleen | E | 14.4 | Mid |
| 211 | FCRLA | spleen | E | 18.9 | Short |
| 212 | C1orf226 | pituitary | E | 2.0 | Short |
| 213 | SH2D1B | spleen | E | 3.6 | Mid |
| 215 | LMX1A | pituitary | E | 1.4 | Long |
| 216 | RXRG | pituitary | E | 21.7 | Mid |
| 217 | LRRC52 | testis | P | 17.6 | Mid |
| 219 | ILDR2 | testis | E | 8.4 | Mid |
| 220 | MAEL | testis | P | 86.4 | Mid |
| 221 | GPA33 | colon | E | 107.9 | Mid |
| 222 | DUSP27 | muscle | E | 37.3 | Mid |
| 223 | CD247 | spleen | E | 14.7 | Mid |
| 224 | RCSD1 | spleen | E | 24.0 | Mid |
| 225 | XCL2 | spleen | E | 5.9 | Short |
| 226 | XCL1 | spleen | E | 2.1 | Short |
| 227 | DPT | colon | E | 248.8 | Mid |
| 228 | F5 | liver | E | 21.5 | Mid |
| 229 | SELP | lung | E | 8.0 | Mid |
| 230 | SELL | spleen | E | 86.7 | Mid |
| 231 | SELE | fallopian tube | E | 16.5 | Short |
| 232 | METTL11B | heart | P | 0.5 | Mid |
| 233 | LINC01142 | testis | P | 1.2 | Short |
| 234 | MROH9 | testis | P | 1.5 | Mid |
| 235 | FMO1 | kidney | E | 9.2 | Mid |
| 236 | MYOC | esophagus | E | 113.1 | Mid |
| 237 | FASLG | spleen | E | 2.6 | Short |
| 238 | SLC9C2 | testis | P | 4.4 | Long |
| 239 | TNN | heart | E | 3.0 | Mid |
| 241 | TNR | brain | P | 14.4 | Long |
| 242 | ASTN1 | brain | E | 16.3 | Long |
| 243 | BRINP2 | brain | E | 21.3 | Long |
| 244 | TDRD5 | testis | E | 12.4 | Mid |
| 245 | FAM163A | pituitary | E | 24.5 | Mid |
| 246 | CACNA1E | brain | P | 3.3 | Long |
| 247 | RGS8 | brain | E | 2.6 | Mid |
| 248 | RGS21 others | brain | ? low | 0.1 | Mid |
| 249 | CFHR1 others | liver | E | 228.2 | Short |
| 250 | LINC01222 | appendix | Low | 0.1 | Short |
| 251 | IGFN1 | muscle | E | 34.8 | Mid |
| 252 | TNNT2 | heart | E | 2058.9 | Mid |
| 253 | TNNT1 | muscle | E | 2311.1 | Mid |
| 255 | CHIT1 | spleen | E | 1.1 | Short |
| 256 | LRRN2 | brain | E | 6.5 | Mid |
| 257 | NFASC | brain | E | 16.7 | Long |
| 258 | CTSE | stomach | E | 144.8 | Short |
| 259 | IL10 | spleen | E | 3.6 | Short |
| 260 | PIGR | colon | E | 999.6 | Mid |
| 261 | FCAMR | kidney | E | 3.6 | Short |
| 262 | C1orf116 | esophagus | E | 53.8 | Short |
| 264 | C4BPA | liver | E | 262.8 | Mid |
| 265 | CR2 | spleen | E | 40.2 | Mid |
| 266 | CR1 | spleen | E | 15.0 | Long |
| 267 | RD3 | bladder | E | 0.6 | Mid |
| 268 | PROX1 | liver | E | 18.8 | Mid |
| 269 | LINC00210 | testis | low | 0.1 | Mid |
| 270 | SLC30A10 | liver | E | 7.6 | Short |
| 271 | MARK1 | brain | E | 7.1 | Long |
| 273 | TLR5 | ovary | E | 8.2 | Mid |
| 274 | CAPN8 | stomach | E | 21.5 | Long |
| 275 | LEFTY2 | ovary | E | 17.4 | Short |
| 276 | STUM | brain | E | 28.5 | Mid |
| 277 | PRSS38 | testis | P | 4.2 | Mid |
| 279 | PGBD5 | brain | E | 29.8 | long |
| 280 | AGT | liver | E | 630.3 | Short |
| 281 | TRIM67 | brain | P | 6.9 | Mid |
| 282 | MAP3K21 | pancreas | E | 5.1 | Mid |
| 283 | GNG4 | brain | E | 20.8 | Long |
| 284 | EDARADD | bladder | E | 2.7 | Mid |
| 285 | ACTN2 | muscle | E | 616.0 | Mid |
| 286 | RYR2 | heart | E | 45.1 | Long |
| 287 | CHRM3 | brain | E | 5.0 | Long |
| 288 | WDR64 | testis | P | 3.3 | Long |
| 289 | PLD5 | brain | E | 4.5 | Long |
| 290 | NLRP3 | spleen | E | 3.4 | Mid |
| 291 | OR2L3 others | brain | E | 2.6 | Mid |

Chr2: All database tissue targeted genes on chromosome 2

| Index | Gene | Tissue | TSE/TSP | Exp | Length |
| --- | --- | --- | --- | --- | --- |
| 1 | FAM110C | esophagus | E | 20.8 | Short |
| 2 | LINC01115 | pituitary | low | 0.2 | Mid |
| 3 | TPO | thyroid | P | 499.9 | Long |
| 4 | MYT1L | brain | P | 14.0 | Long |
| 5 | LINC01250 | pituitary | low | 0.7 | Long |
| 6 | DCDC2C | testis | P | 4.7 | Long |
| 7 | LINC01105 | brain | E | 4.3 | Mid |
| 8 | LINC00487 | testis | low | 0.7 | Mid |
| 9 | LINC00298 | brain | E | 0.2 | Long |
| 10 | NTSR2 | brain | P | 62.6 | Short |
| 11 | MYCNOS | testis | P | 1.7 | Mid |
| 12 | APOB | liver | E | 206.5 | Mid |
| 13 | OTOF | brain | P | 11.5 | Long |
| 14 | C2orf70 | testis | P | 25.0 | Mid |
| 15 | CIB4 | testis | P | 12.8 | Mid |
| 16 | KCNK3 | adrenal | E | 126.5 | Mid |
| 17 | SLC30A3 | testis | E | 31.3 | Short |
| 18 | DNAJC5G | testis | P | 21.2 | Short |
| 19 | TRIM54 | muscle | E | 219.3 | Mid |
| 20 | C2orf71 | testis | low | 0.4 | Short |
| 21 | CAPN13 | sm intestine | E | 17.8 | Mid |
| 22 | GALNT14 | kidney | E | 11.6 | Long |
| 23 | CAPN14 | esophagus | P | 124.0 | Mid |
| 24 | SRD5A2 | liver | E | 13.3 | Mid |
| 25 | VIT | nerve | E | 37.5 | Long |
| 26 | QPCT | adrenal | E | 91.1 | Mid |
| 27 | EPCAM | colon | E | 299.7 | Mid |
| 28 | KCNK12 | nerve | E | 15.9 | Mid |
| 29 | FSHR | testis | low | 1.3 | Long |
| 30 | NRXN1 | brain | P | 15.0 | Long |
| 31 | LINC01122 | brain | E | 0.7 | Long |
| 32 | LINC00309 | testis | low | 0.1 | Mid |
| 33 | PLEK | spleen | E | 57.3 | Mid |
| 34 | PROKR1 | testis | E | 0.9 | Short |
| 35 | BMP10 | heart | P | 234.5 | Short |
| 36 | GKN2 | stomach | E | 137.8 | Short |
| 37 | GKN1 | stomach | P | 1772.1 | Short |
| 38 | TGFA | brain | E | 11.0 | Long |
| 39 | ADD2 | brain | P | 22.5 | Long |
| 40 | FIGLA | testis | low | 0.4 | Short |
| 41 | CLEC4F | spleen | E | 2.1 | Short |
| 42 | CD207 | skin | E | 14.0 | Short |
| 44 | CYP26B1 | brain | E | 27.3 | Mid |
| 46 | C2orf78 | testis | P | 13.1 | Mid |
| 47 | LRRTM4 | brain | E | 3.5 | Long |
| 49 | REG3G | pancreas | E | 574.6 | Short |
| 50 | REEP1 | testis | E | 28.9 | Long |
| 51 | CD8A | spleen | E | 49.5 | Mid |
| 52 | CD8B | sm intestine | E | 7.8 | Mid |
| 54 | RGPD2 | testis | E | 5.1 | Mid |
| 55 | PLGLB2 | liver | P | 28.7 | Short |
| 56 | SMYD1 | heart | E | 86.0 | Mid |
| 57 | FABP1 | liver | E | 469.8 | Short |
| 59 | FOXI3 | placenta | P | 3.1 | Short |
| 60 | TEX37 | testis | P | 87.0 | Short |
| 64 | FAM95A | testis | E | 8.0 | Short |
| 66 | MAL | esophagus | E | 965.0 | Mid |
| 68 | TRIM43 | testis | low | 0.1 | Short |
| 70 | NEURL3 | pancreas | E | 7.1 | Short |
| 71 | ZAP70 | spleen | E | 32.5 | Mid |
| 72 | CNGA3 | pituitary | E | 9.8 | Mid |
| 73 | MGAT4A | sm intestine | E | 15.1 | Long |
| 74 | KIAA1211L | brain | E | 19.7 | Long |
| 75 | NMS | brain | low | Low | Short |
| 76 | IL1R2 | spleen | E | 53.1 | Mid |
| 77 | IL18R1 | lung | E | 18.5 | Mid |
| 78 | LINC01102 | brain | E | 3.0 | Mid |
| 79 | LINC01103 | brain | E | 0.1 | Mid |
| 80 | C2orf40 | artery | E | 42.6 | Short |
| 82 | ST6GAL2 | artery | E | 16.2 | Mid |
| 83 | RGPD4 | testis | P | 2.9 | Mid |
| 84 | SLC5A7 | colon | E | 1.3 | Mid |
| 85 | SULT1C3 | sm intestine | P | 1.6 | Mid |
| 86 | SULT1C2 | kidney | E | 35.2 | Mid |
| 87 | EDAR | esophagus | E | 2.2 | Mid |
| 88 | RGPD5 | testis | E | 0.4 | Mid |
| 89 | LIMS3 | artery | E | 0.1 | Mid |
| 90 | LINC01123 | brain | E | 3.7 | Short |
| 91 | LINC01106 | brain | E | 8.0 | Short |
| 92 | RGPD6 | testis | low | 0.1 | Mid |
| 93 | ACOXL | bladder | E | 2.0 | Mid |
| 94 | RGPD8 | testis | E | 3.1 | Mid |
| 95 | IL37 | skin | P | 39.5 | Short |
| 96 | IL36B | skin | P | 3.1 | Mid |
| 97 | IL1RN | esophagus | E | 700.2 | Mid |
| 98 | PSD4 | spleen | E | 16.4 | Mid |
| 99 | DPP10 | brain | E | 3.5 | Long |
| 100 | MARCO | lung | E | 56.5 | Mid |
| 101 | TMEM37 | sm intestine | E | 36.1 | Short |
| 102 | SCTR | pancreas | P | 41.8 | Mid |
| 103 | CFAP221 | testis | E | 9.4 | Long |
| 104 | TFCP2L1 | salivary gland | E | 60.5 | Mid |
| 105 | CNTNAP5 | brain | P | 0.4 | Long |
| 106 | CYP27C1 | cervix | E | 3.1 | Mid |
| 108 | POTEF | testis | Low | 0.9 | Mid |
| 111 | POTEI | testis | P | 1.7 | Mid |
| 112 | CFC1B | pituitary | Low | 0.2 | Short |
| 113 | TISP43 | testis | P | 2.6 | Short |
| 114 | CFC1 | pituitary | E | 5.4 | Short |
| 115 | POTEJ | testis | Low | 0.5 | Mid |
| 116 | ARHGEF4 | brain | E | 29.1 | Long |
| 117 | POTEE | testis | P | 1.0 | Mid |
| 119 | LINC01087 | testis | P | 2.8 | Short |
| 120 | C2orf27B | pancreas | P | 0.4 | Short |
| 121 | TMEM163 | brain | E | 38.8 | Long |
| 122 | MAP3K19 | testis | P | 5.5 | Mid |
| 123 | THSD7B | artery | E | 1.2 | Long |
| 124 | NXPH2 | ovary | E | 1.8 | Long |
| 125 | NEB | muscle | P | 426.6 | Long |
| 126 | GALNT13 | brain | P | 14.8 | Long |
| 127 | KCNJ3 | brain | E | 34.7 | Long |
| 128 | CYTIP | spleen | E | 26.3 | Mid |
| 129 | UPP2 | kidney | E | 1.4 | Mid |
| 130 | SLC4A10 | brain | E | 16.4 | Long |
| 131 | KCNH7 | testis | P | 0.8 | Long |
| 132 | GRB14 | liver | E | 24.1 | Long |
| 133 | CSRNP3 | brain | E | 3.6 | Long |
| 134 | SCN1A | brain | E | 10.7 | Long |
| 135 | XIRP2 | muscle | E | 131.8 | Long |
| 136 | B3GALT1 | brain | E | 4.3 | Short |
| 137 | ABCB11 | liver | E | 6.5 | Long |
| 138 | DHRS9 | colon | E | 17.9 | Mid |
| 139 | LRP2 | thyroid | E | 10.8 | Long |
| 140 | MYO3B | testis | E | 0.8 | Long |
| 141 | ERICH2 | testis | P | 162.8 | Mid |
| 142 | GAD1 | brain | E | 28.7 | Mid |
| 143 | CHRNA1 | muscle | P | 23.4 | Mid |
| 144 | HOXD1 | brain | E | 7.1 | Short |
| 145 | PDE11A | prostate | E | 2.0 | Long |
| 146 | TTN | muscle | E | 204.7 | Long |
| 147 | CCDC141 | heart | P | 3.3 | Long |
| 148 | ZNF385B | brain | E | 12.1 | Long |
| 149 | SCHLAP1 | prostate | E | 0.4 | Long |
| 150 | CERKL | spleen | E | 13.2 | Long |
| 151 | NEUROD1 | brain | P | 128.3 | Short |
| 152 | ZSWIM2 | testis | P | 4.4 | Mid |
| 153 | DIRC1 | fibroblast | P | 2.0 | Mid |
| 154 | PCGEM1 | prostate | P | 0.5 | Mid |
| 155 | PLCL1 | heart | E | 9.2 | Long |
| 156 | FTCDNL1 | adrenal | E | 2.0 | Mid |
| 157 | CD28 | spleen | E | 2.9 | Mid |
| 158 | CTLA4 | spleen | E | 2.8 | Short |
| 159 | ICOS | spleen | E | 1.8 | Mid |
| 160 | GPR1-AS | placenta | P | 0.5 | Mid |
| 161 | DYTN | testis | E | Low | Mid |
| 162 | CPO | sm intestine | P | 48.8 | Mid |
| 163 | PTH2R | kidney | E | 2.4 | Mid |
| 164 | MAP2 | brain | E | 84.1 | Long |
| 165 | UNC80 | brain | E | 18.7 | Long |
| 166 | ACADL | thyroid | E | 22.1 | Mid |
| 167 | MYL1 | muscle | P | 1479.9 | Short |
| 168 | ERBB4 | brain | E | 5.6 | Long |
| 169 | ABCA12 | skin | P | 13.3 | Long |
| 170 | RUFY4 | spleen | E | 1.3 | Mid |
| 171 | CXCR2 | spleen | E | 17.8 | Short |
| 172 | WNT10A | skin | E | 3.8 | Short |
| 173 | CCDC108 | testis | E | 7.3 | Mid |
| 174 | RESP18 | brain | P | 25.2 | Short |
| 175 | DES | heart | E | 6616.6 | Short |
| 176 | PAX3 | salivary gland | E | 1.9 | Mid |
| 177 | SGPP2 | esophagus | E | 14.0 | Long |
| 178 | MOGAT1 | liver | E | 1.4 | Mid |
| 179 | FAM124B | adipose | E | 2.6 | Mid |
| 180 | NYAP2 | brain | E | 0.3 | Long |
| 181 | COL4A4 | kidney | E | 10.4 | Long |
| 182 | C2orf83 | testis | P | 0.3 | Mid |
| 183 | SPHKAP | brain | E | 33.0 | Long |
| 184 | SP140 | spleen | E | 14.6 | Mid |
| 185 | GPR55 | testis | E | 3.7 | Mid |
| 186 | C2orf72 | liver | E | 60.5 | Short |
| 187 | TMEM25 | brain | E | 30.6 | Short |
| 188 | ECEL1 | ovary | E | 53.2 | Short |
| 189 | PRSS56 | testis | E | 1.9 | Short |
| 190 | INPP5D | spleen | E | 57.3 | Long |
| 191 | SAG | testis | E | 3.1 | Mid |
| 193 | MROH2A | testis | P | 2.5 | Mid |
| 194 | TRPM8 | prostate | E | 7.0 | Long |
| 195 | SPP2 | liver | P | 42.9 | Mid |
| 196 | ASB18 | heart | P | 1.3 | Mid |
| 197 | IQCA1 | thyroid | E | 14.1 | Long |
| 198 | ACKR3 | artery | E | 59.7 | Short |
| 199 | RAB17 | liver | E | 59.4 | Mid |
| 200 | ESPNL | brain | E | 2.8 | Mid |
| 201 | OR6B3 | nerve | P | 3.1 | Short |
| 202 | KIF1A | brain | E | 156.3 | Long |
| 203 | AGXT | liver | P | 1079.2 | Short |
| 204 | C2orf54 | esophagus | E | 99.8 | Short |
| 205 | ANO7 | prostate | E | 46.4 | Short |
| 206 | PDCD1 | spleen | E | 5.5 | Short |

Chr3: All database tissue targeted genes on chromosome 3

| Index | Gene | Tissue | TSE/TSP | Exp | Length |
| --- | --- | --- | --- | --- | --- |
| 1 | CNTN6 | brain | E | 6.8 | Long |
| 2 | CNTN4 | artery | E | 8.8 | Short |
| 3 | IL5RA | pituitary | low | 0.5 | Mid |
| 4 | LRRN1 | kidney | E | 7.2 | Mid |
| 5 | GRM7 | brain | E | 2.2 | Long |
| 6 | LHFPL4 | brain | E | 5.4 | Mid |
| 7 | SLC6A11 | brain | E | 19.4 | Long |
| 8 | SLC6A1 | brain | E | 73.5 | Mid |
| 9 | SYN2 | brain | E | 72.7 | Long |
| 10 | TIMP4 | adipose | E | 119.1 | Short |
| 11 | TMEM40 | esophagus | E | 49.8 | Mid |
| 12 | NUP210 | sm intestine | E | 13.4 | Long |
| 13 | WNT7A | brain | E | 2.2 | Mid |
| 15 | FGD5 | adipose | E | 15.9 | Short |
| 16 | DAZL | testis | P | 34.1 | Mid |
| 17 | KCNH8 | pituitary | E | 12.7 | Long |
| 18 | ZNF385D | artery | E | 8.3 | Long |
| 19 | LINC00692 | testis | P | 4.8 | Mid |
| 20 | LRRC3B | brain | E | 5.7 | Mid |
| 21 | GADL1 | esophagus | E | 0.3 | Long |
| 22 | TRIM71 | testis | P | 4.1 | Mid |
| 23 | ARPP21 | brain | E | 136.3 | Long |
| 24 | STAC | artery | E | 6.7 | Long |
| 25 | DCLK3 | nerve | E | 14.3 | Mid |
| 26 | ITGA9 | thyroid | E | 15.7 | Long |
| 27 | SCN11A | spleen | E | 1.4 | Long |
| 28 | CX3CR1 | brain | E | 6.2 | Mid |
| 29 | MOBP | brain | P | 579.9 | Mid |
| 30 | MYRIP | brain | E | 15.3 | Long |
| 31 | LYZL4 | testis | P | 47.0 | Short |
| 32 | TOPAZ1 | testis | E | 2.9 | Mid |
| 33 | SLC6A20 | sm intestine | E | 8.3 | Mid |
| 34 | CCR3 | skin | E | 0.1 | Long |
| 35 | LTF | salivary gland | E | 331.9 | Mid |
| 36 | BSN | brain | E | 25.6 | Long |
| 37 | CACNA2D2 | lung | E | 16.8 | Long |
| 38 | ITIH1 | liver | P | 329.0 | Short |
| 39 | CACNA1D | pituitary | E | 4.9 | Long |
| 40 | CACNA2D3 | brain | E | 10.0 | Long |
| 41 | ERC2 | brain | E | 5.0 | Long |
| 42 | DNAH12 | testis | P | 3.6 | Long |
| 43 | DNASE1L3 | spleen | E | 52.9 | Mid |
| 44 | FAM3D | salivary gland | E | 132.8 | Mid |
| 45 | LINC00698 | predicted | Low | 0.1 | Long |
| 46 | FAM19A1 | brain | P | 6.0 | Long |
| 47 | FAM19A4 | adrenal | E | 3.3 | Long |
| 48 | FRMD4B | thyroid | E | 11.8 | Long |
| 49 | LINC01212 | artery | Low | 0.2 | Mid |
| 50 | LINC00877 | testis | Low | 1.0 | Long |
| 51 | LINC00971 | testis | Low | 0.3 | Long |
| 52 | CADM2 | brain | E | 20.2 | Short |
| 53 | EPHA3 | prostate | E | 8.8 | Long |
| 54 | LINC00879 | testis | P | 1.8 | Mid |
| 56 | EPHA6 | testis | P | 3.5 | Long |
| 57 | GABRR3 | testis | Low | 0.1 | Mid |
| 58 | OR5K4 | testis | Low | 0.1 | Short |
| 59 | ADGRG7 | sm intestine | E | 10.6 | Mid |
| 60 | ZPLD1 | testis | E | 0.1 | Long |
| 61 | HHLA2 | sm intestine | E | 20.0 | Mid |
| 62 | MORC1 | testis | P | 7.7 | Long |
| 63 | BTLA | spleen | E | 3.6 | Mid |
| 64 | CD200R1 | spleen | E | 2.0 | Mid |
| 65 | SIDT1 | brain | E | 8.2 | Short |
| 66 | DRD3 | brain | E | 1.8 | Mid |
| 67 | PLA1A | lung | E | 12.9 | Mid |
| 68 | MAATS1 | pituitary | E | 25.5 | Mid |
| 69 | NR1I2 | liver | E | 23.8 | Mid |
| 70 | FBXO40 | heart | E | 30.2 | Mid |
| 71 | CD86 | spleen | E | 8.5 | Mid |
| 72 | CSTA | esophagus | E | 1481.1 | Mid |
| 73 | SEMA5B | brain | E | 5.0 | Long |
| 74 | ADCY5 | esophagus | E | 56.6 | Long |
| 75 | ROPN1 | testis | P | 40.8 | Mid |
| 76 | ROPN1B | testis | P | 60.1 | Short |
| 77 | UROC1 | liver | P | 47.4 | Mid |
| 78 | CHST13 | testis | E | 20.5 | Mid |
| 79 | KBTBD12 | muscle | E | 6.7 | Mid |
| 80 | DNAJB8 | testis | P | 41.2 | Short |
| 81 | KIAA1257 | testis | E | 2.9 | Mid |
| 82 | EFCC1 | lung | E | 8.8 | Mid |
| 83 | RHO | brain | Low | 0.1 | Short |
| 85 | COL6A5 | lung | E | 1.0 | Long |
| 86 | CPNE4 | prostate | E | 12.0 | Long |
| 87 | EPHB1 | brain | E | 12.6 | Long |
| 88 | BPESC1 | testis | Low | 0.1 | Mid |
| 89 | SPSB4 | testis | E | 7.0 | Mid |
| 90 | PLSCR1 | adipose | E | 27.2 | Mid |
| 91 | ZIC4 | brain | P | 76.4 | Mid |
| 92 | CPB1 | pancreas | P | 10,112.1 | Mid |
| 93 | CLRN1 | adrenal | E | 1.2 | Mid |
| 94 | C3orf79 | adipose | Low | 0.3 | Mid |
| 95 | GPR149 | brain | E | 1.2 | Mid |
| 96 | PLCH1 | testis | E | 10.8 | Long |
| 97 | KCNAB1 | artery | E | 35.9 | Long |
| 98 | IQCJ | brain | E | 4.8 | Long |
| 99 | SCHIP1 | muscle | E | 4.0 | Mid |
| 100 | SPTSSB | skin | E | 20.6 | Mid |
| 101 | OTOL1 | brain | Low | 0.7 | Short |
| 102 | LINC01192 | testis | P | 2.3 | Long |
| 103 | SI | sm intestine | E | 75.1 | Mid |
| 104 | BCHE | esophagus | E | 14.9 | Mid |
| 105 | ZBBX | testis | E | 16.3 | Long |
| 106 | LRRC31 | colon | E | 6.5 | Mid |
| 107 | SAMD7 | testis | Low | 0.1 | Mid |
| 108 | SLC2A2 | liver | E | 88.8 | Mid |
| 109 | GHSR | pituitary | P | 2.0 | Short |
| 110 | SPATA16 | testis | P | 41.2 | Long |
| 111 | LINC01208 | testis | P | 2.4 | Mid |
| 112 | LINC01209 | testis | P | 0.8 | Short |
| 113 | LINC01014 | pituitary | E | 0.8 | Mid |
| 114 | KCNMB2 | ovary | E | 2.4 | Long |
| 115 | PEX5L | brain | E | 16.4 | Long |
| 116 | FLJ46066 | testis | Low | 0.1 | Mid |
| 117 | LAMP3 | lung | E | 48.1 | Mid |
| 118 | KLHL6 | am intestine | E | 4.0 | Mid |
| 119 | HTR3D | testis | Low | 0.1 | Short |
| 120 | AHSG | liver | P | 826.4 | Short |
| 121 | FETUB | liver | E | 29.9 | Mid |
| 122 | HRG | liver | P | 361.4 | Short |
| 123 | ADIPOQ | adipose | E | 224.7 | Mid |
| 124 | TP63 | prostate | E | 8.4 | Long |
| 125 | TMEM207 | kidney | Low | 0.8 | Mid |
| 126 | GMNC | fallopian tube | E | 2.1 | Short |
| 127 | ATP13A5 | skin | E | 1.7 | Long |
| 128 | ATP13A4 | thyroid | E | 23.2 | Long |
| 129 | MUC4 | colon | E | 25.4 | Mid |

Chr4: All database tissue targeted genes on chromosome 4

| Index | Gene | Tissue | TSE/TSP | Exp | Length |
| --- | --- | --- | --- | --- | --- |
| 1 | PDE6B | brain | E | 9.0 | Mid |
| 2 | RNF212 | pituitary | E | 5.7 | Mid |
| 3 | GRK4 | testis | E | 27.1 | Mid |
| 4 | DOK7 | heart | E | 13.4 | Mid |
| 5 | LINC00955 | sm intestine | P | 4.1 | Short |
| 6 | JAKMIP1 | brain | E | 33.9 | Long |
| 7 | PPP2R2C | brain | E | 43.9 | Long |
| 8 | ABLIM2 | muscle | E | 61.8 | Long |
| 9 | GPR78 | esophagus | P | 2.7 | Short |
| 12 | HS3ST1 | ovary | E | 14.2 | Mid |
| 14 | CD38 | spleen | E | 11.0 | Mid |
| 15 | PROM1 | salivary gland | E | 19.7 | Long |
| 16 | GBA3 | sm intestine | E | 27.4 | Long |
| 17 | LGI2 | nerve | E | 8.1 | Mid |
| 18 | SLC34A2 | lung | E | 231.3 | Mid |
| 19 | CCKAR | stomach | P | 7.7 | Short |
| 20 | DTHD1 | lung | Low | 0.1 | Mid |
| 21 | NWD2 | brain | Low | 1.0 | Long |
| 22 | KLB | liver | E | 5.1 | Mid |
| 23 | RHOH | spleen | E | 10.7 | Mid |
| 24 | CHRNA9 | skin | Low | 0.2 | Mid |
| 25 | NSUN7 | testis | E | 11.9 | Mid |
| 26 | UCHL1-AS1 | pituitary | E | 2.5 | Mid |
| 27 | PHOX2B | colon | E | 1.6 | Short |
| 28 | BEND4 | testis | E | 5.2 | Mid |
| 29 | ATP8A1 | thyroid | E | 26.6 | Long |
| 30 | GRXCR1 | testis | Low | 0.1 | Long |
| 31 | KCTD8 | brain | E | 12.0 | Long |
| 32 | YIPF7 | muscle | E | 5.5 | Mid |
| 33 | GABRG1 | brain | P | 13.7 | Mid |
| 34 | GABRA2 | brain | P | 6.1 | Long |
| 35 | COX7B2 | testis | P | 39.0 | Long |
| 36 | TXK | spleen | E | 5.3 | Mid |
| 37 | SLC10A4 | pituitary | E | 5.7 | Short |
| 38 | CWH43 | skin | E | 17.2 | Mid |
| 39 | KDR | thyroid | E | 31.9 | Mid |
| 40 | PDCL2 | testis | P | 67.5 | Mid |
| 41 | NMU | esophagus | E | 44.2 | Mid |
| 42 | KIAA1211 | testis | E | 20.7 | Long |
| 43 | HOPX | skin | E | 351.7 | Mid |
| 44 | SPINK2 | testis | P | 264.2 | Short |
| 45 | TECRL | heart | P | 87.1 | Long |
| 46 | EPHA5 | brain | P | 2.5 | Long |
| 47 | STAP1 | spleen | E | 12.5 | Mid |
| 48 | TMPRSS11F | esophagus | E | 6.3 | Mid |
| 50 | TMPRSS11B | esophagus | E | 260.9 | Mid |
| 51 | TMPRSS11E | esophagus | E | 133.1 | Mid |
| 52 | UGT2B17 | colon | E | 159.4 | Mid |
| 53 | UGT2B15 | liver | E | 54.5 | Mid |
| 54 | UGT2B10 | liver | P | 71.1 | Mid |
| 55 | UGT2A3 | sm intestine | E | 30.8 | Mid |
| 57 | SULT1B1 | colon | E | 14.8 | Mid |
| 58 | SULT1E1 | skin | E | 10.9 | Mid |
| 59 | CSN1S1 | adipose | E | 2.2 | Mid |
| 60 | ODAM | salivary gland | E | 258.2 | Short |
| 61 | FDSCP | salivary gland | P | 2729.0 | Short |
| 62 | SMR3A | thyroid | Low | 0.5 | Short |
| 63 | AMBN | brain | P | 1.0 | Mid |
| 64 | ENAM | kidney | E | 1.3 | Mid |
| 65 | JCHAIN | salivary gland | E | 652.1 | Short |
| 66 | GC | liver | E | 649.5 | Mid |
| 67 | NPFFR2 | stomach | Low | 0.1 | Long |
| 68 | AFM | liver | P | 153.4 | Mid |
| 69 | CXCL6 | spleen | E | 36.2 | Short |
| 70 | AREG | lung | E | 3.9 | Short |
| 71 | BTC | colon | E | 17.4 | Mid |
| 72 | C4orf26 | brain | Low | 0.2 | Short |
| 73 | PPEF2 | testis | Low | 0.6 | Mid |
| 74 | ART3 | muscle | E | 44.3 | Mid |
| 75 | CXCL13 | spleen | P | 273.4 | Long |
| 76 | FRAS1 | thyroid | E | 5.5 | Long |
| 77 | LINC01088 | artery | E | 7.9 | Long |
| 78 | LINC00989 | adipose | E | 2.9 | Long |
| 79 | PCAT4 | prostate | P | 8.3 | Mid |
| 80 | TMEM150C | adrenal | E | 28.1 | Mid |
| 81 | PLAC8 | spleen | E | 22.7 | Mid |
| 82 | HPSE | esophagus | E | 4.4 | Mid |
| 83 | CDS1 | esophagus | E | 13.6 | Mid |
| 84 | DSPP | testis | Low | 0.1 | Short |
| 85 | MEPE | brain | P | 2.3 | Mid |
| 87 | HERC5 | testis | E | 42.0 | Mid |
| 88 | GPRIN3 | brain | E | 9.2 | Mid |
| 89 | MMRN1 | thyroid | E | 19.6 | Mid |
| 90 | CCSER1 | testis | E | 1.7 | Long |
| 91 | GRID2 | brain | E | 7.0 | Long |
| 92 | HPGDS | lung | E | 2.8 | Mid |
| 93 | UNC5C | thyroid | E | 4.6 | Long |
| 94 | ADH7 | esophagus | P | 91.5 | Mid |
| 95 | C4orf17 | testis | P | 8.5 | Mid |
| 96 | MTTP | sm intestine | E | 28.8 | Mid |
| 97 | DAPP1 | esophagus | E | 17.6 | Mid |
| 98 | EMCN | thyroid | E | 15.4 | Long |
| 99 | LINC01216 | testis | Low | 0.2 | Short |
| 100 | SLC39A8 | lung | E | 36.1 | Mid |
| 101 | TACR3 | brain | Low | 0.2 | Long |
| 102 | CXXC4 | brain | E | 4.7 | Mid |
| 103 | ARHGEF38 | salivary gland | E | 3.1 | Short |
| 104 | NPNT | thyroid | E | 108.6 | Mid |
| 106 | DKK2 | nerve | E | 3.3 | Long |
| 107 | LEF1 | testis | E | 9.6 | Long |
| 108 | ETNPPL | brain | E | 127.2 | Mid |
| 109 | COL25A1 | testis | E | 12.0 | Long |
| 110 | CFI | liver | E | 161.5 | Mid |
| 111 | ANK2 | brain | E | 40.2 | Long |
| 112 | UGT8 | brain | E | 93.7 | Mid |
| 113 | NDST4 | prostate | e | 0.1 | Long |
| 114 | NDNF | lung | E | 17.6 | Mid |
| 115 | TNIP3 | testis | Low | 0.4 | Mid |
| 116 | QRFPR | kidney | Low | 0.8 | Mid |
| 117 | ADAD1 | testis | P | 67.0 | Mid |
| 118 | IL2 | sm intestine | Low | 0.3 | Short |
| 119 | IL21 | testis | Low | 1.3 | Short |
| 120 | LINC00613 | ? | Low | 0.1 | Mid |
| 121 | LINC00499 | brain | Low | 1.0 | Long |
| 122 | GYPB | ? | Low | 0.1 | Mid |
| 123 | GYPA | ? | Low | 0.1 | Mid |
| 124 | C4orf51 | testis | P | 5.6 | Mid |
| 125 | LINC01095 | testis | P | 12.3 | Short |
| 126 | TTC29 | testis | P | 36.7 | Long |
| 127 | FAM160A1 | skin | E | 5.1 | Long |
| 128 | TMEM154 | skin | E | 12.4 | Mid |
| 129 | TLR2 | spleen | E | 31.4 | Mid |
| 130 | RNF175 | brain | E | 7.3 | Mid |
| 131 | SFRP2 | breast | E | 104.5 | Short |
| 132 | DCHS2 | testis | E | 1.1 | Long |
| 133 | FGB | liver | P | 4876.7 | Short |
| 134 | RBM46 | testis | P | 8.1 | Mid |
| 135 | TDO2 | liver | P | 122.5 | Mid |
| 136 | GRIA2 | brain | E | 32.4 | Long |
| 137 | RXFP1 | brain | E | 4.3 | Long |
| 138 | C4orf45 | testis | P | 10.2 | Long |
| 139 | FSTL5 | brain | P | 38.0 | Long |
| 140 | LINC01207 | sm intestine | E | 3.8 | Mid |
| 141 | APELA | prostate | Low | 0.5 | Mid |
| 142 | TLL1 | brain | E | 5.8 | Long |
| 143 | SPOCK3 | brain | P | 48.0 | Long |
| 144 | GLRA3 | brain | P | 1.0 | Long |
| 145 | ADAM29 | testis | P | 20.7 | Mid |
| 146 | GPM6A | brain | E | 116.7 | Long |
| 147 | LINC01098 | testis | P | 1.7 | Long |
| 148 | LINC01099 | testis | Low | 0.2 | Long |
| 149 | LINC00290 | brain | P | 1.2 | Mid |
| 150 | ENPP6 | brain | E | 14.0 | Long |
| 151 | SORBS2 | heart | E | 44.7 | Long |
| 152 | TLR3 | adrenal | E | 2.2 | Mid |
| 153 | F11 | liver | E | 47.8 | Short |
| 154 | LINC01060 | ? | Low | 0.1 | Long |

Chr5: All database tissue targeted genes on chromosome 5

| Index | Gene | Tissue | TSE/TSP | Exp | Length |
| --- | --- | --- | --- | --- | --- |
| 1 | NKD2 | lung | E | 31.4 | Mid |
| 2 | SLC6A19 | sm intestine | E | 96.6 | Mid |
| 3 | TERT | testis | E | 1.3 | Mid |
| 4 | SLC6A3 | brain | P | 34.4 | Mid |
| 5 | IRX4 | skin | E | 13.2 | Short |
| 6 | LINC01019 | testis | P | 1.7 | Long |
| 7 | LINC01020 | testis | P | 1.0 | Mid |
| 8 | ADAMTS16 | ovary | E | 6.0 | Long |
| 9 | FLJ33360 | brain | E | 1.7 | Mid |
| 10 | UBE2QL1 | brain | E | 34.6 | Mid |
| 11 | ADCY2 | brain | E | 18.0 | Long |
| 12 | C5orf49 | testis | E | 26.5 | Mid |
| 13 | CTNND2 | brain | P | 52.0 | Long |
| 14 | LINC01194 | testis | P | 0.2 | Long |
| 15 | MARCH11 | testis | E | 47.1 | Long |
| 17 | CDH18 | brain | E | 10.0 | Long |
| 18 | CDH12 | pituitary | E | 1.1 | Long |
| 19 | PRDM9 | testis | P | 3.8 | Long |
| 20 | CDH10 | brain | E | 16.1 | Long |
| 21 | CDH9 | brain | P | 3.5 | Long |
| 22 | PDZD2 | nerve | E | 13.0 | Long |
| 23 | TTC23L | testis | E | 10.8 | Mid |
| 24 | AGXT2 | liver | E | 34.9 | Mid |
| 25 | PRLR | placenta | E | 18.9 | Long |
| 26 | UGT3A1 | testis | E | 5.4 | Mid |
| 27 | UGT3A2 | skin | E | 4.6 | Mid |
| 28 | EGFLAM | breast | E | 9.4 | Short |
| 29 | FYB | spleen | E | 7.2 | Long |
| 30 | C9 | liver | P | 258.4 | Mid |
| 31 | MROH2B | testis | P | 24.3 | Mid |
| 32 | PLCXD3 | heart | E | 11.9 | Long |
| 33 | CCL28 | salivary gland | E | 89.8 | Mid |
| 34 | FGF10 | cervix | E | 9.2 | Mid |
| 35 | HCN1 | brain | E | 6.2 | Long |
| 36 | EMB | sm intestine | E | 20.8 | Mid |
| 37 | GZMA | spleen | E | 33.7 | Short |
| 38 | MCIDAS | stomach | E | 1.0 | Short |
| 39 | DDX4 | testis | P | 88.0 | Mid |
| 40 | RAB3C | brain | E | 6.1 | Long |
| 41 | ELOVL7 | brain | E | 16.4 | Mid |
| 42 | C5orf64 | brain | P | 1.9 | Mid |
| 44 | RGS7BP | brain | E | 0.6 | Long |
| 45 | CD180 | spleen | E | 18.2 | Mid |
| 48 | SERF1A | testis | E | 1.1 | Short |
| 50 | NAIP | spleen | E | 12.8 | Mid |
| 51 | ZNF366 | adipose | E | 3.0 | Mid |
| 52 | FAM169A | brain | E | 8.0 | Mid |
| 53 | SV2C | brain | P | 1.0 | Long |
| 54 | CRHBP | spleen | E | 15.5 | Mid |
| 55 | ANKRD34B | brain | P | 7.9 | Short |
| 56 | ACOT12 | liver | P | 23.2 | Mid |
| 57 | HAPLN1 | artery | P | 2.3 | Mid |
| 59 | LINC00461 | brain | P | 7.9 | Long |
| 60 | ADGRV1 | adrenal | E | 29.0 | Long |
| 61 | MCTP1 | brain | E | 6.0 | Long |
| 62 | FAM81B | testis | P | 42.1 | Mid |
| 63 | LIX1 | brain | P | 7.3 | Mid |
| 65 | ST8SIA4 | spleen | E | 5.3 | Mid |
| 66 | SLCO4C1 | kidney | E | 1.8 | Mid |
| 67 | LINC00491 | testis | Low | 0.2 | Mid |
| 68 | EPB41L4A | thyroid | E | 4.5 | Mid |
| 69 | LINC00992 | testis | E | 1.3 | Long |
| 70 | MEGF10 | vagina | E | 4.5 | Long |
| 71 | KIAA1024L | brain | E | 0.1 | Mid |
| 72 | ACSL6 | brain | E | 18.0 | Mid |
| 73 | FSTL4 | brain | E | 3.2 | Long |
| 74 | CXCL14 | skin | E | 475.4 | Short |
| 75 | SLC25A48 | nerve | E | 12.1 | Mid |
| 77 | TRPC7 | testis | Low | 0.7 | Long |
| 78 | WNT8A | testis | Low | 0.8 | Short |
| 79 | PSD2 | brain | P | 50.5 | Mid |
| 80 | NRG2 | brain | E | 8.3 | Long |
| 81 | SLC4A9 | kidney | P | 14.8 | Short |
| 82 | PCDHA1 | pituitary | E | 0.5 | Long |
| 83 | GRXCR2 | testis | low | 0.8 | Short |
| 84 | PPP2R2B | brain | E | 14.9 | Long |
| 85 | STK32A | brain | E | 7.0 | Long |
| 86 | JAKMIP2 | brain | E | 9.9 | Long |
| 87 | SPINK1 | pancreas | E | 2884.9 | Short |
| 88 | SPINK5 | esophagus | E | 540.3 | Mid |
| 89 | HTR4 | sm intestine | E | 1.9 | Long |
| 90 | PDE6A | testis | E | 2.0 | Mid |
| 91 | SLC36A2 | muscle | E | 14.4 | Mid |
| 92 | TIMD4 | testis | E | 47.8 | Mid |
| 93 | HAVCR2 | spleen | E | 12.8 | Mid |
| 94 | ITK | spleen | E | 8.4 | Mid |
| 95 | SOX30 | testis | P | 64.1 | Mid |
| 96 | ATP10B | colon | E | 13.8 | Long |
| 97 | GABRB2 | brain | P | 32.1 | Long |
| 98 | GABRA6 | brain | P | 135.2 | Mid |
| 99 | GABRA1 | brain | P | 29.8 | Mid |
| 100 | TENM2 | heart | E | 17.0 | Long |
| 101 | SLIT3 | adipose | E | 40.0 | Long |
| 102 | LCP2 | spleen | E | 32.0 | Mid |
| 103 | KCNIP1 | brain | P | 14.4 | Long |
| 104 | C5orf47 | testis | P | 18.9 | Mid |
| 105 | HMP19 | brain | P | 195.5 | Mid |
| 107 | HRH2 | stomach | E | 2.2 | Mid |
| 108 | FAM153B | brain | E | 18.1 | Mid |
| 109 | UNC5A | brain | E | 29.9 | Mid |
| 110 | HK3 | spleen | E | 92.1 | Mid |
| 111 | SLC34A1 | kidney | P | 9.1 | Short |
| 112 | FAM153A | testis | E | 9.5 | Mid |
| 113 | FAM153C | brain | E | 15.6 | Mid |
| 114 | COL23A1 | thyroid | E | 42.7 | Long |
| 116 | RASGEF1C | brain | E | 20.5 | Long |
| 117 | FLT4 | thyroid | E | 24.5 | Mid |
| 118 | BTNL8 | sm intestine | E | 23.7 | Mid |
| 119 | BTNL3 | sm intestine | E | 41.9 | Short |
| 120 | BTNL9 | adipose | E | 137.2 | Mid |

Chr6: All database tissue targeted genes on chromosome 6

| Index | Gene | Tissue | TSE/TSP | Exp | Length |
| --- | --- | --- | --- | --- | --- |
| 1 | IRF4 | cervix | E | 13.0 | Mid |
| 2 | NRN1 | brain | E | 130.1 | Short |
| 3 | F13A1 | adipose | E | 53.1 | Long |
| 4 | LY86-AS1 | brain | P | 1.7 | Short |
| 5 | CAGE1 | testis | P | 6.7 | Mid |
| 6 | SYCP2L | testis | E | 4.0 | Mid |
| 7 | STMND1 | testis | E | 4.3 | Mid |
| 8 | LINC00581 | colon | P | 0.1 | Mid |
| 9 | PRL | pituitary | P | 29,492.8 | Short |
| 10 | NRSN1 | brain | E | 34.6 | Mid |
| 11 | DCDC2 | kidney | E | 13.4 | Long |
| 12 | GPLD1 | brain | E | 7.0 | Mid |
| 13 | SCGN | pituitary | E | 27.3 | Mid |
| 14 | SLC17A4 | liver | E | 11.1 | Mid |
| 15 | SLC17A1 | kidney | E | 26.1 | Mid |
| 16 | SLC17A3 | kidney | E | 13.2 | Mid |
| 17 | SLC17A2 | liver | P | 21.4 | Mid |
| 19 | GPX6 | testis | Low | 0.2 | Short |
| 20 | MOG | brain | P | 272.5 | Mid |
| 21 | TRIM40 | sm intestine | E | 1.8 | Short |
| 22 | TRIM15 | colon | E | 11.3 | Short |
| 23 | DPCR1 | stomach | E | 37.6 | Mid |
| 24 | MUC22 | esophagus | E | 0.8 | Mid |
| 25 | HCG23 | brain | E | 3.0 | Short |
| 26 | HLA-DRB5 | fallopian tube | E | 22.8 | Short |
| 27 | HLA-DQA1 | spleen | E | 30.4 | Short |
| 28 | HLA-DOA | spleen | E | 22.2 | Short |
| 29 | GRM4 | brain | P | 160.1 | Long |
| 30 | PACSIN1 | brain | P | 157.8 | Mid |
| 31 | SPDEF | prostate | E | 97.4 | Mid |
| 32 | TCP11 | testis | P | 214.6 | Mid |
| 33 | CLPSL1 | pancreas | P | 48.6 | Short |
| 34 | LHFPL5 | pancreas | E | 1.7 | Mid |
| 35 | C6orf222 | sm intestine | E | 6.2 | Mid |
| 36 | ETV7 | skin | E | 5.1 | Mid |
| 37 | RAB44 | lung | Low | 1.0 | Mid |
| 38 | CPNE5 | brain | E | 57.6 | Mid |
| 39 | FGD2 | spleen | E | 93.2 | Mid |
| 40 | DNAH8 | testis | P | 5.6 | Long |
| 41 | GLP1R | pancreas | E | 2.9 | Mid |
| 42 | KCNK5 | sm intestine | E | 23.3 | Mid |
| 43 | KCNK6 | vagina | E | 12.9 | Short |
| 44 | KIF6 | brain | E | 1.4 | Long |
| 45 | LRFN2 | brain | E | 4.3 | Long |
| 46 | TREML2 | spleen | E | 5.7 | Short |
| 47 | NCR2 | sm intestine | E | 0.2 | Mid |
| 48 | MDFI | esophagus | E | 16.1 | Mid |
| 49 | TTBK1 | brain | E | 12.6 | Mid |
| 50 | SLC22A7 | liver | E | 77.8 | Short |
| 51 | CAPN11 | testis | E | 32.3 | Mid |
| 52 | CLIC5 | muscle | E | 16.7 | Long |
| 54 | RCAN2 | artery | E | 122.9 | Long |
| 55 | TDRD6 | testis | E | 13.2 | Mid |
| 56 | MEP1A | sm intestine | E | 153.8 | Mid |
| 57 | ADGRF1 | kidney | E | 10.7 | Mid |
| 58 | ADGRF2 | skin | E | 2.1 | Mid |
| 59 | ADGRF4 | skin | E | 26.8 | Mid |
| 60 | OPN5 | testis | P | 3.5 | Mid |
| 61 | GLYATL3 | liver | Low | Low | Mid |
| 62 | RHAG | heart | E | 1.0 | Mid |
| 63 | CRISP2 | testis | P | 554.8 | Mid |
| 64 | CRISP3 | salivary gland | E | 1248.7 | Mid |
| 65 | CRISP1 | testis | P | 1.7 | Mid |
| 66 | TFAP2D | brain | Low | 0.3 | Mid |
| 67 | TFAP2B | heart | E | 3.5 | Mid |
| 68 | PKHD1 | kidney | E | 6.8 | Long |
| 69 | IL17A | lung | Low | Low | Short |
| 70 | GSTA1 | adrenal | E | 582.0 | Short |
| 71 | GCM1 | testis | E | 0.7 | Mid |
| 72 | MLIP | heart | E | 32.7 | Long |
| 73 | TINAG | kidney | E | 10.5 | Mid |
| 74 | FAM83B | skin | E | 14.9 | Mid |
| 75 | HCRTR2 | brain | Low | 0.1 | Long |
| 76 | GFRAL | adipose | Low | 0.1 | Mid |
| 77 | HMGCLL1 | brain | E | 3.1 | Long |
| 78 | BMP5 | bladder | E | 7.3 | Long |
| 79 | COL21A1 | artery | E | 20.0 | Long |
| 80 | KHDRBS2 | brain | E | 5.0 | Long |
| 81 | LGSN | liver | Low | 0.3 | Mid |
| 82 | BAI3 | brain | E | 14.0 | Long |
| 83 | COL19A1 | lymph node | E | 1.1 | Long |
| 84 | B3GAT2 | brain | P | 1.0 | Long |
| 85 | IMPG1 | brain | P | 1.3 | Long |
| 86 | MEI4 | testis | E | 2.2 | Long |
| 87 | LINC01621 | brain | E | 1.0 | Mid |
| 88 | SNAP91 | brain | E | 65.4 | Long |
| 89 | MRAP2 | artery | E | 65.7 | Mid |
| 90 | HTR1E | brain | E | 2.2 | Mid |
| 91 | GJB7 | pituitary | E | 3.1 | Mid |
| 92 | SPACA1 | testis | P | 43.3 | Mid |
| 93 | CNR1 | pituitary | E | 11.3 | Mid |
| 94 | GABRR1 | testis | E | 0.8 | Mid |
| 96 | ANKRD6 | brain | E | 9.0 | Long |
| 97 | CASC6 | brain | Low | 0.1 | Long |
| 98 | EPHA7 | esophagus | E | 26.4 | Long |
| 99 | TSG1 | prostate | Low | 0.2 | Mid |
| 100 | FUT9 | brain | E | 13.0 | Long |
| 101 | FHL5 | artery | E | 159.7 | Mid |
| 102 | KLHL32 | brain | P | 11.3 | Long |
| 103 | MCHR2 | brain | E | 2.2 | Mid |
| 104 | SIM1 | kidney | E | 2.5 | Mid |
| 105 | LIN28B | testis | P | 2.3 | Long |
| 106 | NR2E1 | brain | P | 12.2 | Mid |
| 107 | METTL24 | colon | E | 3.0 | Long |
| 108 | DDO | heart | E | 8.1 | Mid |
| 109 | SLC22A16 | testis | P | 8.4 | Mid |
| 110 | SLC16A10 | muscle | E | 5.5 | Long |
| 111 | FRK | artery | E | 3.0 | Long |
| 112 | GPRC6A | salivary gland | Low | 0.2 | Mid |
| 113 | RFX6 | stomach | E | 1.0 | Mid |
| 114 | VGLL2 | muscle | E | 22.9 | Short |
| 115 | ROS1 | lung | P | 7.6 | Long |
| 116 | SLC35F1 | brain | E | 13.0 | Long |
| 117 | CLVS2 | brain | P | 15.8 | Mid |
| 118 | TRDN | muscle | E | 168.8 | Long |
| 119 | HEY2 | artery | E | 45.8 | Short |
| 120 | RSPO3 | esophagus | E | 24.3 | Mid |
| 121 | C6orf58 | salivary gland | P | 335.2 | Short |
| 122 | THEMIS | brain | Low | 1.0 | Long |
| 123 | TAAR9 | brain | Low | Low | Short |
| 124 | VNN2 | spleen | E | 40.5 | Short |
| 125 | LINC00326 | testis | P | 5.6 | Long |
| 126 | LINC01010 | testis | P | 5.6 | Long |
| 127 | MYB | colon | E | 8.0 | Mid |
| 128 | IL20RA | skin | E | 16.6 | Mid |
| 129 | ARFGEF3 | brain | P | 20.0 | Long |
| 130 | ECT2L | ? | Low | Low | Long |
| 131 | TXLNB | muscle | E | 60.2 | Mid |
| 132 | NMBR | testis | Low | 1.0 | Short |
| 133 | STX11 | adipose | E | 17.4 | Mid |
| 134 | GRM1 | brain | P | 14.8 | Long |
| 135 | ADGB | testis | P | 4.6 | Long |
| 136 | PPP1R14C | skin | E | 70.0 | Long |
| 137 | IYD | thyroid | P | 147.2 | Mid |
| 138 | ESR1 | cervix | E | 25.8 | Long |
| 139 | OPRM1 | brain | E | 0.2 | Mid |
| 140 | NOX3 | brain | Low | 0.1 | Mid |
| 141 | C6orf99 | testis | P | 75.9 | Mid |
| 142 | FNDC1 | thyroid | E | 12.3 | Long |
| 143 | SLC22A2 | kidney | P | 8.4 | Mid |
| 144 | SLC22A3 | nerve | E | 38.1 | Long |
| 146 | LPA | liver | P | 11.5 | Long |
| 147 | PLG | liver | P | 201.8 | Mid |
| 148 | C6orf118 | testis | E | 7.4 | Mid |
| 149 | CCR6 | spleen | E | 7.3 | Mid |
| 150 | UNC93A | skin | E | 9.5 | Mid |
| 151 | TTLL2 | testis | P | 17.4 | Mid |
| 152 | TCP10 | testis | P | 3.0 | Short |
| 153 | FRMD1 | stomach | E | 7.8 | Mid |
| 154 | DACT2 | brain | E | 9.7 | Short |
| 155 | SMOC2 | artery | E | 223.8 | Long |

Chr7: All database tissue targeted genes on chromosome 7

| Index | Gene | Tissue | TSE/TSP | Exp | Length |
| --- | --- | --- | --- | --- | --- |
| 1 | UNCX | brain | P | 18.3 | Short |
| 2 | MMD2 | brain | E | 9.9 | Mid |
| 3 | RSPH10B | testis | P | 6.9 | Mid |
| 4 | GRID2IP | brain | E | 2.0 | Mid |
| 5 | COL28A1 | nerve | E | 22.1 | Long |
| 6 | ICA1 | testis | E | 21.7 | Long |
| 7 | NXPH1 | adrenal | E | 11.1 | Long |
| 8 | THSD7A | nerve | E | 4.0 | Long |
| 9 | VWDE | pituitary | E | 4.0 | Mid |
| 10 | SCIN | kidney | E | 19.6 | Mid |
| 11 | DGKB | brain | P | 9.0 | Long |
| 12 | AGMO | liver | E | 7.4 | Long |
| 13 | MEOX2 | nerve | E | 41.0 | Mid |
| 14 | LRRC72 | testis | P | 2.3 | Mid |
| 15 | AGR3 | sm intestine | E | 58.2 | Mid |
| 16 | TMEM196 | brain | E | 3.0 | Mid |
| 17 | MACC1 | esophagus | E | 3.8 | Mid |
| 18 | ABCB5 | breast | E | 1.0 | Long |
| 19 | DNAH11 | esophagus | E | 1.5 | Long |
| 20 | GPNMB | skin | E | 133.3 | Mid |
| 21 | SNX10 | brain | E | 35.8 | Mid |
| 22 | HOXA10 | uterus | E | 41.6 | Short |
| 23 | HOXA13 | cervix | E | 22.9 | Short |
| 24 | CPVL | spleen | E | 45.3 | Long |
| 25 | CHN2 | brain | E | 45.2 | Long |
| 26 | WIPF3 | ovary | E | 32.3 | Long |
| 27 | CRHR2 | pituitary | E | 3.0 | Mid |
| 28 | AQP1 | artery | E | 2.7 | Short |
| 29 | GHRHR | pituitary | P | 143.5 | Mid |
| 30 | ADCYAP1R1 | brain | E | 36.0 | Mid |
| 31 | CCDC129 | salivary gland | E | 1.0 | Long |
| 32 | PPP1R17 | brain | E | 14.8 | Mid |
| 33 | NPSR1 | brain | Low | 0.1 | Long |
| 34 | TBX20 | heart | E | 12.1 | Mid |
| 35 | AOAH | spleen | E | 31.7 | Long |
| 36 | ELMO1 | brain | E | 28.0 | Long |
| 37 | NME8 | testis | E | 6.7 | Mid |
| 38 | SFRP4 | cervix | E | 214.0 | Short |
| 39 | EPDR1 | brain | E | 55.7 | Long |
| 40 | TARP | prostate | E | 21.6 | Short |
| 41 | POU6F2 | brain | E | 1.0 | Long |
| 42 | GCK | prostate | E | 15.7 | Mid |
| 43 | NPC1L1 | liver | E | 17.5 | Mid |
| 44 | RAMP3 | lung | E | 81.1 | Mid |
| 45 | ADCY1 | brain | E | 25.0 | Long |
| 46 | IGFBP1 | liver | P | 198.9 | Short |
| 47 | SUN3 | testis | P | 30.1 | Mid |
| 48 | ABCA13 | thyroid | Low | 0.1 | Long |
| 49 | VWC2 | brain | E | 9.3 | Long |
| 50 | ZPBP | testis | P | 45.7 | Long |
| 51 | C7orf72 | testis | P | 3.6 | Mid |
| 52 | IKZF1 | spleen | E | 17.2 | Long |
| 53 | DDC | sm intestine | E | 28.9 | Long |
| 54 | COBL | brain | E | 21.2 | Long |
| 55 | LINC01446 | testis | P | 0.3 | Long |
| 56 | VSTM2A | brain | P | 13.7 | Mid |
| 58 | SEPT14 | testis | P | 12.1 | Mid |
| 59 | ZNF479 | testis | P | 2.4 | Mid |
| 60 | ZNF716 | testis | Low | 0.2 | Mid |
| 61 | ZNF727 | ovary | E | 1.5 | Mid |
| 62 | ZNF679 | testis | P | 1.1 | Mid |
| 64 | WBSCR17 | brain | E | 37.2 | Long |
| 65 | CALN1 | brain | P | 38.0 | Long |
| 66 | FKBP6 | testis | P | 37.6 | Mid |
| 67 | MLXIPL | liver | E | 244.9 | Mid |
| 68 | WBSCR28 | testis | P | 86.1 | Short |
| 69 | NCF1 | spleen | E | 47.8 | Mid |
| 72 | CCL26 | ovary | E | 7.7 | Mid |
| 73 | SRRM3 | adrenal | E | 3.7 | Mid |
| 74 | CD36 | adipose | E | 256.3 | Long |
| 75 | PCLO | brain | E | 13.7 | Long |
| 76 | SEMA3E | esophagus | E | 2.2 | Long |
| 77 | GRM3 | brain | P | 25.6 | Long |
| 78 | ABCB4 | liver | E | 18.1 | Mid |
| 79 | ADAM22 | brain | E | 34.9 | Long |
| 80 | STEAP2 | prostate | E | 37.5 | Short |
| 81 | ZNF804B | thyroid | P | 2.0 | Long |
| 82 | CALCR | brain | Low | 1.0 | Long |
| 83 | PPP1R9A | brain | E | 7.0 | Long |
| 84 | PON1 | liver | P | 100.3 | Mid |
| 85 | PON3 | liver | E | 83.7 | Long |
| 86 | ASB4 | adrenal | E | 22.4 | Mid |
| 88 | DLX5 | skin | E | 13.3 | Short |
| 89 | KPNA7 | stomach | Low | 0.1 | Mid |
| 90 | CYP3A4 | liver | E | 247.4 | Mid |
| 91 | CYP3A43 | liver | E | 2.4 | Mid |
| 92 | AZGP1 | salivary gland | E | 1371.6 | Short |
| 93 | COL26A1 | brain | E | 3.1 | Long |
| 94 | MYL10 | pituitary | Low | 0.1 | Short |
| 95 | LHFPL3 | brain | P | 13.0 | Long |
| 96 | PIK3CG | spleen | E | 2.6 | Mid |
| 97 | SLC26A3 | colon | E | 247.5 | Mid |
| 98 | PPP1R3A | muscle | E | 14.6 | Mid |
| 99 | TFEC | spleen | E | 4.5 | Mid |
| 100 | ASZ1 | testis | P | 11.1 | Mid |
| 101 | ANKRD7 | testis | P | 434.2 | Mid |
| 102 | KCND2 | brain | E | 21.3 | Long |
| 103 | PTPRZ1 | brain | E | 24.8 | Long |
| 104 | CADPS2 | brain | E | 72.0 | Long |
| 105 | SLC13A1 | sm intestine | E | 3.8 | Mid |
| 106 | ASB15 | heart | E | 6.1 | Mid |
| 107 | LMOD2 | muscle | P | 182.0 | Short |
| 108 | HYAL4 | testis | E | 1.3 | Mid |
| 109 | SPAM1 | testis | P | 8.3 | Mid |
| 110 | GRM8 | testis | E | 1.4 | Long |
| 111 | LEP | adipose | E | 101.0 | Mid |
| 112 | KCP | kidney | E | 3.0 | Mid |
| 113 | TSPAN33 | kidney | E | 63.9 | Mid |
| 114 | SMKR1 | testis | E | 157.9 | Short |
| 115 | CPA2 | pancreas | P | 7338.8 | Short |
| 116 | PLXNA4 | adipose | E | 6.0 | Long |
| 117 | STRA8 | testis | E | 1.3 | Mid |
| 118 | AKR1D1 | liver | P | 31.3 | Long |
| 119 | SVOPL | testis | E | 1.0 | Mid |
| 120 | ATP6V0A4 | kidney | E | 25.5 | Mid |
| 121 | TMEM213 | kidney | E | 34.7 | Short |
| 122 | CLEC2L | brain | P | 29.9 | Mid |
| 123 | RAB19 | pituitary | E | 1.1 | Mid |
| 124 | MGAM | sm intestine | E | 6.6 | Mid |
| 125 | EPHB6 | skin | E | 52.0 | Mid |
| 126 | TRPV5 | kidney | Low | 1.1 | Mid |
| 127 | CTAGE4 | testis | Low | 0.9 | Short |
| 128 | TPK1 | testis | E | 3.9 | Long |
| 129 | CNTNAP2 | brain | P | 18.0 | Long |
| 130 | C7orf33 | testis | Low | 0.2 | Mid |
| 131 | SSPO | brain | E | 0.3 | Mid |
| 132 | ACTR3C | liver | E | 2.0 | Mid |
| 133 | GBX1 | testis | P | 6.8 | Mid |
| 134 | ASB10 | muscle | P | 11.2 | Short |
| 135 | WDR86 | thyroid | E | 21.9 | Mid |
| 136 | GALNT5 | salivary gland | E | 5.0 | Mid |
| 137 | DPP6 | brain | E | 28.0 | Long |
| 138 | CNPY1 | brain | P | 16.8 | Mid |
| 139 | PTPRN2 | brain | E | 40.0 | Long |
| 140 | LINC00689 | esophagus | E | 3.2 | Mid |
| 141 | VIPR2 | colon | E | 2.2 | Long |
| 126a | KEL | testis | E | 36.4 | Mid |

Chr8: All database tissue targeted genes on chromosome 8

| Index | Gene | Tissue | TSE/TSP | Exp | Length |
| --- | --- | --- | --- | --- | --- |
| 1 | ERICH-AS1 | testis | P | 2.2 | Long |
| 2 | DLGAP2 | brain | E | 5.0 | Long |
| 3 | CSMD1 | brain | E | 2.3 | Long |
| 5 | SPAG11B | prostate | Low | 0.1 | Short |
| 7 | PRSS55 | testis | P | 13.4 | Mid |
| 8 | RP1L1 | skin | Low | 0.2 | Mid |
| 9 | C8orf74 | testis | P | 3.9 | Mid |
| 10 | FAM66D | brain | E | 0.8 | Mid |
| 11 | LINC00681 | skin | P | 1.6 | Mid |
| 12 | FGL1 | liver | E | 848.6 | Mid |
| 13 | SLC18A1 | sm intestine | Low | 0.7 | Mid |
| 14 | GFRA2 | thyroid | E | 9.3 | Long |
| 15 | PEBP4 | heart | E | 60.8 | Long |
| 16 | ADAM28 | stomach | E | 14.0 | Mid |
| 17 | ADAMDEC1 | sm intestine | E | 95.5 | Mid |
| 18 | ADAM7 | testis | P | 1.9 | Mid |
| 19 | EBF2 | nerve | E | 19.6 | Long |
| 20 | ADRA1A | liver | E | 8.4 | Mid |
| 21 | SCARA5 | bladder | E | 55.8 | Long |
| 22 | NUGGC | liver | E | 4.6 | Mid |
| 24 | FAM183CP | testis | P | 0.5 | Mid |
| 25 | TEX15 | testis | E | 8.1 | Mid |
| 26 | UNC5D | pituitary | E | 0.8 | Long |
| 29 | ADAM18 | testis | P | 6.8 | Long |
| 30 | ADAM12 | ovary | E | 3.5 | Long |
| 32 | EFCAB1 | testis | E | 6.7 | Short |
| 33 | OPRK1 | brain | P | 2.0 | Mid |
| 34 | XKR4 | nerve | E | 1.6 | Long |
| 35 | SDR16C5 | skin | E | 27.0 | Mid |
| 36 | CYP7A1 | liver | Low | 1.1 | Short |
| 37 | CA8 | brain | E | 4.6 | Mid |
| 38 | NKAIN3 | brain | E | 0.4 | Long |
| 40 | TCF24 | testis | E | 1.3 | Mid |
| 41 | PREX2 | thyroid | E | 6.6 | Long |
| 42 | SLCO5A1 | muscle | E | 2.0 | Long |
| 43 | EYA1 | pituitary | E | 10.8 | Long |
| 44 | KCNB2 | brain | E | 1.8 | Long |
| 45 | JPH1 | muscle | E | 73.0 | Mid |
| 46 | PI15 | fallopian tube | E | 20.8 | Mid |
| 47 | CASC9 | colon | Low | 0.1 | Long |
| 48 | STMN2 | brain | E | 298.3 | Mid |
| 49 | TPD52 | brain | E | 23.5 | Mid |
| 51 | RALYL | brain | P | 22.0 | Long |
| 52 | CA1 | colon | E | 126.4 | Mid |
| 53 | PSKH2 | kidney | P | 21.2 | Mid |
| 54 | ATP6V0D2 | kidney | P | 21.2 | Mid |
| 55 | SLC7A13 | testis | Low | 0.1 | Mid |
| 56 | CNGB3 | testis | Low | 1.4 | Long |
| 57 | CNBD1 | testis | P | 3.1 | Long |
| 58 | LINC00534 | brain | Low | 0.1 | Long |
| 59 | NECAB1 | brain | P | 9.0 | Long |
| 60 | SLC26A7 | thyroid | E | 154.2 | Long |
| 61 | FLJ46284 | testis | P | 0.8 | Mid |
| 62 | C8orf87 | testis | E | 0.2 | Mid |
| 63 | RGS22 | testis | E | 38.2 | Long |
| 64 | GRHL2 | skin | E | 16.9 | Long |
| 65 | NCALD | brain | E | 44.0 | Long |
| 66 | PKHD1L1 | thyroid | E | 28.5 | Long |
| 67 | CSMD3 | brain | E | 1.5 | Long |
| 68 | LINC00536 | testis | Low | 0.2 | Long |
| 69 | SLC30A8 | pancreas | E | 4.0 | Mid |
| 71 | ANXA13 | sm intestine | E | 21.6 | Mid |
| 72 | LINC00861 | spleen | E | 7.5 | Long |
| 73 | CASC8 | salivary gland | Low | 0.2 | Long |
| 74 | LINC00977 | spleen | Low | 0.3 | Mid |
| 75 | GSDMC | esophagus | E | 25.8 | Mid |
| 76 | ADCY8 | brain | E | 3.6 | Long |
| 78 | HHLA1 | testis | Low | 0.1 | Mid |
| 79 | KCNQ3 | brain | P | 11.8 | Long |
| 80 | LRRC6 | testis | E | 13.9 | Long |
| 81 | FAM135B | testis | E | 4.3 | Long |
| 82 | COL22A1 | pituitary | P | 65.6 | Long |
| 83 | KCNK9 | brain | P | 24.1 | Long |
| 84 | MROH5 | testis | P | 8.3 | Mid |
| 38a | TTPA | liver | E | 22.3 | Mid |

Chr9: All database tissue targeted genes on chromosome 9

| Index | Gene | Tissue | TSE/TSP | Exp | Length |
| --- | --- | --- | --- | --- | --- |
| 1 | DOCK8 | spleen | E | 22.9 | Long |
| 2 | DMRT1 | testis | P | 21.3 | Long |
| 3 | DMRT3 | testis | E | 6.5 | Mid |
| 5 | IL33 | artery | E | 31.0 | Mid |
| 6 | GLDC | kidney | E | 10.7 | Long |
| 7 | PTPRD | brain | E | 27.5 | Long |
| 8 | TYRP1 | skin | E | 17.5 | Mid |
| 9 | LINC01235 | breast | E | 2.1 | Mid |
| 11 | FREM1 | brain | E | 5.0 | Long |
| 12 | SH3GL2 | brain | E | 142.1 | Long |
| 13 | SLC24A2 | brain | P | 11.3 | Long |
| 15 | ELAVL2 | brain | E | 14.6 | Long |
| 16 | LINGO2 | uterus | E | 5.0 | Long |
| 17 | AQP7 | adipose | E | 82.6 | Mid |
| 18 | DNAI1 | testis | E | 23.2 | Mid |
| 19 | CNTFR | brain | E | 55.2 | Mid |
| 20 | KIAA1045 | brain | E | 37.6 | Mid |
| 21 | PAX5 | spleen | E | 8.9 | Long |
| 22 | FRMPD1 | testis | E | 7.5 | Mid |
| 23 | ANKRD18A | testis | E | 9.6 | Mid |
| 25 | ANKRD20A2 | testis | Low | 0.5 | Mid |
| 31 | FAM189A2 | thyroid | E | 56.2 | Mid |
| 32 | GDA | sm intestine | E | 19.8 | Long |
| 34 | RORB | brain | E | 8.0 | Long |
| 35 | TRPM6 | colon | Low | 1.0 | Long |
| 36 | RASEF | pancreas | E | 8.5 | Mid |
| 37 | NTRK2 | brain | E | 49.4 | Long |
| 39 | SYK | spleen | E | 35.5 | Mid |
| 40 | FGD3 | spleen | E | 31.0 | Mid |
| 41 | WNK2 | heart | E | 27.4 | Long |
| 42 | FBP2 | muscle | E | 65.5 | Mid |
| 43 | HSD17B3 | testis | E | 17.6 | Mid |
| 44 | PLPPR1 | brain | P | 10.0 | Long |
| 47 | CYLC2 | testis | P | 46.3 | Mid |
| 48 | TXNDC8 | testis | P | 5.2 | Mid |
| 50 | TNFSF8 | spleen | E | 4.3 | Mid |
| 52 | BRINP1 | brain | P | 24.3 | Long |
| 54 | MORN5 | testis | E | 31.1 | Mid |
| 55 | LHX6 | adipose | E | 7.0 | Mid |
| 56 | LHX2 | brain | E | 31.1 | Mid |
| 57 | NR5A1 | spleen | E | 106.6 | Mid |
| 58 | LMX1B | breast | E | 1.5 | Mid |
| 60 | PRDM12 | brain | Low | 0.3 | Mid |
| 61 | LAMC3 | cervix | E | 23.8 | Mid |
| 62 | FAM78A | spleen | E | 14.5 | Mid |
| 63 | GFI1B | testis | E | 2.0 | Mid |
| 64 | ABO | sm intestine | E | 11.2 | Mid |
| 65 | TMEM8C | nerve | Low | 0.2 | Short |
| 66 | FCN1 | spleen | E | 60.8 | Short |
| 67 | OLFM1 | brain | E | 178.8 | Mid |
| 68 | GLT6D1 | testis | P | 2.2 | Mid |
| 69 | KCNT1 | brain | E | 58.9 | Mid |
| 70 | GRIN1 | brain | P | 121.1 | Mid |
| 71 | CACNA1B | brain | E | 19.6 | Long |

Chr10: All database tissue targeted genes on chromosome 10

| Index | Gene | Tissue | TSE/TSP | Exp | Length |
| --- | --- | --- | --- | --- | --- |
| 1 | ADARB2 | brain | E | 5.0 | Long |
| 2 | UCN3 | pituitary | E | 1.4 | Short |
| 3 | IL2RA | spleen | E | 5.8 | Mid |
| 4 | ITIH5 | adipose | E | 41.2 | Long |
| 5 | GATA3-AS1 | kidney | E | 1.7 | Short |
| 6 | OLAH | testis | E | 15.9 | Mid |
| 7 | ITGA8 | artery | E | 237.3 | Long |
| 8 | TMEM236 | sm intestine | E | 0.5 | Mid |
| 9 | SLC39A12 | brain | P | 18.0 | Mid |
| 10 | NEBL | heart | E | 98.6 | Long |
| 12 | SPAG6 | testis | E | 43.0 | Mid |
| 13 | ARMC3 | testis | E | 15.2 | Long |
| 14 | GPR158 | brain | E | 26.7 | Long |
| 15 | LINC00836 | brain | P | 1.3 | Mid |
| 16 | MYO3A | testis | E | 3.0 | Long |
| 17 | GAD2 | brain | P | 13.9 | Mid |
| 18 | APBB1IP | spleen | E | 35.7 | Long |
| 19 | PTCHD3 | testis | E | 1.8 | Mid |
| 20 | C10orf126 | testis | Low | 0.4 | Mid |
| 21 | LYZL1 | testis | P | 38.5 | Mid |
| 22 | LYZL2 | testis | P | 65.0 | Mid |
| 23 | ANKRD30A | breast | P | 2.0 | Long |
| 25 | RET | brain | E | 4.0 | Mid |
| 26 | RASGEF1A | brain | E | 8.0 | Mid |
| 28 | ALOX5 | sm intestine | E | 19.4 | Mid |
| 30 | PTPN20A | testis | E | 0.4 | Mid |
| 33 | AGAP9 | testis | E | 15.8 | Mid |
| 35 | ANXA8 | skin | E | 96.8 | Mid |
| 37 | FRMPD2 | testis | E | 1.0 | Long |
| 39 | LRRC18 | testis | P | 19.4 | Short |
| 40 | DRGX | brain | Low | 1.0 | Mid |
| 41 | CHAT | brain | Low | 0.5 | Mid |
| 42 | C10orf53 | testis | P | 3.0 | Mid |
| 43 | OGDHL | kidney | E | 53.3 | Mid |
| 44 | ASAH2 | sm intestine | Low | 0.1 | Mid |
| 45 | A1CF | liver | E | 19.7 | Mid |
| 46 | PCDH15 | brain | E | 1.4 | Long |
| 47 | SLC16A9 | adrenal | E | 74.5 | Mid |
| 48 | CTNNA3 | brain | E | 13.1 | Long |
| 49 | STOX1 | brain | E | 12.0 | Mid |
| 50 | HKDC1 | sm intestine | E | 24.0 | Mid |
| 52 | NPFFR1 | brain | E | 4.6 | Mid |
| 54 | TBATA | testis | P | 22.2 | Short |
| 55 | C10orf11 | testis | E | 3.3 | Long |
| 56 | MAT1A | liver | E | 455.1 | Mid |
| 57 | DYDC1 | testis | P | 57.5 | Mid |
| 58 | SH2D4B | testis | Low | 0.3 | Long |
| 59 | NRG3 | brain | P | 6.5 | Long |
| 60 | CDHR1 | skin | E | 108.7 | Mid |
| 61 | RGR | brain | P | 2.6 | Short |
| 62 | GRID1 | brain | E | 6.8 | Long |
| 63 | LIPK | skin | P | 6.8 | Mid |
| 64 | FFAR4 | pituitary | E | 8.4 | Mid |
| 65 | LGI1 | brain | E | 24.3 | Mid |
| 66 | CYP2C18 | liver | E | 38.3 | Mid |
| 67 | CYP2C19 | liver | E | 7.7 | Mid |
| 68 | DNTT | testis | Low | 0.3 | Mid |
| 69 | TLL2 | brain | E | 1.5 | Long |
| 70 | PIK3AP1 | spleen | E | 23.7 | Long |
| 71 | CRTAC1 | artery | E | 19.4 | Long |
| 72 | HPSE2 | esophagus | E | 13.1 | Long |
| 73 | PAX2 | kidney | P | 36.9 | Mid |
| 74 | TLX1NB | spleen | E | 1.5 | Mid |
| 75 | SORCS3 | brain | E | 4.8 | Long |
| 76 | SORCS1 | nerve | E | 22.2 | Long |
| 78 | HABP2 | liver | E | 29.9 | Mid |
| 79 | NRAP | muscle | E | 584.5 | Mid |
| 80 | PLEKHS1 | salivary gland | E | 16.6 | Mid |
| 81 | TDRD1 | testis | P | 6.5 | Mid |
| 82 | VWA2 | stomach | E | 4.6 | Mid |
| 83 | AFAP1L2 | thyroid | E | 49.9 | Long |
| 84 | ATRNL1 | brain | E | 12.3 | Long |
| 85 | CCDC172 | testis | P | 11.8 | Mid |
| 86 | PNLIPRP3 | skin | P | 9.2 | Mid |
| 87 | PNLIP | pancreas | P | 20,091.1 | Short |
| 88 | KCNK18 | brain | Low | 0.1 | Short |
| 89 | EMX2OS | vagina | E | 15.2 | Mid |
| 90 | CASC2 | testis | E | 2.2 | Long |
| 91 | PPAPDC1A | brain | E | 5.6 | Long |
| 92 | FGFR2 | brain | E | 50.2 | Long |
| 93 | DMBT1 | sm intestine | E | 52.2 | Mid |
| 95 | GPR26 | brain | P | 1.2 | Mid |
| 96 | CPXM2 | artery | E | 101.2 | Long |
| 98 | TEX36 | testis | P | 13.5 | Long |
| 99 | C10orf90 | brain | E | 4.7 | Mid |
| 101 | TCERG1L | thyroid | E | 9.7 | Long |
| 102 | GPR123 | brain | P | 12.1 | Mid |

Chr11: All database tissue targeted genes on chromosome 11

| Index | Gene | Tissue | TSE/TSP | Exp | Length |
| --- | --- | --- | --- | --- | --- |
| 1 | LINC01001 | testis | E | 19.0 | Short |
| 2 | NLRP6 | sm intestine | E | 40.9 | Short |
| 3 | MUC2 | colon | E | 65.6 | Short |
| 4 | MUC5B | salivary gland | P | 301.4 | Mid |
| 5 | SYT8 | skin | E | 40.3 | Short |
| 6 | LSP1 | spleen | E | 71.0 | Mid |
| 7 | TNNT3 | muscle | P | 943.0 | Mid |
| 8 | TH | brain | P | 34.9 | Short |
| 9 | ASCL2 | skin | E | 4.8 | Short |
| 10 | TRPM5 | sm intestine | E | 3.6 | Mid |
| 12 | ART5 | testis | E | 22.4 | Short |
| 13 | C11orf40 | testis | Low | 0.1 | Short |
| 14 | OR51E1 | artery | E | 1.6 | Short |
| 15 | OR51E2 | prostate | E | 7.3 | Short |
| 16 | MMP26 | endometrium | P | 7.9 | Long |
| 17 | CNGA4 | testis | E | 6.4 | Short |
| 18 | CCKBR | stomach | E | 21.4 | Short |
| 19 | SYT9 | brain | E | 8.8 | Long |
| 20 | LMO1 | skin | E | 3.5 | Mid |
| 21 | ABCC8 | brain | E | 43.5 | Mid |
| 22 | USH1C | sm intestine | E | 29.1 | Mid |
| 23 | OTOG | testis | E | 1.5 | Mid |
| 24 | KCNC1 | brain | E | 41.3 | Mid |
| 25 | LDHC | testis | P | 166.4 | Mid |
| 26 | SLC6A5 | testis | Low | 0.4 | Mid |
| 27 | NELL1 | brain | E | 4.7 | Long |
| 28 | ANO5 | muscle | E | 12.7 | Mid |
| 29 | SLC17A6 | brain | E | 10.5 | Mid |
| 30 | GAS2 | liver | E | 5.0 | Long |
| 31 | BBOX1 | kidney | E | 39.0 | Mid |
| 33 | DCDC1 | testis | E | 1.3 | Long |
| 34 | WT1 | uterus | E | 35.6 | Mid |
| 35 | CCDC73 | testis | P | 1.6 | Long |
| 36 | PRRG4 | esophagus | E | 16.2 | Mid |
| 38 | ALX4 | breast | Low | 1.2 | Mid |
| 39 | SYT13 | brain | E | 35.1 | Mid |
| 40 | FOLH1 | brain | E | 25.5 | Mid |
| 41 | P2RX3 | testis | E | 5.1 | Mid |
| 42 | GLYAT | liver | E | 53.2 | Mid |
| 43 | GIF | stomach | P | 838.8 | Short |
| 44 | MS4A4A | spleen | E | 30.1 | Mid |
| 45 | LINC00301 | testis | P | 58.9 | Mid |
| 46 | CD6 | spleen | E | 13.6 | Mid |
| 47 | PGA3 | stomach | P | 2004.3 | Short |
| 48 | SYT7 | brain | E | 40.6 | Mid |
| 49 | ASRGL1 | testis | E | 50.2 | Mid |
| 50 | SLC22A6 | kidney | E | 43.7 | Short |
| 51 | SLC22A24 | kidney | Low | 0.2 | Mid |
| 52 | SLC22A25 | liver | P | 8.6 | Mid |
| 53 | GAL | pituitary | P | 309.7 | Short |
| 54 | FGF19 | testis | Low | 0.2 | Short |
| 57 | MOGAT2 | sm intestine | E | 35.6 | Short |
| 58 | WNT11 | adipose | E | 17.1 | Mid |
| 59 | MYO7A | adrenal | E | 21.3 | Mid |
| 60 | CCDC83 | testis | P | 19.4 | Mid |
| 61 | GRM5 | brain | P | 8.3 | Long |
| 63 | NAALAD2 | pituitary | E | 15.9 | Mid |
| 64 | DISC1FP1 | spleen | Low | 0.1 | Long |
| 65 | CCDC67 | testis | P | 11.0 | Mid |
| 66 | GPR83 | brain | E | 9.3 | Mid |
| 67 | CNTN5 | brain | E | 1.2 | Long |
| 68 | PGR | cervix | E | 30.5 | Long |
| 69 | MMP20 | testis | P | 1.2 | Mid |
| 70 | GUCY1A2 | uterus | E | 5.5 | Long |
| 71 | ELMOD1 | brain | E | 21.9 | Mid |
| 72 | RAB39A | brain | E | 0.8 | Mid |
| 73 | C11orf53 | testis | E | 5.3 | Mid |
| 74 | BTG4 | testis | P | 12.6 | Mid |
| 75 | NCAM1 | brain | E | 36.3 | Long |
| 76 | DRD2 | pituitary | E | 43.0 | Mid |
| 77 | HTR3B | brain | P | 1.0 | Mid |
| 78 | HTR3A | brain | E | 1.4 | Mid |
| 80 | NXPE2 | colon | E | 2.4 | Mid |
| 81 | DSCAML1 | brain | E | 5.5 | Long |
| 82 | FXYD6 | brain | E | 212.1 | Mid |
| 83 | TMPRSS13 | skin | E | 16.8 | Mid |
| 84 | IL10RA | spleen | E | 74.7 | Short |
| 85 | TRIM29 | skin | E | 317.3 | Short |
| 86 | BARX2 | esophagus | E | 52.2 | Mid |
| 87 | ST14 | colon | E | 123.0 | Mid |
| 88 | OPCML | brain | P | 32.3 | Long |
| 89 | GLB1L3 | brain | E | 3.6 | Mid |
| 90 | GLB1L2 | prostate | E | 11.3 | Mid |
| 91 | B3GAT1 | brain | E | 49.6 | Mid |

Chr12: All database tissue targeted genes on chromosome 12

| Index | Gene | Tissue | TSE/TSP | Exp | Length |
| --- | --- | --- | --- | --- | --- |
| 1 | SLC6A12 | kidney | E | 18.7 | Mid |
| 2 | SLC6A13 | kidney | E | 18.5 | Mid |
| 3 | CACNA2D4 | testis | E | 3.4 | Long |
| 4 | PRMT8 | brain | E | 12.3 | Long |
| 5 | FGF6 | muscle | Low | 0.8 | Short |
| 6 | KCNA6 | brain | P | 7.0 | Short |
| 7 | ANO2 | testis | E | 2.6 | Long |
| 8 | APOBEC1 | sm intestine | P | 15.1 | Mid |
| 9 | LINC00937 | spleen | Low | 1.0 | Short |
| 10 | CLEC6A | lung | Low | 0.1 | Mid |
| 11 | PZP | liver | E | 4.7 | Mid |
| 12 | CD69 | spleen | E | 22.2 | Short |
| 13 | KLRF2 | skin | Low | 0.9 | Short |
| 14 | CLEC1A | adipose | E | 6.4 | Mid |
| 15 | CLEC7A | spleen | E | 24.5 | Short |
| 16 | STYK1 | colon | E | 5.7 | Mid |
| 17 | GRIN2B | brain | P | 4.0 | Long |
| 18 | GUCY2C | sm intestine | E | 22.5 | Mid |
| 20 | PTPRO | kidney | E | 6.6 | Long |
| 21 | SLC15A5 | testis | Low | 0.1 | Mid |
| 22 | RERGL | artery | E | 48.3 | Short |
| 23 | PIK3C2G | stomach | E | 10.0 | Long |
| 24 | PLCZ1 | testis | P | 29.5 | Mid |
| 25 | SLCO1C1 | brain | P | 10.0 | Mid |
| 26 | SLCO1B3 | liver | P | 32.6 | Long |
| 27 | GYS2 | liver | E | 20.8 | Mid |
| 28 | ST8SIA1 | brain | E | 3.4 | Long |
| 29 | LRMP | sm intestine | E | 5.6 | Mid |
| 30 | CASC1 | testis | E | 16.2 | Mid |
| 31 | TSPAN11 | cervix | E | 2.0 | Mid |
| 32 | PKP2 | heart | E | 60.4 | Long |
| 33 | SYT10 | nerve | E | 1.0 | Mid |
| 35 | ABCD2 | brain | E | 4.3 | Mid |
| 36 | C12orf40 | testis | P | 2.1 | Mid |
| 37 | MUC19 | salivary gland | E | 4.7 | Short |
| 38 | CNTN1 | brain | E | 53.1 | Long |
| 39 | PDZRN4 | colon | E | 16.5 | Long |
| 40 | ADAMTS20 | adipose | E | 20.4 | Long |
| 41 | NELL2 | brain | E | 35.0 | Long |
| 42 | DBX2 | testis | Low | 0.3 | Mid |
| 43 | TMPRSS12 | testis | P | 8.8 | Mid |
| 44 | NCKAP1L | spleen | E | 34.4 | Mid |
| 45 | PDE1B | brain | E | 131.6 | Mid |
| 46 | TESPA1 | brain | E | 42.8 | Mid |
| 47 | SDR9C7 | skin | E | 39.5 | Short |
| 48 | LINC02389 | brain | P | 0.6 | Long |
| 49 | WIF1 | brain | E | 53.3 | Mid |
| 50 | GRIP1 | pituitary | E | 2.6 | Long |
| 51 | IL26 | spleen | Low | 0.1 | Mid |
| 52 | TSPAN8 | colon | E | 282.0 | Long |
| 53 | LGR5 | muscle | E | 6.9 | Long |
| 54 | KCNC2 | brain | E | 21.9 | Long |
| 55 | OTOGL | heart | E | 2.4 | Long |
| 56 | PPFIA2 | brain | E | 13.8 | Long |
| 57 | ALX1 | fallopian tube | E | 2.1 | Mid |
| 58 | RASSF9 | esophagus | E | 2.8 | Mid |
| 59 | MGAT4C | testis | E | 2.5 | Long |
| 60 | C12orf50 | testis | P | 44.3 | Mid |
| 61 | LINC00615 | testis | P | 0.6 | Mid |
| 62 | CCDC38 | testis | P | 7.6 | Mid |
| 63 | AMDHD1 | liver | E | 46.0 | Mid |
| 64 | RMST | thyroid | E | 37.2 | Long |
| 65 | ANKS1B | brain | P | 13.6 | Long |
| 66 | SLC17A8 | sm intestine | P | 3.5 | Mid |
| 67 | NR1H4 | liver | E | 24.3 | Mid |
| 68 | SLC5A8 | thyroid | P | 10.5 | Mid |
| 69 | SPIC | spleen | P | 12.9 | Short |
| 70 | MYBPC1 | muscle | P | 1743.7 | Mid |
| 71 | IGF1 | cervix | E | 14.3 | Mid |
| 72 | LINC00485 | testis | Low | 0.2 | Mid |
| 73 | PAH | liver | E | 173.2 | Short |
| 74 | C12orf42 | testis | E | 8.4 | Long |
| 75 | RFX4 | testis | E | 43.9 | Long |
| 76 | WSCD2 | brain | E | 56.3 | Long |
| 77 | CMKLR1 | spleen | E | 21.0 | Mid |
| 78 | DAO | brain | E | 16.4 | Mid |
| 79 | SVOP | brain | E | 31.7 | Long |
| 80 | FOXN4 | testis | P | 10.6 | Mid |
| 81 | FAM222A | testis | E | 21.5 | Short |
| 82 | CCDC63 | testis | P | 18.5 | Mid |
| 83 | CUX2 | brain | E | 9.4 | Long |
| 84 | RPH3A | brain | P | 32.9 | Long |
| 85 | OAS1 | spleen | E | 15.7 | Short |
| 86 | OAS3 | esophagus | E | 7.7 | Mid |
| 87 | DTX1 | spleen | E | 22.1 | Mid |
| 88 | LHX5 | brain | E | 1.7 | Short |
| 89 | LINC01234 | brain | E | 0.8 | Long |
| 91 | NOS1 | muscle | E | 3.7 | Long |
| 92 | KSR2 | pituitary | E | 9.4 | Long |
| 93 | SRRM4 | brain | E | 35.6 | Long |
| 94 | LINC00934 | testis | Low | 0.2 | Mid |
| 95 | CCDC60 | testis | E | 7.2 | Long |
| 96 | TMEM233 | muscle | E | 17.7 | Mid |
| 97 | CCDC64 | pituitary | E | 54.7 | Long |
| 98 | MSI1 | testis | E | 16.1 | Mid |
| 99 | CABP1 | brain | P | 103.1 | Short |
| 100 | OASL | lung | E | 4.2 | Mid |
| 101 | LRRC43 | testis | E | 6.8 | Mid |
| 102 | TMEM132B | brain | E | 8.4 | Long |
| 103 | LINC00939 | testis | E | 1.4 | Mid |
| 104 | LINC00944 | testis | E | 3.7 | Short |
| 105 | TMEM132C | cervix | E | 17.5 | Long |
| 106 | GLT1D1 | testis | E | 16.3 | Long |
| 107 | TMEM132D | brain | P | 4.6 | Short |
| 108 | PIWIL1 | testis | P | 39.0 | Short |
| 109 | RIMBP2 | pituitary | E | 27.6 | Long |
| 110 | ADGRD1 | heart | E | 21.1 | Long |
| 111 | GALNT9 | brain | E | 68.3 | Long |

Chr13: All database tissue targeted genes on chromosome 13

| Index | Gene | Tissue | TSE/TSP | Exp | Length |
| --- | --- | --- | --- | --- | --- |
| 2 | LINC00408 | testis | P | 2.6 | Mid |
| 3 | TUBA3C | testis | P | 438.6 | Short |
| 5 | TPTE2 | testis | P | 7.6 | Long |
| 6 | GJB6 | esophagus | E | 101.6 | Short |
| 7 | LINC00540 | placenta | E | 0.8 | Mid |
| 8 | SGCG | heart | E | 28.7 | Long |
| 11 | ATP12A | skin | Low | 1.5 | Mid |
| 12 | RNF17 | testis | P | 15.9 | Long |
| 14 | ATP8A2 | brain | E | 17.5 | Long |
| 15 | FLT3 | brain | E | 8.8 | Mid |
| 16 | MTUS2 | heart | E | 6.6 | Mid |
| 17 | LINC00297 | adipose | Low | 0.1 | Mid |
| 18 | LINC00544 | adipose | Low | 0.1 | Short |
| 20 | LINC00423 | testis | Low | 0.1 | Mid |
| 21 | KL | kidney | E | 7.3 | Mid |
| 22 | LINC00457 | testis | Low | 0.1 | Long |
| 23 | DCLK1 | brain | E | 22.9 | Long |
| 24 | CCNA1 | testis | E | 57.4 | Short |
| 25 | SERTM1 | fallopian tube | E | 23.1 | Mid |
| 26 | LINC00571 | thyroid | E | 0.3 | Long |
| 27 | FREM2 | thyroid | E | 2.7 | Long |
| 29 | LINC00332 | testis | Low | 0.3 | Mid |
| 30 | LINC00598 | adipose | E | 0.3 | Long |
| 31 | TNFSF11 | testis | P | 0.8 | Mid |
| 32 | SPERT | testis | P | 77.4 | Short |
| 33 | SIAH3 | brain | E | 1.0 | Mid |
| 34 | LRRC63 | brain | E | 3.5 | Mid |
| 35 | DLEU7 | brain | E | 1.7 | Mid |
| 37 | FAM124A | brain | E | 12.5 | Mid |
| 38 | TMEM272 | lymph node | E | 0.6 | Mid |
| 39 | NEK5 | testis | P | 5.9 | Mid |
| 41 | OLFM4 | sm intestine | E | 602.1 | Mid |
| 42 | LINC00558 | testis | P | 0.5 | Long |
| 43 | PCDH17 | spleen | E | 13.4 | Mid |
| 44 | LINC00358 | testis | P | 2.5 | Mid |
| 45 | LINC00395 | testis | P | 2.2 | Mid |
| 46 | KLHL1 | brain | P | 4.8 | Long |
| 47 | LINC00348 | testis | P | 1.5 | Long |
| 49 | SCEL | esophagus | E | 186.4 | Long |
| 50 | SLAIN1 | brain | E | 94.9 | Mid |
| 51 | LINC00331 | testis | P | 1.5 | Mid |
| 52 | LINC00351 | testis | P | 0.4 | Long |
| 53 | LINC00379 | kidney | P | 1.0 | Short |
| 54 | DCT | skin | E | 11.1 | Mid |
| 55 | HS6ST3 | artery | E | 1.4 | Long |
| 56 | LINC00359 | testis | E | 1.6 | Mid |
| 57 | SLC15A1 | skin | E | 12.8 | Mid |
| 58 | FGF14 | brain | E | 4.5 | Long |
| 59 | SLC10A2 | sm intestine | P | 54.9 | Mid |
| 60 | LINC00343 | testis | P | 5.8 | Mid |
| 61 | LINC00551 | esophagus | E | 1.3 | Long |
| 62 | SPACA7 | testis | P | 60.3 | Mid |
| 63 | ATP11AUN | testis | Low | 0.2 | Mid |
| 64 | MCF2L | brain | E | 45.1 | Long |

Chr14: All database tissue targeted genes on chromosome 14

| Index | Gene | Tissue | TSE/TSP | Exp | Length |
| --- | --- | --- | --- | --- | --- |
| 1 | POTEG | testis | Low | 1.1 | Mid |
| 3 | POTEM | prostate | E | 10.9 | Mid |
| 4 | RPGRIP1 | testis | P | 17.6 | Mid |
| 6 | MYH6 | heart | E | 3252.3 | Mid |
| 7 | MYH7 | heart | E | 3854.0 | Mid |
| 8 | LINC00645 | kidney | E | 1.9 | Mid |
| 9 | NPAS3 | brain | P | 5.6 | Long |
| 10 | LINC00609 | brain | P | 2.6 | Mid |
| 11 | PTCSC3 | thyroid | P | 31.0 | Mid |
| 12 | SFTA3 | thyroid | E | 77.5 | Mid |
| 13 | LINC00639 | brain | E | 3.3 | Long |
| 14 | MIA2 | liver | E | 1.1 | Mid |
| 15 | LINC00871 | kidney | Low | 0.2 | Long |
| 16 | MDGA2 | testis | P | 0.4 | Long |
| 17 | LINC00640 | skin | E | 2.5 | Mid |
| 18 | C14orf105 | kidney | E | 33.6 | Mid |
| 19 | SLC35F4 | brain | P | 4.7 | Long |
| 20 | CCDC175 | brain | E | 6.6 | Mid |
| 21 | LRRC9 | testis | P | 4.4 | Long |
| 23 | KCNH5 | brain | E | 0.5 | Long |
| 24 | SPTB | muscle | E | 47.2 | Long |
| 25 | TMEM229B | brain | E | 15.3 | Mid |
| 26 | GALNT16 | brain | E | 12.6 | Mid |
| 27 | PLEKHD1 | brain | E | 5.5 | Mid |
| 28 | SLC8A3 | muscle | E | 4.2 | Long |
| 29 | RGS6 | brain | E | 4.4 | Long |
| 30 | VSX2 | testis | E | 0.3 | Mid |
| 31 | SYNDIG1L | brain | P | 271.0 | Mid |
| 32 | BATF | spleen | E | 4.4 | Mid |
| 33 | ESRRB | kidney | E | 2.5 | Long |
| 34 | LRRC74 | testis | P | 13.4 | Mid |
| 35 | TMEM63C | pituitary | E | 39.5 | Mid |
| 36 | TSHR | thyroid | P | 84.4 | Long |
| 37 | STON2 | brain | E | 13.4 | Long |
| 38 | LINC00911 | testis | P | 4.0 | Mid |
| 39 | LINC01146 | liver | E | 5.3 | Mid |
| 40 | KCNK10 | brain | E | 2.3 | Mid |
| 41 | KCNK13 | testis | E | 9.8 | Long |
| 42 | CATSPERB | pancreas | E | 3.2 | Long |
| 43 | SLC24A4 | nerve | E | 2.3 | Long |
| 44 | UNC79 | pituitary | E | 13.0 | Long |
| 45 | FAM181A-AS1 | testis | P | 9.2 | Short |
| 46 | PPP4R4 | brain | E | 7.8 | Long |
| 48 | TUNAR | brain | E | 28.1 | Mid |
| 49 | AK7 | testis | E | 3.5 | Mid |
| 50 | C14orf64 | skin | E | 1.3 | Mid |
| 51 | BCL11B | skin | E | 9.4 | Long |
| 52 | TDRD9 | testis | E | 34.5 | Long |
| 53 | ASPG | liver | E | 59.8 | Short |

Chr15: All database tissue targeted genes on chromosome 15

| Index | Gene | Tissue | TSE/TSP | Exp | Length |
| --- | --- | --- | --- | --- | --- |
| 2 | GOLGA6L6 | testis | P | 5.0 | Short |
| 4 | LINC01193 | testis | Low | 1.5 | Mid |
| 5 | GOLGA6L1 | testis | P | 6.6 | Short |
| 6 | PWRN1 | testis | E | 12.2 | Long |
| 8 | LINC00929 | testis | P | 6.8 | Mid |
| 9 | GABRB3 | brain | E | 16.2 | Long |
| 10 | GABRG3 | testis | E | 2.1 | Long |
| 11 | OCA2 | thyroid | E | 3.6 | Long |
| 12 | GOLGA8G | testis | P | 3.1 | Short |
| 14 | CHRFAM7A | testis | Low | 0.9 | Mid |
| 15 | TRPM1 | skin | E | 1.4 | Mid |
| 16 | OTUD7A | testis | E | 2.8 | Long |
| 17 | CHRNA7 | testis | E | 2.1 | Long |
| 18 | RYR3 | brain | E | 5.9 | Long |
| 19 | TMCO5A | testis | P | 31.1 | Mid |
| 20 | EXD1 | testis | P | 18.1 | Mid |
| 21 | PLA2G4E | skin | E | 28.1 | Mid |
| 22 | PLA2G4D | skin | E | 13.5 | Mid |
| 23 | TGM5 | skin | E | 29.5 | Mid |
| 24 | TGM7 | testis | P | 2.0 | Mid |
| 26 | C15orf43 | testis | P | 12.5 | Mid |
| 27 | GATM | pancreas | E | 355.4 | Mid |
| 28 | SLC12A1 | kidney | P | 36.1 | Long |
| 29 | CYP19A1 | nerve | Low | 0.1 | Long |
| 30 | GLDN | nerve | E | 22.1 | Mid |
| 31 | ONECUT1 | pancreas | E | 2.6 | Mid |
| 32 | WDR72 | thyroid | E | 15.0 | Long |
| 33 | UNC13C | brain | P | 25.9 | Long |
| 34 | CGNL1 | kidney | E | 24.3 | Long |
| 35 | MYZAP | heart | Low | 0.8 | Mid |
| 36 | ALDH1A2 | testis | E | 17.7 | Long |
| 37 | FAM81A | brain | E | 15.7 | Mid |
| 38 | DAPK2 | thyroid | E | 21.8 | Long |
| 39 | GOLGA6B | testis | P | 3.1 | Short |
| 40 | CCDC33 | testis | P | 11.8 | Mid |
| 41 | GOLGA6C | testis | P | 4.2 | Short |
| 42 | NRG4 | brain | E | 11.4 | Mid |
| 43 | CRABP2 | esophagus | E | 481.0 | Short |
| 44 | CHRNB4 | testis | P | 4.6 | Mid |
| 45 | BCL2A1 | spleen | E | 33.2 | Short |
| 46 | LINC00927 | testis | P | 6.9 | Mid |
| 50 | HOMER2 | pancreas | E | 22.0 | Long |
| 51 | SH3GL3 | testis | E | 46.6 | Long |
| 52 | ADAMTSL3 | artery | E | 12.2 | Long |
| 54 | AGBL1 | muscle | E | 0.7 | Long |
| 55 | NTRK3 | artery | E | 20.6 | Long |
| 56 | ACAN | artery | E | 11.9 | Mid |
| 57 | RHCG | esophagus | E | 1989.3 | Mid |
| 58 | LINC00928 | testis | P | 1.6 | Mid |
| 59 | WDR93 | testis | E | 7.3 | Mid |
| 60 | SV2B | brain | P | 29.6 | Long |
| 61 | ST8SIA2 | brain | E | 0.7 | Mid |
| 64 | LINC01197 | ovary | E | 5.7 | Long |
| 65 | LINC00924 | ovary | E | 5.4 | Mid |
| 66 | FAM169B | testis | E | 0.9 | Mid |
| 67 | PGPEP1L | muscle | E | 4.5 | Mid |
| 68 | CERS3 | skin | E | 27.9 | Long |
| 69 | PCSK6 | liver | E | 64.1 | Long |

Chr16: All database tissue targeted genes on chromosome 16

| Index | Gene | Tissue | TSE/TSP | Exp | Length |
| --- | --- | --- | --- | --- | --- |
| 2 | SSTR5-AS1 | adrenal | E | 1.5 | Short |
| 3 | IL32 | spleen | E | 110.0 | Short |
| 5 | C16orf96 | testis | P | 13.2 | Mid |
| 6 | SEPT12 | testis | P | 98.8 | Short |
| 7 | PPL | esophagus | E | 470.4 | Mid |
| 8 | C16orf89 | thyroid | E | 94.2 | Mid |
| 9 | RBFOX1 | brain | E | 30.7 | Long |
| 10 | GRIN2A | brain | E | 4.9 | Long |
| 11 | TEKT5 | testis | P | 29.0 | Mid |
| 12 | TVP23A | ovary | E | 3.0 | Mid |
| 13 | CIITA | spleen | E | 15.7 | Mid |
| 14 | BCAR4 | placenta | P | 8.2 | Short |
| 15 | SHISA9 | brain | E | 3.2 | Long |
| 16 | PLA2G10 | colon | E | 10.2 | Mid |
| 17 | NPIPA2 | testis | E | 2.2 | Mid |
| 19 | NOMO3 | pancreas | E | 7.1 | Mid |
| 22 | SYT17 | brain | E | 15.1 | Long |
| 23 | CLEC19A | prostate | E | 0.1 | Mid |
| 24 | TMC5 | sm intestine | E | 19.9 | Mid |
| 25 | GPR139 | brain | Low | 0.2 | Mid |
| 26 | GP2 | pancreas | P | 9286.7 | Short |
| 27 | UMOD | kidney | P | 271.1 | Mid |
| 28 | PDILT | stomach | E | 54.7 | Mid |
| 29 | ACSM5 | liver | E | 64.4 | Mid |
| 30 | ZP2 | brain | P | 26.3 | Short |
| 31 | ANKS4B | sm intestine | E | 12.7 | Mid |
| 32 | OTOA | testis | E | 3.9 | Mid |
| 33 | HS3ST2 | brain | E | 9.3 | Long |
| 34 | SCNN1G | kidney | E | 17.0 | Mid |
| 35 | SCNN1B | esophagus | E | 37.5 | Mid |
| 36 | PRKCB | brain | E | 48.1 | Long |
| 37 | CACNG3 | brain | P | 31.8 | Long |
| 38 | SLC5A11 | brain | P | 35.2 | Mid |
| 39 | AQP8 | pancreas | E | 184.4 | Short |
| 40 | HS3ST4 | brain | P | 11.8 | Long |
| 41 | GSG1L | brain | E | 7.2 | Long |
| 42 | SBK1 | brain | E | 34.1 | Mid |
| 44 | APOBR | spleen | E | 53.2 | Short |
| 45 | ITGAL | spleen | E | 49.6 | Mid |
| 46 | TRIM72 | muscle | E | 31.6 | Short |
| 47 | ITGAM | spleen | E | 22.8 | Mid |
| 48 | ITGAX | spleen | E | 71.3 | Mid |
| 49 | ITGAD | spleen | P | 10.1 | Mid |
| 52 | ABCC12 | testis | P | 6.0 | Mid |
| 53 | ABCC11 | testis | P | 2.5 | Mid |
| 54 | C16orf78 | testis | P | 46.1 | Mid |
| 55 | ZNF423 | brain | E | 3.4 | Long |
| 56 | NKD1 | artery | E | 7.7 | Long |
| 57 | SNX20 | spleen | E | 9.3 | Short |
| 58 | NOD2 | skin | E | 8.2 | Mid |
| 59 | C16orf97 | testis | P | 5.0 | Mid |
| 60 | LINC00919 | testis | P | 43.9 | Short |
| 61 | CASC16 | testis | P | 15.5 | Mid |
| 62 | SLC8A2 | brain | E | 86.5 | Mid |
| 65 | CES1 | liver | E | 223.0 | Mid |
| 66 | CES5A | liver | E | 1.3 | Mid |
| 67 | GNAO1 | brain | E | 52.8 | Long |
| 68 | SLC12A3 | kidney | P | 32.2 | Mid |
| 69 | PLLP | nerve | E | 124.4 | Mid |
| 70 | CCL17 | lung | E | 1.5 | Short |
| 71 | ADGRG5 | spleen | E | 5.9 | Mid |
| 72 | ADGRG3 | spleen | E | 34.2 | Mid |
| 73 | DRC7 | testis | P | 33.5 | Mid |
| 74 | CNGB1 | brain | E | 2.1 | Mid |
| 75 | TEPP | testis | E | 14.3 | Short |
| 76 | MMP15 | thyroid | E | 36.0 | Mid |
| 77 | CDH8 | brain | E | 3.4 | Long |
| 78 | LINC00922 | lung | Low | 0.5 | Long |
| 80 | LRRC36 | testis | E | 29.8 | Mid |
| 81 | SMPD3 | sm intestine | E | 10.6 | Mid |
| 82 | CDH3 | ovary | E | 26.5 | Mid |
| 83 | CDH1 | esophagus | E | 61.0 | Mid |
| 84 | CLEC18C | kidney | E | 3.3 | Short |
| 85 | IL34 | spleen | E | 73.0 | Mid |
| 86 | CALB2 | brain | E | 172.7 | Mid |
| 87 | CHST4 | pancreas | E | 1.8 | Short |
| 88 | PMFBP1 | testis | P | 13.9 | Mid |
| 89 | C16orf47 | bladder | E | 5.9 | Mid |
| 90 | CLEC18B | kidney | E | 5.5 | Short |
| 91 | FA2H | brain | E | 88.9 | Mid |
| 92 | CHST6 | adrenal | E | 3.4 | Mid |
| 93 | CNTNAP4 | brain | P | 60.0 | Long |
| 94 | ADAMTS18 | brain | P | 11.9 | Long |
| 95 | CLEC3A | bladder | Low | 1.0 | Short |
| 96 | PLCG2 | spleen | E | 24.5 | Long |
| 97 | NECAB2 | brain | E | 65.1 | Mid |
| 98 | SLC38A8 | brain | E | 0.9 | Mid |
| 99 | DNAAF1 | testis | E | 46.6 | Mid |
| 100 | FAM92B | pituitary | E | 10.8 | Short |
| 101 | IRF8 | spleen | E | 56.0 | Mid |
| 102 | CA5A | liver | P | 15.5 | Mid |
| 104 | DPEP1 | sm intestine | E | 266.0 | Mid |
| 105 | PRDM7 | testis | P | 2.1 | Mid |

Chr17: All database tissue targeted genes on chromosome 17

| Index | Gene | Tissue | TSE/TSP | Exp | Length |
| --- | --- | --- | --- | --- | --- |
| 1 | TUSC5 | adipose | E | 65.6 | Mid |
| 2 | SERPINF2 | liver | E | 669.0 | Low |
| 3 | SPATA22 | testis | P | 78.1 | Mid |
| 4 | ASPA | brain | E | 13.7 | Mid |
| 5 | SPNS3 | spleen | E | 2.1 | Mid |
| 6 | SMTNL2 | muscle | E | 135.9 | Mid |
| 7 | ALOX15 | adipose | E | 8.9 | Low |
| 8 | TM4SF5 | liver | E | 66.9 | Low |
| 9 | USP6 | testis | E | 14.2 | Mid |
| 10 | WSCD1 | brain | E | 12.5 | Mid |
| 11 | AIPL1 | testis | E | 0.3 | Low |
| 12 | SLC13A5 | liver | P | 163.4 | Low |
| 13 | FBXO39 | testis | P | 19.8 | Low |
| 14 | TEKT1 | testis | P | 16.0 | Mid |
| 16 | CLEC10A | sm intestine | E | 5.7 | Low |
| 17 | ASGR2 | liver | P | 232.2 | Low |
| 18 | SLC2A4 | muscle | E | 53.3 | Low |
| 19 | GUCY2D | testis | P | 1.8 | Mid |
| 20 | ALOX15B | prostate | E | 36.4 | Low |
| 21 | ALOX12B | skin | P | 63.8 | Low |
| 22 | ARHGEF15 | adipose | E | 29.6 | Low |
| 23 | ODF4 | testis | P | 13.4 | Low |
| 24 | CCDC42 | testis | P | 67.9 | Low |
| 25 | SPDYE4 | testis | P | 6.1 | Low |
| 26 | PIK3R6 | spleen | E | 2.6 | Mid |
| 27 | PIK3R5 | spleen | E | 20.0 | Mid |
| 28 | NTN1 | esophagus | E | 41.5 | Long |
| 29 | CFAP52 | testis | E | 9.9 | Mid |
| 30 | USP43 | esophagus | E | 3.0 | Mid |
| 31 | DHRS7C | heart | E | 52.9 | Mid |
| 32 | GLP2R | colon | E | 6.5 | Mid |
| 33 | MYH13 | muscle | E | 1.1 | Mid |
| 34 | MYH8 | muscle | E | 1.9 | Mid |
| 35 | LINC00675 | colon | E | 15.6 | Low |
| 36 | PIRT | brain | E | 5.9 | Mid |
| 37 | SHISA6 | brain | E | 9.1 | Long |
| 38 | DNAH9 | testis | E | 2.2 | Long |
| 39 | TEKT3 | testis | P | 28.5 | Mid |
| 40 | TBC1D26 | testis | P | 1.3 | Low |
| 41 | CCDC144A | testis | E | 0.8 | Mid |
| 43 | TNFRSF13B | spleen | E | 13.3 | Mid |
| 45 | LGALS9C | colon | E | 4.8 | Mid |
| 46 | FBXW10 | testis | P | 7.4 | Mid |
| 47 | GRAP | spleen | E | 7.4 | Mid |
| 48 | GRAPL | testis | Low | 0.1 | Mid |
| 49 | SLC47A1 | adrenal | E | 59.3 | Mid |
| 50 | ALDH3A1 | esophagus | E | 155.6 | Low |
| 52 | LGALS9B | esophagus | E | 5.3 | Mid |
| 53 | CCDC144NL | testis | P | 1.6 | Mid |
| 55 | NOS2 | sm intestine | E | 2.6 | Mid |
| 56 | SLC13A2 | sm intestine | E | 52.1 | Mid |
| 57 | FOXN1 | skin | E | 26.3 | Mid |
| 58 | PIPOX | liver | E | 81.8 | Long |
| 59 | SLC6A4 | lung | E | 14.0 | Mid |
| 60 | TMIGD1 | sm intestine | E | 72.8 | Mid |
| 61 | RAB11FIP4 | brain | E | 25.4 | Long |
| 62 | RHBDL3 | brain | E | 6.8 | Mid |
| 63 | SPACA3 | testis | P | 81.1 | Low |
| 64 | ASIC2 | brain | E | 5.7 | Long |
| 65 | TMEM132E | brain | P | 13.2 | Mid |
| 66 | UNC45B | muscle | E | 40.5 | Mid |
| 67 | SLFN13 | salivary gland | E | 7.0 | Low |
| 68 | SLFN14 | spleen | E | 0.7 | Low |
| 69 | C17orf50 | testis | P | 13.1 | Low |
| 70 | MMP28 | adipose | E | 8.8 | Mid |
| 71 | LYZL6 | testis | P | 15.7 | Low |
| 72 | CCL23 | lymph node | E | 3.6 | Low |
| 73 | TBC1D3H | testis | E | 20.7 | Low |
| 74 | LHX1 | kidney | P | 4.6 | Low |
| 75 | HNF1B | kidney | P | 16.9 | Mid |
| 76 | SRCIN1 | brain | E | 9.1 | Mid |
| 78 | IKZF3 | spleen | E | 3.0 | Long |
| 79 | ZPBP2 | testis | P | 70.0 | Low |
| 80 | LRRC3C | testis | P | 0.6 | Low |
| 81 | GSDMA | skin | P | 36.5 | Mid |
| 83 | HAP1 | brain | E | 15.1 | Low |
| 84 | RAMP2-AS1 | adipose | E | 7.0 | Low |
| 85 | LINC00671 | kidney | E | 16.3 | Mid |
| 86 | G6PC | pancreas | E | 4.7 | Low |
| 87 | CD300LG | adipose | E | 41.1 | Mid |
| 88 | SLC4A1 | kidney | E | 6.1 | Mid |
| 90 | WNT3 | skin | E | 15.8 | Mid |
| 91 | WNT9B | kidney | Low | 0.3 | Mid |
| 92 | MYL4 | heart | E | 1639.1 | Low |
| 93 | TBX21 | spleen | E | 8.9 | Low |
| 94 | SKAP1 | pituitary | E | 9.5 | Long |
| 95 | HOXB9 | colon | E | 23.3 | Low |
| 96 | TTLL6 | testis | E | 21.5 | Mid |
| 97 | NGFR | nerve | E | 102.4 | Mid |
| 98 | DLX3 | skin | E | 33.7 | Low |
| 99 | CACNA1G | brain | E | 14.3 | Mid |
| 100 | CA10 | brain | P | 63.3 | Long |
| 102 | LPO | salivary gland | P | 26.5 | Mid |
| 103 | MPO | spleen | E | 7.7 | Low |
| 104 | RNF43 | sm intestine | E | 5.2 | Mid |
| 105 | HSF5 | testis | P | 30.1 | Mid |
| 106 | PPM1E | testis | E | 9.2 | Long |
| 107 | C17orf64 | testis | P | 110.0 | Low |
| 108 | TBX4 | lung | E | 19.3 | Mid |
| 110 | EFCAB3 | testis | P | 3.3 | Mid |
| 111 | KCNH6 | pituitary | E | 4.1 | Mid |
| 112 | SCN4A | muscle | E | 37.7 | Mid |
| 113 | PRR29 | spleen | E | 19.8 | Low |
| 114 | PECAM1 | placenta | E | 145.1 | Mid |
| 115 | MILR1 | lymph node | E | 6.4 | Mid |
| 116 | APOH | liver | P | 1914.6 | Low |
| 117 | CACNG5 | brain | E | 0.3 | Mid |
| 118 | CACNG4 | brain | P | 12.6 | Mid |
| 119 | ABCA6 | nerve | E | 20.8 | Mid |
| 120 | LINC01028 | nerve | E | 4.4 | Mid |
| 121 | KCNJ16 | thyroid | E | 51.8 | Mid |
| 122 | CASC17 | testis | P | 2.6 | Long |
| 123 | LINC00511 | bladder | E | 3.6 | Long |
| 124 | SDK2 | testis | E | 8.6 | Long |
| 125 | LINC00469 | low | Low | 0.2 | Mid |
| 126 | DNAI2 | testis | P | 26.1 | Mid |
| 127 | KIF19 | nerve | E | 51.2 | Mid |
| 128 | BTBD17 | brain | P | 2.5 | Low |
| 130 | CD300LB | lymph node | E | 4.4 | Low |
| 131 | RAB37 | brain | E | 54.8 | Mid |
| 132 | FADS6 | sm intestine | E | 7.9 | Mid |
| 133 | USH1G | esophagus | E | 4.6 | Low |
| 134 | OTOP3 | esophagus | E | 9.2 | Low |
| 135 | HID1 | brain | E | 122.3 | Mid |
| 136 | EVPL | esophagus | E | 142.9 | Mid |
| 138 | TMC8 | spleen | E | 88.3 | Low |
| 139 | TMEM235 | brain | P | 17.7 | Low |
| 140 | DNAH17 | testis | E | 9.1 | Long |
| 141 | RBFOX3 | brain | E | 11.3 | Long |
| 142 | ENPP7 | liver | E | 2.3 | Low |
| 143 | AATK | nerve | E | 161.4 | Mid |
| 144 | TMEM105 | liver | E | 0.7 | Mid |
| 145 | NOTUM | testis | E | 2.8 | Low |

Chr18: All database tissue targeted genes on chromosome 18

| Index | Gene | Tissue | TSE/TSP | Exp | Length |
| --- | --- | --- | --- | --- | --- |
| 1 | ADCYAP1 | brain | E | 10.7 | Short |
| 2 | LINC00470 | testis | P | 1.4 | Long |
| 3 | EMILIN2 | brain | E | 4.5 | Mid |
| 4 | MYOM1 | heart | E | 156.7 | Long |
| 5 | C18orf42 | brain | P | 21.2 | Mid |
| 6 | LINC00668 | testis | E | 14.4 | Short |
| 7 | GNAL | brain | E | 4.1 | Long |
| 8 | ANKRD62 | testis | E | 1.3 | Mid |
| 10 | CIDEA | adipose | E | 53.5 | Mid |
| 11 | LDLRAD4 | brain | E | 7.8 | Long |
| 12 | MC2R | adrenal | P | 20.6 | Mid |
| 15 | POTEC | testis | P | 7.9 | Mid |
| 16 | ANKRD30B | testis | E | 9.2 | Long |
| 17 | PSMA8 | testis | P | 13.4 | Mid |
| 18 | AQP4 | brain | E | 145.4 | Short |
| 19 | CHST9 | salivary gland | E | 7.5 | Long |
| 20 | DSC3 | skin | E | 125.2 | Mid |
| 21 | DSC2 | esophagus | E | 101.1 | Mid |
| 22 | DSC1 | skin | E | 103.0 | Mid |
| 23 | DSG1 | skin | E | 171.1 | Mid |
| 24 | DSG3 | esophagus | E | 211.0 | Mid |
| 25 | TTR | liver | P | 1516.9 | Short |
| 26 | RNF125 | adipose | E | 5.5 | Mid |
| 27 | MEP1B | sm intestine | P | 52.3 | Mid |
| 28 | KLHL14 | thyroid | E | 7.7 | Long |
| 29 | CCDC178 | testis | E | 5.2 | Long |
| 30 | ASXL3 | testis | E | 2.9 | Long |
| 31 | NOL4 | brain | E | 5.9 | Long |
| 32 | CELF4 | brain | E | 31.7 | Long |
| 33 | KC6 | prostate | Low | 0.2 | Long |
| 34 | RIT2 | brain | P | 30.4 | Long |
| 35 | SYT4 | brain | E | 123.4 | Short |
| 36 | RNF165 | brain | E | 6.6 | Long |
| 37 | LOXHD1 | testis | E | 7.3 | Long |
| 38 | ST8SIA5 | brain | E | 12.8 | Mid |
| 39 | SKOR2 | testis | E | 0.6 | Mid |
| 40 | ZBTB7C | esophagus | E | 16.0 | Long |
| 41 | LIPG | thyroid | E | 83.4 | Mid |
| 42 | MAPK4 | brain | E | 26.6 | Long |
| 43 | MRO | testis | E | 9.4 | Mid |
| 44 | DCC | testis | P | 4.1 | Long |
| 45 | DYNAP | esophagus | E | 9.1 | Short |
| 46 | RAB27B | esophagus | E | 8.0 | Mid |
| 47 | LINC-ROR | testis | Low | 0.1 | Mid |
| 48 | ST8SIA3 | brain | E | 15.6 | Mid |
| 49 | ONECUT2 | liver | E | 2.6 | Mid |
| 50 | CPLX4 | brain | E | 0.6 | Mid |
| 51 | CDH20 | brain | P | 10.7 | Long |
| 52 | TNFRSF11A | colon | E | 5.4 | Mid |
| 53 | SERPINB5 | skin | E | 124.2 | Mid |
| 54 | LINC00305 | testis | P | 2.8 | Short |
| 55 | CDH7 | brain | E | 2.3 | Long |
| 56 | CDH19 | nerve | E | 55.8 | Long |
| 57 | GTSCR1 | testis | P | 0.4 | Mid |
| 58 | CBLN2 | brain | P | 19.7 | Short |
| 59 | NETO1 | brain | E | 4.0 | Long |
| 60 | C18orf63 | testis | P | 4.6 | Mid |
| 61 | FAM69C | brain | E | 14.5 | Mid |
| 62 | CNDP1 | brain | E | 26.1 | Mid |
| 63 | SMIM21 | testis | P | 4.5 | Mid |
| 64 | GALR1 | pituitary | P | 1.5 | Mid |
| 65 | LINC01029 | testis | P | 0.8 | Mid |
| 66 | SALL3 | vagina | E | 3.0 | Mid |

Chr19: All database tissue targeted genes on chromosome 19

| Index | Gene | Tissue | TSE/TSP | Exp | Length |
| --- | --- | --- | --- | --- | --- |
| 1 | THEG | testis | P | 35.1 | Short |
| 3 | AZU1 | spleen | E | 6.6 | Short |
| 4 | ELANE | spleen | E | 11.1 | Short |
| 5 | EFNA2 | sm intestine | E | 9.3 | Mid |
| 6 | PLK5 | brain | E | 19.5 | Short |
| 7 | ONECUT3 | testis | Low | 1.3 | Mid |
| 8 | JSRP1 | muscle | E | 81.5 | Short |
| 9 | LINGO3 | brain | E | 15.8 | Short |
| 10 | ZNF556 | brain | E | 1.4 | Mid |
| 11 | TLE6 | thyroid | E | 4.3 | Short |
| 12 | SMIM24 | sm intestine | E | 189.8 | Short |
| 13 | TJP3 | colon | E | 34.8 | Mid |
| 14 | MATK | brain | E | 17.4 | Short |
| 15 | ZFR2 | pituitary | E | 14.7 | Mid |
| 16 | ATCAY | brain | E | 55.3 | Mid |
| 17 | CREB3L3 | liver | E | 205.7 | Mid |
| 18 | EBI3 | spleen | E | 31.0 | Short |
| 19 | SHD | heart | E | 7.8 | Short |
| 20 | TMIGD2 | spleen | E | 4.1 | Short |
| 22 | LRG1 | liver | E | 463.4 | Short |
| 24 | ARRDC5 | testis | E | 19.3 | Short |
| 25 | TINCR | skin | E | 41.1 | Short |
| 26 | CATSPERD | testis | P | 10.4 | Mid |
| 27 | FUT6 | esophagus | E | 26.2 | Short |
| 28 | FUT5 | colon | E | 5.1 | Short |
| 29 | ACER1 | skin | E | 58.1 | Mid |
| 30 | SLC25A41 | testis | E | 11.4 | Short |
| 31 | CRB3 | esophagus | E | 7.0 | Short |
| 32 | DENND1C | spleen | E | 35.3 | Short |
| 33 | TUBB4A | brain | E | 616.6 | Short |
| 34 | CD70 | spleen | E | 1.3 | Short |
| 35 | TNFSF14 | liver | E | 27.8 | Short |
| 36 | C3 | liver | E | 1009.1 | Short |
| 37 | VAV1 | spleen | E | 38.6 | Mid |
| 38 | ADGRE1 | spleen | E | 9.5 | Mid |
| 40 | C19orf45 | testis | P | 19.9 | Short |
| 41 | CAMSAP3 | skin | E | 47.1 | Mid |
| 42 | FCER2 | spleen | E | 30.8 | Short |
| 43 | CD209 | adipose | E | 6.4 | Short |
| 45 | CCL25 | sm intestine | P | 48.1 | Short |
| 46 | FBN3 | salivary gland | E | 2.0 | Mid |
| 47 | MUC16 | salivary gland | E | 1.4 | Long |
| 50 | AP1M2 | colon | E | 31.8 | Short |
| 51 | CCDC151 | testis | E | 12.4 | Short |
| 52 | ELAVL3 | brain | E | 103.7 | Mid |
| 53 | ACP5 | lung | E | 39.5 | Short |
| 54 | BEST2 | colon | E | 19.0 | Short |
| 55 | RTBDN | pituitary | E | 9.1 | Short |
| 56 | MAST1 | brain | E | 79.9 | Mid |
| 57 | KLF1 | spleen | Low | 0.7 | Short |
| 58 | DAND5 | heart | E | 3.2 | Short |
| 59 | RLN3 | testis | Low | 1.0 | Short |
| 61 | PALM3 | liver | E | 21.0 | Short |
| 62 | CLEC17A | spleen | E | 8.3 | Mid |
| 63 | ADGRE3 | spleen | Low | Low | Mid |
| 64 | ADGRE2 | spleen | E | 9.4 | Mid |
| 65 | SLC1A6 | brain | E | 36.4 | Mid |
| 66 | CCDC105 | testis | P | 15.7 | Short |
| 67 | CASP14 | skin | P | 285.4 | Short |
| 68 | RASAL3 | spleen | E | 35.2 | Short |
| 69 | CYP4F22 | skin | E | 38.9 | Mid |
| 70 | CYP4F8 | prostate | P | 2.3 | Short |
| 71 | CYP4F3 | liver | E | 76.1 | Mid |
| 72 | LINC00661 | testis | P | 37.8 | Short |
| 73 | LINC00905 | testis | P | 5.1 | Short |
| 74 | CIB3 | testis | Low | 0.3 | Short |
| 75 | NWD1 | brain | E | 4.5 | Mid |
| 76 | USHBP1 | heart | E | 5.9 | Mid |
| 77 | PLVAP | thyroid | E | 340.8 | Mid |
| 78 | NXNL1 | adipose | P | 1.0 | Short |
| 79 | UNC13A | brain | E | 36.2 | Mid |
| 80 | FCHO1 | skin | E | 10.2 | Mid |
| 81 | B3GNT3 | colon | E | 26.4 | Mid |
| 82 | INSL3 | testis | P | 99.8 | Short |
| 83 | SLC5A5 | stomach | E | 4.5 | Mid |
| 84 | KCNN1 | brain | P | 28.9 | Mid |
| 85 | IL12RB1 | spleen | E | 7.8 | Mid |
| 86 | LRRC25 | spleen | E | 48.3 | Short |
| 87 | NCAN | brain | P | 32.3 | Mid |
| 89 | LINC00664 | testis | E | 4.1 | Mid |
| 90 | ZNF208 | thyroid | E | 1.6 | Mid |
| 91 | ZNF676 | testis | E | 2.8 | Mid |
| 92 | ZNF729 | testis | Low | 1.1 | Mid |
| 93 | ZNF98 | testis | Low | 1.1 | Mid |
| 94 | VSTM2B | brain | E | 27.4 | Mid |
| 95 | ZNF536 | brain | E | 9.3 | Long |
| 96 | THEG5 | testis | P | 3.0 | Short |
| 97 | TDRD12 | testis | P | 7.9 | Long |
| 98 | SLC7A9 | sm intestine | E | 74.9 | Mid |
| 99 | CHST8 | pituitary | E | 43.6 | Long |
| 100 | LINC00904 | testis | P | 2.2 | Short |
| 101 | HPN | liver | E | 275.0 | Mid |
| 102 | MAG | brain | E | 366.3 | Mid |
| 103 | CD22 | spleen | E | 100.7 | Mid |
| 104 | PRODH2 | liver | E | 60.4 | Short |
| 105 | NPHS1 | kidney | E | 17.6 | Mid |
| 106 | KIRREL2 | pancreas | P | 20.0 | Short |
| 107 | RYR1 | muscle | E | 202.7 | Long |
| 108 | PAPL | brain | E | 8.5 | Mid |
| 112 | LEUTX | testis | Low | 0.1 | Short |
| 113 | FCGBP | colon | E | 342.9 | Mid |
| 115 | PCAT19 | lung | E | 12.0 | Mid |
| 116 | CEACAM21 | spleen | E | 6.8 | Short |
| 117 | LYPD4 | testis | P | 27.7 | Short |
| 118 | ATP1A3 | brain | E | 174.4 | Mid |
| 119 | PSG6 | placenta | P | 62.8 | Short |
| 120 | CD177 | bone marrow | E | 83.5 | Short |
| 121 | KCNN4 | salivary gland | E | 45.7 | Short |
| 122 | CEACAM20 | sm intestine | E | 13.3 | Mid |
| 123 | CBLC | skin | E | 30.2 | Mid |
| 125 | EXOC3L2 | thyroid | E | 21.0 | Mid |
| 126 | CKM | muscle | E | 11,938.1 | Mid |
| 128 | FOXA3 | liver | E | 19.1 | Short |
| 129 | NOVA2 | brain | E | 10.3 | Mid |
| 130 | IGFL2 | skin | E | 18.6 | Short |
| 132 | C5AR2 | spleen | E | 11.9 | Short |
| 133 | CRX | testis | Low | 0.1 | Mid |
| 134 | SULT2A1 | liver | E | 241.9 | Mid |
| 135 | BSPH1 | brain | P | 0.2 | Mid |
| 136 | CABP5 | spleen | Low | 0.1 | Short |
| 137 | CCDC155 | testis | E | 51.8 | Mid |
| 138 | SLC17A7 | brain | P | 278.8 | Short |
| 139 | TSKS | testis | P | 127.9 | Mid |
| 140 | SIGLEC11 | ovary | E | 35.1 | Short |
| 141 | IZUMO2 | testis | P | 29.7 | Short |
| 142 | SYT3 | brain | E | 14.3 | Mid |
| 144 | SIGLEC7 | spleen | E | 17.5 | Short |
| 145 | CD33 | spleen | E | 16.6 | Short |
| 146 | LIM2 | testis | Low | Low | Short |
| 147 | FPR3 | lung | E | 7.0 | Mid |
| 148 | NLRP12 | spleen | E | 3.7 | Mid |
| 149 | PRKCG | brain | E | 51.2 | Mid |
| 150 | VSTM1 | pituitary | E | 4.2 | Mid |
| 151 | KIR3DX1 | spleen | Low | 0.1 | Short |
| 152 | LILRA2 | spleen | E | 23.6 | Short |
| 153 | LILRB1 | spleen | E | 20.5 | Short |
| 154 | KIR3DL3 | testis | Low | 0.1 | Short |
| 155 | NCR1 | spleen | E | 2.2 | Short |
| 156 | NLRP7 | testis | P | 3.7 | Mid |
| 157 | SHISA7 | brain | E | 20.1 | Short |
| 158 | SBK2 | heart | E | 47.3 | Short |
| 160 | NLRP8 | testis | Low | 0.1 | Mid |
| 161 | GALP | testis | Low | 0.1 | Short |
| 162 | ZNF835 | brain | E | 3.2 | Short |
| 163 | ZIM2 | testis | E | 5.6 | Mid |
| 164 | PEG3 | ovary | E | 131.2 | Mid |
| 165 | MIMT1 | testis | E | 4.1 | Short |
| 166 | USP29 | testis | P | 3.4 | Short |
| 167 | DUXA | testis | Low | 0.1 | Short |
| 168 | ZSCAN1 | pituitary | E | 5.5 | Mid |

Chr20: All database tissue targeted genes on chromosome 20

| Index | Gene | Tissue | TSE/TSP | Exp | Length |
| --- | --- | --- | --- | --- | --- |
| 1 | DEFB125 | testis | Low | 0.2 | Short |
| 2 | SCRT2 | brain | P | 3.4 | Short |
| 3 | SLC52A3 | testis | E | 34.1 | Short |
| 4 | RSPO4 | lung | E | 7.6 | Mid |
| 5 | SIRPB2 | spleen | E | 10.4 | Mid |
| 6 | SIRPD | testis | P | 56.3 | Mid |
| 7 | PDYN | brain | E | 64.6 | Short |
| 8 | TGM3 | esophagus | E | 1080.6 | Mid |
| 9 | TGM6 | cervix | Low | 0.1 | Mid |
| 10 | SIGLEC1 | adipose | E | 11.3 | Mid |
| 11 | LINC00658 | testis | P | 21.8 | Short |
| 12 | CHGB | pituitary | E | 804.1 | Short |
| 13 | CASC20 | testis | Low | 0.1 | Mid |
| 14 | LAMP5-AS1 | brain | P | 3.6 | Short |
| 15 | PAK7 | brain | P | 14.2 | Long |
| 16 | ISM1 | thyroid | E | 35.7 | Mid |
| 17 | MACROD2 | brain | E | 3.0 | Long |
| 18 | PCSK2 | thyroid | E | 15.7 | Long |
| 19 | BANF2 | testis | P | 40.2 | Mid |
| 20 | OVOL2 | salivary gland | E | 6.9 | Mid |
| 21 | CFAP61 | testis | E | 6.3 | Long |
| 22 | PAX1 | thyroid | E | 0.4 | Short |
| 23 | LINC00261 | liver | E | 38.7 | Short |
| 25 | SYNDIG1 | brain | E | 24.4 | Long |
| 26 | VSX1 | brain | E | 8.5 | Short |
| 27 | FAM182B | testis | E | 5.5 | Long |
| 28 | FAM182A | testis | E | 1.0 | Mid |
| 29 | DEFB123 | testis | P | 98.9 | Short |
| 30 | TTLL9 | testis | E | 10.6 | Mid |
| 31 | XKR7 | brain | E | 13.0 | Mid |
| 32 | CCM2L | spleen | E | 77.9 | Mid |
| 33 | HCK | spleen | E | 93.0 | Mid |
| 34 | C20orf203 | brain | E | 2.2 | Mid |
| 35 | SUN5 | testis | P | 27.2 | Mid |
| 36 | BPIFB2 | salivary gland | P | 1117.3 | Mid |
| 37 | CNBD2 | testis | P | 18.9 | Mid |
| 38 | TLDC2 | sm intestine | E | 3.7 | Mid |
| 39 | GHRH | heart | E | 3.5 | Short |
| 40 | VSTM2L | brain | E | 69.4 | Mid |
| 41 | BPI | testis | E | 7.1 | Mid |
| 42 | LBP | liver | E | 452.6 | Mid |
| 43 | ARHGAP40 | skin | E | 15.7 | Mid |
| 44 | SLC32A1 | brain | E | 30.0 | Short |
| 45 | PPP1R16B | brain | E | 40.3 | Long |
| 46 | PTPRT | brain | P | 8.9 | Long |
| 47 | R3HDML | colon | E | 0.9 | Short |
| 48 | HNF4A | liver | E | 43.9 | Mid |
| 49 | KCNK15 | artery | E | 18.2 | Short |
| 50 | RIMS4 | brain | E | 33.4 | Mid |
| 51 | WFDC2 | salivary gland | E | 316.1 | Short |
| 52 | MMP9 | spleen | E | 70.5 | Short |
| 53 | SLC12A5 | brain | P | 78.4 | Mid |
| 55 | CDH22 | brain | E | 32.9 | Long |
| 56 | LINC00494 | testis | E | 6.2 | Short |
| 57 | KCNB1 | brain | E | 14.8 | Long |
| 58 | SALL4 | thyroid | E | 3.9 | Mid |
| 59 | TSHZ2 | ovary | E | 4.6 | Long |
| 60 | BCAS1 | brain | E | 282.6 | Long |
| 61 | CYP24A1 | bladder | E | 5.3 | Mid |
| 62 | TFAP2C | skin | E | 33.5 | Short |
| 63 | BMP7 | thyroid | E | 17.4 | Mid |
| 64 | SPO11 | testis | P | 5.6 | Short |
| 65 | CTCFL | testis | P | 3.5 | Mid |
| 66 | ZBP1 | spleen | E | 7.6 | Mid |
| 67 | C20orf85 | testis | P | 74.0 | Short |
| 68 | ZNF831 | spleen | E | 2.3 | Long |
| 69 | EDN3 | vagina | E | 49.9 | Mid |
| 70 | PHACTR3 | brain | E | 28.4 | Long |
| 71 | SYCP2 | testis | E | 19.6 | Mid |
| 72 | CDH26 | prostate | E | 3.8 | Mid |
| 73 | C20orf197 | spleen | Low | 0.4 | Mid |
| 74 | HRH3 | brain | E | 33.4 | Short |
| 75 | RBBP8NL | esophagus | E | 4.9 | Mid |
| 76 | GATA5 | bladder | E | 13.8 | Short |
| 77 | MIR1-1HG | colon | E | 21.0 | Mid |
| 78 | COL9A3 | brain | E | 37.5 | Mid |
| 79 | HAR1B | testis | E | 5.1 | Short |
| 80 | BIRC3 | sm intestine | E | 21.8 | Mid |
| 81 | NKAIN4 | brain | E | 23.2 | Short |
| 82 | COL20A1 | testis | E | 3.5 | Mid |
| 83 | CHRNA4 | liver | E | 15.1 | Mid |
| 84 | KCNQ2 | brain | E | 29.5 | Mid |
| 85 | EEF1A2 | muscle | E | 1138.1 | Short |
| 86 | SRMS | spleen | E | 0.7 | Short |
| 87 | MYT1 | brain | E | 28.7 | Mid |
| 88 | LINC00266-1 | brain | E | 1.3 | Mid |

Chr21: All database tissue targeted genes on chromosome 21

| Index | Gene | Tissue | TSE/TSP | Exp | Length |
| --- | --- | --- | --- | --- | --- |
| 1 | TPTE | testis | P | 57.8 | Mid |
| 4 | RBM11 | brain | E | 9.4 | Short |
| 6 | SAMSN1 | spleen | E | 16.8 | Mid |
| 7 | CHODL | testis | E | 19.0 | Mid |
| 8 | TMPRSS15 | sm intestine | P | 1.7 | Long |
| 9 | LINC00320 | brain | P | 12.6 | Mid |
| 10 | LINC00317 | testis | E | 0.1 | Short |
| 12 | LINC00314 | brain | P | 0.1 | Short |
| 13 | LINC00945 | brain | P | 9.0 | Short |
| 14 | CLIC6 | stomach | E | 94.3 | Mid |
| 15 | RIPPLY3 | prostate | E | 0.4 | Short |
| 16 | KCNJ6 | brain | E | 3.2 | Long |
| 17 | DSCR4 | testis | P | 0.4 | Long |
| 18 | DSCR8 | testis | P | 20.5 | Mid |
| 19 | KCNJ15 | kidney | E | 13.6 | Mid |
| 20 | ERG | artery | E | 15.7 | Long |
| 21 | B3GALT5-AS1 | brain | E | 1.8 | Short |
| 22 | B3GALT5 | sm intestine | E | 10.7 | Long |
| 23 | IGSF5 | heart | E | 1.7 | Mid |
| 24 | PCP4 | brain | E | 494.9 | Mid |
| 25 | DSCAM | brain | E | 4.1 | Long |
| 26 | LINC00111 | esophagus | Low | 0.1 | Mid |
| 27 | TMPRSS3 | salivary gland | E | 3.4 | Mid |
| 28 | UBASH3A | spleen | E | 3.4 | Mid |
| 29 | RSPH1 | testis | E | 40.1 | Mid |
| 30 | DNMT3L | kidney | E | 0.8 | Mid |
| 31 | AIRE | brain | E | 2.2 | Short |
| 32 | TRPM2 | kidney | E | 9.5 | Mid |
| 33 | ITGB2 | spleen | E | 90.6 | Mid |

Chr22: All database tissue targeted genes on chromosome 22

| Index | Gene | Tissue | TSE/TSP | Exp | Length |
| --- | --- | --- | --- | --- | --- |
| 1 | POTEH | testis | E | 1.7 | Mid |
| 2 | XKR3 | testis | P | 6.9 | Mid |
| 4 | CECR3 | testis | Low | 0.1 | Short |
| 7 | FAM230B | testis | P | 3.6 | Short |
| 9 | PRAME | testis | P | 69.1 | Short |
| 11 | IGLL5 | spleen | E | 560.9 | Short |
| 14 | IGLL1 | testis | P | 23.3 | Short |
| 17 | BCRP3 | kidney | E | 8.5 | Mid |
| 18 | PIWIL3 | testis | P | 0.4 | Mid |
| 20 | CRYBB2 | testis | E | 1.6 | Mid |
| 21 | CRYBB1 | spleen | E | 1.3 | Mid |
| 22 | SEC14L3 | liver | Low | 0.3 | Mid |
| 23 | SEC14L4 | liver | E | 8.7 | Mid |
| 24 | SLC5A1 | sm intestine | E | 120.1 | Mid |
| 25 | C22orf42 | testis | E | 16.2 | Short |
| 26 | SLC5A4 | sm intestine | E | 1.3 | Mid |
| 27 | BPIFC | skin | P | 20.1 | Mid |
| 28 | ISX | colon | E | 19.2 | Mid |
| 29 | MB | muscle | E | 3785.3 | Short |
| 30 | CACNG2 | brain | E | 6.5 | Long |
| 31 | PVALB | brain | E | 269.4 | Mid |
| 32 | NCF4 | spleen | E | 107.5 | Mid |
| 33 | CSF2RB | spleen | E | 19.1 | Mid |
| 34 | TEX33 | testis | P | 75.8 | Short |
| 35 | IL2RB | spleen | E | 20.9 | Mid |
| 36 | SSTR3 | testis | E | 3.5 | Short |
| 37 | KCNJ4 | brain | E | 56.2 | Mid |
| 39 | ENTHD1 | testis | E | 6.7 | Long |
| 40 | FAM83F | testis | E | 8.0 | Mid |
| 41 | SEPT3 | brain | E | 130.5 | Short |
| 42 | NFAM1 | spleen | E | 16.4 | Mid |
| 43 | SCUBE1 | nerve | E | 19.8 | Long |
| 44 | MPPED1 | brain | E | 20.8 | Mid |
| 45 | PNPLA5 | brain | E | 2.0 | Short |
| 46 | LINC00229 | testis | P | 10.8 | Mid |
| 47 | WNT7B | skin | E | 9.3 | Mid |
| 48 | LINC00898 | testis | P | 2.0 | Mid |
| 49 | FAM19A5 | nerve | E | 44.5 | Long |
| 50 | C22orf34 | spleen | E | 1.7 | Long |
| 52 | KLHDC7B | spleen | E | 2.5 | Short |
| 53 | ACR | testis | E | 75.5 | Short |

Chr23: All database tissue targeted genes on chromosome 23 (X chromosome)

| Index | Gene | Tissue | TSE/TSP | Exp | Length |
| --- | --- | --- | --- | --- | --- |
| 4 | SHOX | adipose | P | 0.2 | Mid |
| 5 | CRLF2 | appendix | E | 1.0 | Mid |
| 6 | CSF2RA | placenta | E | 20.8 | Mid |
| 7 | IL3RA | lung | E | 8.7 | Mid |
| 10 | P2RY8 | lymph node | E | 13.9 | Mid |
| 16 | XG | skin | E | 18.8 | Mid |
| 17 | ARSF | skin | E | 2.7 | Mid |
| 18 | NLGN4X | ovary | E | 7.7 | Long |
| 19 | KAL1 | brain | E | 5.8 | Long |
| 20 | FAM9A | testis | P | 4.4 | Short |
| 21 | FAM9B | testis | E | 1.9 | Long |
| 22 | TLR7 | brain | E | 1.0 | Mid |
| 23 | TLR8-AS1 | spleen | Low | 0.1 | Short |
| 24 | LINC01203 | testis | P | 2.8 | Mid |
| 25 | EGFL6 | lung | E | 16.9 | Mid |
| 26 | ASB9 | testis | E | 18.7 | Mid |
| 27 | ASB11 | heart | E | 11.7 | Mid |
| 28 | BMX | adipose | E | 5.0 | Mid |
| 29 | REPS2 | brain | E | 12.3 | Long |
| 31 | BEND2 | testis | E | 3.3 | Mid |
| 32 | PPEF1 | testis | E | 11.7 | Long |
| 33 | MAP7D2 | brain | E | 18.1 | Long |
| 34 | SMPX | heart | E | 176.3 | Mid |
| 35 | CFAP47 | pituitary | E | 0.9 | Mid |
| 38 | LANCL3 | solon | E | 0.6 | Long |
| 39 | XK | colon | E | 6.1 | Mid |
| 40 | CYBB | spleen | E | 43.6 | Mid |
| 41 | OTC | liver | E | 34.4 | Mid |
| 43 | CXorf36 | breast | E | 16.9 | Mid |
| 44 | SSX3 | testis | P | 10.0 | Short |
| 45 | GAGE13 | testis | P | 122.7 | Short |
| 46 | DGKK | adrenal | E | 2.2 | Long |
| 47 | SSX2B | testis | P | 18.4 | Mid |
| 48 | ITIH6 | prostate | E | 0.2 | Mid |
| 49 | LINC00269 | pituitary | E | 0.3 | Mid |
| 50 | FAM155B | heart | E | 26.4 | Mid |
| 51 | EDA | adrenal | E | 4.7 | Long |
| 52 | DGAT2L6 | skin | Low | 0.3 | Mid |
| 53 | CDX4 | skin | P | 0.4 | Short |
| 54 | KIAA2012 | testis | E | 1.0 | Long |
| 55 | CYSLTR1 | esophagus | E | 4.9 | Mid |
| 56 | P2RY10 | spleen | E | 5.9 | Mid |
| 58 | FAM46D | testis | P | 1.7 | Long |
| 59 | CYLC1 | testis | P | 28.8 | Mid |
| 60 | POF1B | skin | E | 84.9 | Long |
| 61 | KLHL4 | adrenal | E | 18.4 | Long |
| 62 | PCDH11X | brain | E | 1.8 | Long |
| 63 | NAP1L3 | brain | E | 104.2 | Short |
| 65 | PCDH19 | brain | E | 3.2 | Long |
| 66 | TNMD | adipose | E | 11.1 | Short |
| 67 | XKRX | skin | E | 5.5 | Mid |
| 68 | NXF5 | testis | E | 0.5 | Short |
| 70 | NXF2 | testis | P | 10.2 | Mid |
| 71 | NXF2B | testis | P | 9.5 | Mid |
| 73 | IL1RAPL2 | testis | Low | 0.2 | Long |
| 74 | MUM1L1 | ovary | E | 86.4 | Mid |
| 75 | CXorf57 | pituitary | E | 14.9 | Mid |
| 76 | NUP62CL | testis | E | 7.1 | Mid |
| 77 | PIH1D3 | testis | P | 7.3 | Mid |
| 78 | VSIG1 | testis | E | 55.1 | Mid |
| 79 | GUCY2F | testis | E | 0.3 | Long |
| 80 | CAPN9 | stomach | E | 12.3 | Mid |
| 81 | DCX | brain | E | 1.4 | Long |
| 82 | HTR2C | brain | P | 19.1 | Long |
| 83 | SLC6A14 | lung | E | 8.9 | Mid |
| 84 | LONRF3 | thyroid | E | 4.8 | Mid |
| 85 | KIAA1210 | testis | E | 23.7 | Mid |
| 86 | RHOXF1 | testis | E | 13.9 | Short |
| 87 | TMEM255A | testis | E | 12.4 | Mid |
| 88 | ATP1B4 | muscle | P | 4.6 | Mid |
| 89 | CT47B1 | testis | P | 7.3 | Short |
| 90 | GRIA3 | brain | E | 19.9 | Long |
| 91 | SH2D1A | spleen | E | 5.1 | Mid |
| 92 | TENM1 | brain | E | 15.4 | Long |
| 93 | ARHGAP36 | pituitary | E | 139.9 | Mid |
| 94 | STK26 | bone marrow | E | 21.7 | Mid |
| 95 | FRMD7 | uterus | E | 1.8 | Mid |
| 96 | HS6ST2 | ovary | E | 7.5 | Long |
| 97 | GPC3 | lung | E | 74.6 | Long |
| 98 | CT45A1 | testis | P | 10.8 | Short |
| 99 | CT45A4 | testis | P | 11.8 | Short |
| 100 | ADGRG4 | fallopian tube | E | 1.0 | Long |
| 101 | VGLL1 | bladder | E | 5.8 | Mid |
| 102 | FGF13 | brain | E | 23.8 | Mid |
| 103 | F9 | liver | P | 151.9 | Mid |
| 104 | CXorf66 | testis | P | 14.0 | Short |
| 105 | SPANXN3 | testis | P | 38.0 | Short |
| 106 | FMR1NB | testis | P | 52.3 | Mid |
| 107 | AFF2 | brain | E | 4.4 | Long |
| 108 | LINC00850 | adipose | E | 0.2 | Mid |
| 109 | PASD1 | testis | P | 13.7 | Long |
| 110 | FATE1 | testis | P | 273.2 | Short |
| 111 | GABRA3 | brain | P | 10.4 | Long |
| 112 | ATP2B3 | brain | E | 15.3 | Mid |
| 113 | OPN1LW | testis | Low | 0.1 | Short |
| 114 | OPN1MW2 | adipose | Low | 0.1 | Short |
| 115 | TMLHE-AS1 | spleen | Low | 0.1 | Long |
| 117 | IL9R | bladder | Low | 0.5 | Short |

Chr24: All database tissue targeted genes on chromosome 24 (Y chromosome)

| Index | Gene | Tissue | TSE/TSP | Exp | Length |
| --- | --- | --- | --- | --- | --- |
| 1 | PLCXD1 | stomach | E | 5.3 | Chr23 |
| 2 | PPP2R3B | heart | E | 9.0 | Chr23 |
| 3 | SHOX | adipose | P | 0.2 | Chr23 |
| 4 | CRLF2 | appendix | E | 1.0 | Chr23 |
| 5 | CSF2RA | placenta | E | 20.8 | Chr23 |
| 6 | IL3RA | lung | E | 8.7 | Chr23 |
| 11 | P2RY8 | lymph node | E | 13.9 | Chr23 |
| 18 | SRY | testis | E | 2.0 | Short |
| 19 | ZFY | testis | E | 3.4 | Mid |
| 20 | LINC00278 | sm intestine | E | 1.0 | Long |
| 20a | TGIF2LY | testis | P | 4.4 | Short |
| 21 | PCDH11Y | brain | E | 1.4 | Mid |
| 22 | TSPY2 | testis | P | 5.7 | Short |
| 23 | TTTY1B | gall bladder | E | 0.8 | Mid |
| 24 | TTTY2B | gall bladder | E | 3.8 | Mid |
| 25 | AMELY | prostate | E | 0.4 | Mid |
| 25a | TBL1Y | prostate | E | 0.5 | Long |
| 26 | TTTY11 | testis | P | 0.1 | Mid |
| 27 | TSPY8 | testis | P | 34.5 | Short |
| 28 | TSPY1 | testis | P | 33.8 | Short |
| 29 | TTTY2 | gall bladder | E | 3.9 | Mid |
| 30 | TTTY1 | gall bladder | E | 0.8 | Mid |
| 32b | NLGN4Y | brain | E | 2.2 | Long |
| 34 | HSFY1 | testis | P | 0.1 | Mid |
| 35 | HSFY2 | brain | E | 0.2 | Short |
| 35a | TTTY14 | brain | E | 4.6 | Long |
| 38 | TTTY10 | spleen | E | 0.6 | Long |
| 40 | RBMY1A1 | testis | P | 0.5 | Mid |
| 41 | PRY | testis | Low | 0.6 | Mid |
| 42 | RBMY1F | testis | P | 0.1 | Short |
| 43 | RBMY1J | testis | low | 0.3 | Short |
| 44 | TTTY4 | kidney | low | 0.1 | Mid |
| 45 | BPY2 | testis | low | 0.1 | Mid |
| 46 | DAZ1 | testis | P | 0.5 | Mid |
| 47 | DAZ2 | testis | P | 0.3 | Mid |
| 48 | TTTY4B | kidney | P | 0.1 | Mid |
| 49 | BPY2B | adipose | low | 0.1 | Mid |
| 50 | DAZ3 | testis | low | 0.1 | Mid |
| 51 | DAZ4 | testis | low | 0.1 | Mid |
