# Supplementary material for: Role of Gene Length in Control of Human Gene Expression: Chromosome-Specific and Tissue-Specific Effects
Source: Int J Genomics. 2021 Feb 13;2021:8902428. doi: 10.1155/2021/8902428 (PMC7911607; doi:10.1155/2021/8902428)
Supplement: Supplementary 2 — Table S2: list of all 1125 broadly expressed genes together with the same information described above except for information about tissue expression. [file 8902428.f2.docx]

**Supplementary Data**. Table S2: All broadly-expressed database genes

Chr: Gene chromosome number.

Length: Gene Length in Mb.

Exp: Gene Expression Level. Units are TPM. Data were downloaded from the UCSC Genome Browser ( <http://genome.ucsc.edu> ).

S/M/L: Gene length group. Short, Mid-Length or Long. S/M/L gene lengths are <15kb, 15kb-100kb and >100kb, respectively.

| Chr | Gene | Length | Exp | S/M/L |
| --- | --- | --- | --- | --- |
| 1 | ACBD6 | 233.3 | 23.4 | Long |
| 6 | ANKS1A | 202.2 | 75.6 | Long |
| 20 | ARFGEF2 | 115.0 | 27.2 | Long |
| 1 | ATF6 | 197.8 | 20.0 | Long |
| 1 | KIAA0319L | 123.9 | 30.0 | Long |
| 2 | CAB39 | 108.2 | 79.9 | Long |
| 16 | CFDP1 | 139.8 | 83.1 | Long |
| 16 | CREBBP | 155.7 | 40.5 | Long |
| 15 | CRTC3 | 115.4 | 51.0 | Long |
| 20 | CTNNBL1 | 178.1 | 31.1 | Long |
| 7 | CUL1 | 102.9 | 72.5 | Long |
| 2 | CUL3 | 115.2 | 20.6 | Long |
| 14 | DCAF5 | 102.2 | 40.3 | Long |
| 10 | DNAJC1 | 247.2 | 46.8 | Long |
| 3 | DNAJC13 | 121.5 | 32.8 | Long |
| 13 | DNAJC3 | 117.9 | 37.4 | Long |
| 3 | EEFSEC | 255.2 | 28.7 | Long |
| 8 | EIF3E | 126.3 | 368.6 | Long |
| 5 | ERGIC1 | 118.4 | 77.0 | Long |
| 1 | ERI3 | 134.2 | 75.2 | Long |
| 9 | ERP44 | 119.8 | 35.4 | Long |
| 6 | EXOC2 | 208.0 | 11.0 | Long |
| 11 | FAM168A | 192.2 | 82.2 | Long |
| 1 | FBXO42 | 105.6 | 27.9 | Long |
| 1 | GATAD2B | 118.3 | 21.2 | Long |
| 10 | GBF1 | 137.4 | 70.4 | Long |
| 1 | GNB1 | 105.8 | 325.9 | Long |
| 3 | GOLGA4 | 123.6 | 50.8 | Long |
| 13 | GTF2F2 | 164.4 | 29.9 | Long |
| 11 | HSD17B12 | 175.9 | 26.4 | Long |
| 4 | HTT | 169.3 | 37.6 | Long |
| 15 | INO80 | 137.3 | 27.8 | Long |
| 3 | IQSEC1 | 176.1 | 100.7 | Long |
| 20 | ITCH | 148.2 | 37.7 | Long |
| 1 | JAK1 | 133.3 | 110.1 | Long |
| 11 | KDM2A | 138.8 | 54.1 | Long |
| 19 | KDM4B | 184.5 | 28.7 | Long |
| 5 | LARP1 | 104.7 | 60.3 | Long |
| 10 | LARP4B | 124.8 | 27.2 | Long |
| 3 | LARS | 160.3 | 9.9 | Long |
| 16 | LONP2 | 109.2 | 33.9 | Long |
| 2 | LRPPRC | 109.8 | 59.7 | Long |
| 1 | MAN1A2 | 158.1 | 17.0 | Long |
| 15 | MAP2K1 | 104.7 | 87.1 | Long |
| 22 | MAPK1 | 108.0 | 42.5 | Long |
| 17 | MED13 | 122.7 | 20.6 | Long |
| 8 | MRPS28 | 111.4 | 20.6 | Long |
| 1 | MTOR | 156.0 | 19.8 | Long |
| 13 | MYCBP2 | 282.4 | 28.5 | Long |
| 5 | NDUFAF2 | 207.9 | 46.5 | Long |
| 10 | NT5C2 | 105.3 | 48.4 | Long |
| 11 | PACS1 | 174.4 | 64.6 | Long |
| 22 | PACSIN2 | 145.4 | 120.2 | Long |
| 16 | PARN | 194.6 | 26.4 | Long |
| 4 | PDS5A | 155.1 | 28.8 | Long |
| 14 | PPP2R5C | 166.2 | 23.0 | Long |
| 14 | PPP2R5E | 168.7 | 23.5 | Long |
| 22 | PPP6R2 | 101.8 | 63.3 | Long |
| 6 | PREP | 125.6 | 24.0 | Long |
| 2 | PSMD1 | 116.0 | 99.9 | Long |
| 2 | PSMD14 | 103.5 | 64.7 | Long |
| 10 | PTEN | 105.3 | 47.6 | Long |
| 8 | RAB2A | 106.7 | 96.9 | Long |
| 2 | RAB3GAP1 | 118.4 | 31.8 | Long |
| 1 | RERE | 465.2 | 92.2 | Long |
| 7 | RNF216 | 161.7 | 34.5 | Long |
| 4 | SEC24B | 106.6 | 34.9 | Long |
| 6 | SMAP1 | 194.2 | 13.5 | Long |
| 3 | SMARCC1 | 196.0 | 41.9 | Long |
| 7 | SND1 | 440.5 | 120.9 | Long |
| 17 | SPAG9 | 158.7 | 85.3 | Long |
| 15 | SPAG11 | 101.0 | 16.9 | Long |
| 12 | SPPL3 | 141.8 | 32.8 | Long |
| 11 | STIM1 | 237.5 | 54.4 | Long |
| 13 | STK24 | 126.9 | 49.3 | Long |
| 3 | STT3B | 103.1 | 77.8 | Long |
| 17 | STX8 | 325.5 | 23.1 | Long |
| 17 | TBCD | 191.1 | 30.4 | Long |
| 3 | TBL1XR1 | 176.5 | 31.5 | Long |
| 9 | TMEM245 | 104.8 | 45.6 | Long |
| 8 | TNKS | 226.4 | 13.2 | Long |
| 2 | TRIP12 | 154.7 | 41.8 | Long |
| 9 | UBE2R2 | 103.2 | 61.1 | Long |
| 7 | UBE3C | 130.4 | 34.8 | Long |
| 1 | UBR4 | 135.8 | 43.2 | Long |
| 1 | USP48 | 104.9 | 26.6 | Long |
| 8 | ZC3H3 | 103.8 | 23.7 | Long |
| 10 | ZMIZ1 | 247.5 | 40.8 | Long |
| 1 | ZYG11B | 100.9 | 16.5 | Long |

| 16 | C16orf72 | 30.0 | 31.2 | Mid |
| --- | --- | --- | --- | --- |
| 17 | KIAA0100 | 30.7 | 57.0 | Mid |
| 20 | AAR2 | 20.5 | 44.4 | Mid |
| 16 | AARS | 37.3 | 173.4 | Mid |
| 11 | AASDHPPT | 21.0 | 83.0 | Mid |
| 4 | ABCE1 | 31.2 | 41.6 | Mid |
| 7 | ABCF2 | 15.7 | 47.9 | Mid |
| 3 | ACAD9 | 33.5 | 77.7 | Mid |
| 1 | ACBD3 | 42.1 | 36.5 | Mid |
| 10 | ACTR1A | 23.5 | 117.5 | Mid |
| 2 | ACTR2 | 43.5 | 146.2 | Mid |
| 4 | ADH5 | 17.8 | 249.2 | Mid |
| 12 | ADIPOR2 | 97.6 | 113.5 | Mid |
| 7 | AGAP3 | 58.0 | 135.5 | Mid |
| 5 | AGGF1 | 34.8 | 19.9 | Mid |
| 1 | AIDA | 44.5 | 32.3 | Mid |
| 1 | AK2 | 28.9 | 41.3 | Mid |
| 9 | AK3 | 31.6 | 67.7 | Mid |
| 6 | AKIRIN2 | 27.4 | 76.9 | Mid |
| 14 | AKT1 | 26.4 | 88.8 | Mid |
| 1 | ALDH9A1 | 36.7 | 130.5 | Mid |
| 17 | ALKBH5 | 26.1 | 166.6 | Mid |
| 16 | AMFR | 64.1 | 56.2 | Mid |
| 17 | ANKFY1 | 97.7 | 41.7 | Mid |
| 12 | ANKLE2 | 36.3 | 72.1 | Mid |
| 13 | ANKRD10 | 36.5 | 134.6 | Mid |
| 12 | ANKRD52 | 20.6 | 44.0 | Mid |
| 15 | ANP32A | 42.4 | 75.9 | Mid |
| 9 | ANP32B | 32.6 | 284.0 | Mid |
| 16 | AP1G1 | 80.2 | 34.9 | Mid |
| 10 | AP3M1 | 30.8 | 27.4 | Mid |
| 15 | AP3S2 | 63.4 | 16.7 | Mid |
| 3 | API5 | 32.5 | 78.4 | Mid |
| 3 | APPL1 | 45.7 | 28.3 | Mid |
| 11 | ARCN1 | 30.6 | 132.2 | Mid |
| 1 | ARF1 | 16.6 | 423.3 | Mid |
| 3 | ARF4 | 26.0 | 232.4 | Mid |
| 22 | ARFGAP3 | 60.8 | 40.9 | Mid |
| 11 | ARHGAP1 | 23.5 | 232.4 | Mid |
| 3 | ARIH2 | 67.6 | 39.0 | Mid |
| 11 | ARL14EP | 15.1 | 13.9 | Mid |
| 3 | ARL8B | 58.7 | 112.5 | Mid |
| 8 | ARMC1 | 31.7 | 39.9 | Mid |
| 1 | ARNT | 66.9 | 68.8 | Mid |
| 2 | ARPC2 | 37.2 | 250.6 | Mid |
| 15 | ARPP19 | 22.2 | 173.8 | Mid |
| 21 | ASNA1/GET3 | 18.2 | 37.9 | Mid |
| 20 | ASXL1 | 81.0 | 61.1 | Mid |
| 3 | ATG3 | 29.5 | 51.2 | Mid |
| 23 | ATP6GAP2 | 25.9 | 154.4 | Mid |
| 5 | ATP6V0E1 | 51.7 | 236.4 | Mid |
| 1 | ATPAF1 | 35.7 | 66.3 | Mid |
| 7 | BAZ1B | 81.9 | 51.7 | Mid |
| 6 | C6orf89 | 37.7 | 72.4 | Mid |
| 23 | BCAP31 | 23.9 | 189.3 | Mid |
| 10 | BCCIP | 30.2 | 23.5 | Mid |
| 16 | BFAR | 36.3 | 38.5 | Mid |
| 8 | BNIP3L | 30.1 | 152.9 | Mid |
| 9 | BRD3 | 37.7 | 36.0 | Mid |
| 19 | BRD4 | 44.9 | 36.1 | Mid |
| 1 | BSDC1 | 29.4 | 78.8 | Mid |
| 15 | BTBD1 | 50.8 | 40.1 | Mid |
| 19 | BTBD2 | 30.3 | 142.7 | Mid |
| 1 | BTF3L4 | 34.4 | 26.8 | Mid |
| 10 | BUB3 | 16.1 | 25.4 | Mid |
| 19 | CACTIN | 16.2 | 32.6 | Mid |
| 12 | CAND1 | 50.6 | 26.6 | Mid |
| 17 | CASC3 | 31.6 | 139.0 | Mid |
| 17 | CBX1 | 31.4 | 102.6 | Mid |
| 22 | CBY1 | 17.2 | 61.9 | Mid |
| 8 | CCAR2 | 16.9 | 71.5 | Mid |
| 23 | CCDC22 | 15.1 | 36.3 | Mid |
| 17 | CCDC47 | 28.3 | 62.0 | Mid |
| 3 | CCDC50 | 69.3 | 41.1 | Mid |
| 4 | CCNI | 28.8 | 454.6 | Mid |
| 14 | CCNK | 31.0 | 16.6 | Mid |
| 12 | CCT2 | 16.1 | 138.9 | Mid |
| 2 | CCT4 | 20.6 | 123.7 | Mid |
| 7 | CCZ1 | 27.8 | 13.6 | Mid |
| 10 | CDC123 | 54.4 | 72.2 | Mid |
| 13 | CDC16 | 37.8 | 73.9 | Mid |
| 5 | CDC23 | 25.7 | 21.4 | Mid |
| 9 | CDC37L1 | 28.8 | 40.5 | Mid |
| 1 | CDC42 | 38.1 | 124.9 | Mid |
| 1 | CDC42SE1 | 19.4 | 234.0 | Mid |
| 6 | CDC5L | 62.7 | 20.3 | Mid |
| 2 | CEBPZ | 30.0 | 36.2 | Mid |
| 2 | CEBPZOS | 17.2 | 33.6 | Mid |
| 3 | CGGBP1 | 94.5 | 46.1 | Mid |
| 3 | CHMP2B | 28.3 | 66.4 | Mid |
| 2 | CHMP3 | 60.0 | 63.1 | Mid |
| 20 | CHMP4B | 43.0 | 206.2 | Mid |
| 15 | CHP1 | 50.6 | 91.8 | Mid |
| 14 | CIPC | 19.0 | 74.5 | Mid |
| 11 | CLNS1A | 23.3 | 57.1 | Mid |
| 19 | CLPTM1 | 37.9 | 134.3 | Mid |
| 5 | CLPTM1L | 27.4 | 92.0 | Mid |
| 12 | CMAS | 19.5 | 105.4 | Mid |
| 1 | CMPK1 | 45.0 | 125.6 | Mid |
| 6 | CMTR1 | 48.3 | 56.0 | Mid |
| 2 | CNOT11 | 17.4 | 58.7 | Mid |
| 13 | COG3 | 71.8 | 25.8 | Mid |
| 16 | COG7 | 64.7 | 44.3 | Mid |
| 20 | COMMD7 | 41.3 | 71.8 | Mid |
| 22 | COMT | 28.4 | 74.7 | Mid |
| 1 | COPA | 54.7 | 134.3 | Mid |
| 3 | COPB2 | 32.2 | 87.4 | Mid |
| 19 | COPE | 19.9 | 209.1 | Mid |
| 3 | COPG1 | 28.2 | 130.4 | Mid |
| 4 | COPS4 | 40.6 | 41.1 | Mid |
| 8 | COPS5 | 19.1 | 54.5 | Mid |
| 12 | COPZ1 | 26.7 | 102.3 | Mid |
| 12 | COQ5 | 25.9 | 42.2 | Mid |
| 10 | COX15 | 18.6 | 33.4 | Mid |
| 17 | CRK | 35.5 | 69.5 | Mid |
| 22 | CRKL | 36.3 | 69.1 | Mid |
| 5 | CSNK1A1 | 58.5 | 105.9 | Mid |
| 17 | CSNK1D | 31.1 | 105.7 | Mid |
| 20 | CSNK2A1 | 71.3 | 39.1 | Mid |
| 16 | CSNK2A2 | 40.2 | 73.7 | Mid |
| 4 | CTBP1 | 37.7 | 63.1 | Mid |
| 3 | CTNNB1 | 45.2 | 233.5 | Mid |
| 23 | CUL4B | 49.2 | 40.2 | Mid |
| 16 | CYB5B | 41.7 | 63.9 | Mid |
| 6 | C6orf70 | 30.0 | 36.2 | Mid |
| 14 | DAD1 | 24.3 | 346.5 | Mid |
| 1 | DAP3 | 49.9 | 47.0 | Mid |
| 2 | DARS | 79.7 | 61.1 | Mid |
| 9 | DCAF12 | 40.3 | 38.5 | Mid |
| 12 | DCTN2 | 16.8 | 158.4 | Mid |
| 5 | DCTN4 | 50.7 | 41.3 | Mid |
| 11 | DDB1 | 33.9 | 126.6 | Mid |
| 2 | DDX1 | 39.9 | 97.2 | Mid |
| 22 | DDX17 | 22.9 | 643.6 | Mid |
| 2 | DDX18 | 17.7 | 54.7 | Mid |
| 16 | DDX19A | 26.5 | 37.7 | Mid |
| 12 | DDX23 | 22.4 | 66.8 | Mid |
| 20 | DDX27 | 24.8 | 63.3 | Mid |
| 12 | DDX47 | 16.6 | 2.0 | Mid |
| 11 | DEAF1 | 51.3 | 95.9 | Mid |
| 3 | DENND6A | 67.6 | 20.7 | Mid |
| 12 | DENR | 18.2 | 47.2 | Mid |
| 8 | DERL1 | 29.1 | 56.8 | Mid |
| 4 | DHX15 | 57.1 | 64.8 | Mid |
| 5 | DHX29 | 51.6 | 31.1 | Mid |
| 16 | DHX38 | 19.1 | 73.7 | Mid |
| 17 | DHX40 | 42.7 | 39.7 | Mid |
| 5 | DIMT1 | 16.7 | 32.9 | Mid |
| 16 | DNAJA2 | 18.3 | 44.3 | Mid |
| 16 | DNAJA3 | 30.9 | 53.5 | Mid |
| 3 | DNAJB11 | 18.4 | 91.3 | Mid |
| 7 | DNAJB6 | 49.4 | 53.8 | Mid |
| 1 | DNAJC11 | 67.6 | 15.8 | Mid |
| 20 | DNAJC5 | 40.9 | 164.6 | Mid |
| 17 | DNAJC7 | 41.0 | 72.3 | Mid |
| 1 | DNAJC8 | 32.8 | 84.0 | Mid |
| 20 | DNTTIP1 | 19.5 | 48.8 | Mid |
| 2 | DPY30 | 15.9 | 56.3 | Mid |
| 22 | DRG1 | 34.9 | 76.4 | Mid |
| 2 | DUSP11 | 18.0 | 20.5 | Mid |
| 3 | DYNC1LI1 | 44.9 | 58.3 | Mid |
| 20 | DYNLRB1 | 24.6 | 135.2 | Mid |
| 14 | EAPP | 23.8 | 67.3 | Mid |
| 8 | EBAG9 | 25.8 | 24.2 | Mid |
| 15 | EDC3 | 65.5 | 24.6 | Mid |
| 8 | EEF1D | 18.0 | 131.2 | Mid |
| 23 | EIF1AX | 17.3 | 82.9 | Mid |
| 7 | EIF2AK1 | 37.0 | 62.8 | Mid |
| 1 | EIF2D | 20.8 | 55.0 | Mid |
| 14 | EIF2S1 | 26.2 | 36.5 | Mid |
| 20 | EIF2S2 | 23.9 | 65.6 | Mid |
| 10 | EIF3A | 47.2 | 119.1 | Mid |
| 7 | EIF3B | 25.9 | 148.3 | Mid |
| 22 | EIF3D | 18.3 | 190.4 | Mid |
| 19 | EIF3K | 17.8 | 187.4 | Mid |
| 11 | EIF3M | 23.4 | 67.1 | Mid |
| 12 | EIF4B | 36.1 | 520.6 | Mid |
| 7 | EIF4H | 22.9 | 272.8 | Mid |
| 2 | EIF5B | 63.9 | 71.5 | Mid |
| 19 | ELAVL1 | 47.1 | 33.6 | Mid |
| 19 | ELL | 79.4 | 29.5 | Mid |
| 8 | EMC2 | 45.6 | 93.6 | Mid |
| 3 | EMC3 | 24.6 | 76.3 | Mid |
| 15 | EMC7 | 17.9 | 101.7 | Mid |
| 16 | EMC8 | 20.9 | 19.4 | Mid |
| 19 | EPN1 | 34.3 | 30.8 | Mid |
| 20 | ERGIC3 | 15.6 | 197.1 | Mid |
| 14 | ERH | 18.2 | 122.9 | Mid |
| 2 | ERLEC1 | 31.8 | 105.6 | Mid |
| 5 | ETF1 | 37.1 | 87.2 | Mid |
| 5 | FAF2 | 61.7 | 29.9 | Mid |
| 10 | FAM160B1 | 42.9 | 27.7 | Mid |
| 2 | FAM168B | 45.6 | 129.6 | Mid |
| 16 | FAM192A | 33.6 | 6+0.6 | Mid |
| 1 | FAM20B | 50.7 | 45.8 | Mid |
| 2 | FAM98A | 15.6 | 43.3 | Mid |
| 13 | FBXL3 | 21.9 | 92.0 | Mid |
| 1 | FBXO28 | 47.9 | 30.8 | Mid |
| 10 | FBXW4 | 84.6 | 79.1 | Mid |
| 14 | FCF1 | 25.5 | 21.4 | Mid |
| 15 | FEM1B | 18.1 | 52.0 | Mid |
| 17 | FLCN | 25.0 | 108.5 | Mid |
| 4 | FRG1 | 21.0 | 39.6 | Mid |
| 9 | FUBP3 | 58.8 | 30.1 | Mid |
| 17 | FXR2 | 23.7 | 93.4 | Mid |
| 19 | FZR1 | 32.1 | 72.5 | Mid |
| 5 | G3BP1 | 40.9 | 32.5 | Mid |
| 4 | G3BP2 | 31.2 | 87.4 | Mid |
| 10 | GDI2 | 48.2 | 116.2 | Mid |
| 7 | GET4 | 19.9 | 6.1 | Mid |
| 16 | GGA2 | 47.0 | 97.9 | Mid |
| 9 | GLE1 | 37.6 | 35.0 | Mid |
| 1 | GNL2 | 29.1 | 19.2 | Mid |
| 1 | GNPAT | 36.8 | 65.5 | Mid |
| 12 | GOLGA3 | 60.0 | 37.5 | Mid |
| 5 | GOLPH3 | 49.6 | 133.1 | Mid |
| 2 | GORASP2 | 37.9 | 76.9 | Mid |
| 17 | GOSR2 | 43.9 | 20.2 | Mid |
| 5 | GPBP1 | 90.6 | 78.8 | Mid |
| 19 | GPI | 37.2 | 310.4 | Mid |
| 12 | GPN3 | 15.8 | 36.0 | Mid |
| 16 | GSPT1 | 43.0 | 42.3 | Mid |
| 1 | GTF2B | 38.9 | 33.6 | Mid |
| 8 | GTF2E2 | 79.9 | 42.3 | Mid |
| 2 | GTF3C3 | 36.7 | 14.1 | Mid |
| 22 | GTPBP1 | 27.6 | 42.7 | Mid |
| 2 | HAT1 | 61.2 | 35.0 | Mid |
| 23 | HCFC1 | 24.2 | 38.2 | Mid |
| 18 | IER3IP1 | 23.5 | 26.4 | Mid |
| 2 | HDLBP | 88.4 | 161.2 | Mid |
| 7 | HERPUD2 | 62.5 | 51.3 | Mid |
| 17 | HGS | 18.1 | 88.7 | Mid |
| 3 | HIGD1A | 20.2 | 283.3 | Mid |
| 1 | HIPK1 | 48.6 | 59.9 | Mid |
| 5 | HMGXB3 | 52.4 | 54.5 | Mid |
| 7 | HNRNPA2B1 | 28.3 | 889.5 | Mid |
| 4 | HNRNPD | 21.5 | 193.2 | Mid |
| 19 | HNRNPM | 44.1 | 187.8 | Mid |
| 19 | HNRNPUL1 | 43.4 | 112.4 | Mid |
| 1 | HP1BP3 | 44.0 | 153.2 | Mid |
| 8 | HSF1 | 23.1 | 123.0 | Mid |
| 12 | HSP90B1 | 17.6 | 800.8 | Mid |
| 5 | HSPA4 | 53.1 | 49.4 | Mid |
| 23 | IGBP1 | 32.9 | 180.9 | Mid |
| 2 | ILKAP | 33.3 | 59.7 | Mid |
| 8 | INTS10 | 34.7 | 67.2 | Mid |
| 4 | INTS12 | 26.1 | 15.6 | Mid |
| 13 | IPO5 | 70.6 | 67.1 | Mid |
| 12 | IPO8 | 67.0 | 32.6 | Mid |
| 1 | IPO9 | 55.1 | 26.8 | Mid |
| 2 | IWS1 | 45.7 | 37.8 | Mid |
| 16 | KARS | 20.0 | 114.4 | Mid |
| 7 | KBTBD2 | 23.7 | 35.5 | Mid |
| 2 | KCMF1 | 88.4 | 34.6 | Mid |
| 6 | KCTD20 | 47.8 | 53.0 | Mid |
| 16 | KCTD5 | 26.5 | 35.6 | Mid |
| 7 | KDELR2 | 23.1 | 226.8 | Mid |
| 1 | KDM1A | 64.2 | 71.9 | Mid |
| 19 | KEAP1 | 17.3 | 88.3 | Mid |
| 1 | KHDRBS1 | 30.2 | 150.6 | Mid |
| 10 | KIF1BP | 27.8 | 35.8 | Mid |
| 7 | KLHDC10 | 65.2 | 24.5 | Mid |
| 3 | KPNA1 | 93.0 | 21.6 | Mid |
| 13 | KPNA3 | 93.6 | 33.6 | Mid |
| 3 | KPNA4 | 70.6 | 34.1 | Mid |
| 1 | KPNA6 | 68.5 | 41.5 | Mid |
| 12 | KRAS | 45.7 | 21.5 | Mid |
| 23 | LAMP2 | 43.2 | 60.7 | Mid |
| 16 | LCMT1 | 66.5 | 40.5 | Mid |
| 18 | LMAN1 | 31.5 | 66.7 | Mid |
| 5 | LMAN2 | 20.3 | 180.0 | Mid |
| 19 | LONP1 | 28.4 | 61.6 | Mid |
| 4 | LRPAP1 | 28.9 | 54.0 | Mid |
| 1 | LRRC41 | 25.0 | 38.1 | Mid |
| 17 | LRRC59 | 16.3 | 78.4 | Mid |
| 17 | LSM12 | 33.0 | 6.6 | Mid |
| 19 | LSM14A | 57.1 | 90.9 | Mid |
| 19 | LSM4 | 17.0 | 56.2 | Mid |
| 6 | LTV1 | 20.4 | 40.4 | Mid |
| 17 | LUC7L3 | 33.2 | 242.2 | Mid |
| 4 | MAEA | 50.3 | 52.3 | Mid |
| 19 | MAP2K2 | 33.8 | 108.1 | Mid |
| 14 | MAPK1IP1L | 18.6 | 35.2 | Mid |
| 16 | MAPK8IP3 | 64.1 | 108.1 | Mid |
| 20 | MAPRE1 | 30.5 | 118.2 | Mid |
| 10 | MARCH5 | 62.8 | 31.7 | Mid |
| 19 | MAU2 | 37.9 | 57.3 | Mid |
| 14 | MAX | 27.4 | 88.0 | Mid |
| 18 | MBD2 | 73.2 | 45.8 | Mid |
| 5 | MCCC2 | 71.4 | 35.7 | Mid |
| 10 | MCMBP | 43.5 | 34.6 | Mid |
| 23 | MCTS1 | 16.5 | 17.0 | Mid |
| 23 | MECP2 | 75.9 | 10.1 | Mid |
| 19 | MED25 | 18.7 | 88.7 | Mid |
| 13 | MED4 | 19.2 | 47.0 | Mid |
| 17 | MED9 | 16.2 | 13.7 | Mid |
| 16 | METTL9 | 57.9 | 48.7 | Mid |
| 1 | MFN2 | 33.3 | 173.4 | Mid |
| 1 | MFSD14A | 45.3 | 62.7 | Mid |
| 16 | MGRN1 | 66.2 | 39.6 | Mid |
| 22 | MIEF1 | 15.9 | 18.4 | Mid |
| 3 | MKRN2 | 26.7 | 54.7 | Mid |
| 2 | MMADHC | 18.2 | 139.0 | Mid |
| 22 | MORC2 | 41.6 | 31.2 | Mid |
| 15 | MORF4L1 | 25.0 | 277.2 | Mid |
| 2 | MPHOSPH10 | 19.8 | 26.5 | Mid |
| 5 | MRPL22 | 28.3 | 12.2 | Mid |
| 1 | MRPL37 | 18.2 | 58.3 | Mid |
| 21 | MRPL39 | 21.8 | 46.2 | Mid |
| 3 | MRPS25 | 16.8 | 66.6 | Mid |
| 2 | MRPS5 | 34.8 | 117.3 | Mid |
| 2 | MRPS9 | 61.9 | 33.8 | Mid |
| 3 | MSL2 | 46.9 | 28.2 | Mid |
| 6 | MTCH1 | 18.4 | 301.6 | Mid |
| 8 | MTDH | 86.1 | 47.1 | Mid |
| 6 | MTFR1L | 15.5 | 12.1 | Mid |
| 7 | MTPN | 50.6 | 158.3 | Mid |
| 12 | NAP1L1 | 40.1 | 76.2 | Mid |
| 11 | NAP1L4 | 48.0 | 104.0 | Mid |
| 19 | NAPA | 27.6 | 95.1 | Mid |
| 18 | NAPG | 26.9 | 49.5 | Mid |
| 18 | NARS | 21.3 | 129.6 | Mid |
| 11 | NAT10 | 41.4 | 26.7 | Mid |
| 9 | NCBP1 | 40.3 | 20.8 | Mid |
| 20 | NCOA5 | 29.0 | 59.5 | Mid |
| 5 | NDST1 | 50.1 | 43.9 | Mid |
| 9 | NELFB | 18.2 | 70.8 | Mid |
| 16 | NFATC2IP | 15.5 | 54.7 | Mid |
| 9 | NFX1 | 80.7 | 30.7 | Mid |
| 12 | NFYB | 21.2 | 62.4 | Mid |
| 3 | NMD3 | 30.7 | 26.0 | Mid |
| 17 | NMT1 | 47.7 | 60.0 | Mid |
| 4 | NOP14 | 25.5 | 39.5 | Mid |
| 19 | NOSIP | 25.1 | 61.0 | Mid |
| 17 | NPEPPS | 92.2 | 102.4 | Mid |
| 17 | NPLOC4 | 80.2 | 34.0 | Mid |
| 16 | NSMCE1 | 43.8 | 44.4 | Mid |
| 10 | NSMCE4A | 18.1 | 45.4 | Mid |
| 1 | NUCKS1 | 37.4 | 167.0 | Mid |
| 1 | NUDC | 25.2 | 130.0 | Mid |
| 16 | NUDT21 | 22.2 | 69.2 | Mid |
| 4 | NUDT9 | 36.9 | 30.4 | Mid |
| 17 | NUFIP2 | 38.3 | 41.0 | Mid |
| 11 | NUMA1 | 77.7 | 96.6 | Mid |
| 1 | NUP133 | 67.1 | 33.0 | Mid |
| 6 | NUP153 | 91.8 | 32.6 | Mid |
| 6 | NUS1 | 35.3 | 19.6 | Mid |
| 23 | OGT | 42.8 | 256.5 | Mid |
| 12 | OS9 | 27.6 | 238.4 | Mid |
| 11 | OSBP | 41.7 | 51.0 | Mid |
| 4 | OSTC | 17.2 | 101.5 | Mid |
| 4 | OTUD4 | 46.0 | 24.2 | Mid |
| 3 | OXSR1 | 90.0 | 29.6 | Mid |
| 17 | P4HB | 17.5 | 960.7 | Mid |
| 17 | PAFAH1B1 | 92.0 | 88.4 | Mid |
| 11 | PAFAH1B2 | 26.8 | 60.4 | Mid |
| 5 | PAIP1 | 30.8 | 42.1 | Mid |
| 1 | PARK7 | 23.6 | 273.0 | Mid |
| 1 | PARP1 | 47.4 | 59.2 | Mid |
| 11 | PATL1 | 32.3 | 43.0 | Mid |
| 12 | PCBP2 | 29.1 | 241.2 | Mid |
| 4 | PCGF3 | 64.9 | 71.2 | Mid |
| 6 | PCMT1 | 61.7 | 151.5 | Mid |
| 20 | PCMTD2 | 20.5 | 55.3 | Mid |
| 11 | PCSK7 | 27.0 | 34.6 | Mid |
| 2 | PCYOX1 | 23.1 | 75.4 | Mid |
| 3 | PCYT1A | 49.3 | 30.1 | Mid |
| 5 | PDCD6 | 43.4 | 60.4 | Mid |
| 15 | PDCD7 | 16.5 | 36.0 | Mid |
| 15 | PDIA3 | 26.2 | 328.0 | Mid |
| 2 | PDIA6 | 29.4 | 129.6 | Mid |
| 16 | PDPK1 | 27.2 | ~30.0 | Mid |
| 17 | PELP1 | 33.0 | 43.8 | Mid |
| 5 | PFDN1 | 58.1 | 93.5 | Mid |
| 1 | PFDN2 | 17.5 | 201.0 | Mid |
| 4 | PGRMC2 | 20.0 | 71.3 | Mid |
| 17 | PHF12 | 46.2 | 27.6 | Mid |
| 11 | PHRF1 | 35.7 | 46.3 | Mid |
| 19 | PIP5K1C | 70.3 | 80.9 | Mid |
| 9 | PLAA | 44.1 | 23.0 | Mid |
| 7 | PMPCB | 17.3 | 64.7 | Mid |
| 2 | PNO1 | 18.1 | 19.8 | Mid |
| 20 | POFUT1 | 30.8 | 25.4 | Mid |
| 13 | POLR1D | 45.6 | 27.6 | Mid |
| 1 | POLR3C | 18.3 | 23.7 | Mid |
| 12 | PPP1CC | 23.2 | 66.6 | Mid |
| 19 | PPP1R37 | 54.1 | 32.0 | Mid |
| 1 | PPP1R8 | 20.9 | 37.4 | Mid |
| 5 | PPP2CA | 29.8 | 99.6 | Mid |
| 8 | PPP2CB | 27.2 | 90.8 | Mid |
| 19 | PPP2R1A | 36.6 | 181.2 | Mid |
| 1 | PPP2R5A | 76.3 | 46.2 | Mid |
| 19 | PPP5C | 44.0 | 75.3 | Mid |
| 9 | PPP6C | 43.4 | 43.0 | Mid |
| 19 | PPP6R1 | 28.9 | 63.8 | Mid |
| 1 | PPT1 | 24.8 | 111.5 | Mid |
| 12 | PPTC7 | 48.8 | 32.3 | Mid |
| 1 | PRCC | 33.3 | 62.6 | Mid |
| 12 | PRDM4 | 28.3 | 29.6 | Mid |
| 5 | PRKAA1 | 38.8 | 58.3 | Mid |
| 17 | PRKAR1A | 21.7 | 123.3 | Mid |
| 3 | PRKAR2A | 97.2 | 25.5 | Mid |
| 19 | PRKCSH | 15.5 | 240.7 | Mid |
| 2 | PRKRA | 19.8 | 50.8 | Mid |
| 10 | PRPF18 | 43.9 | 25.2 | Mid |
| 11 | PRPF19 | 16.0 | 189.8 | Mid |
| 23 | PRPS1 | 22.6 | 44.4 | Mid |
| 17 | PRPSAP1 | 43.3 | 30.7 | Mid |
| 7 | PSMA2 | 15.3 | 41.3 | Mid |
| 1 | PSMA5 | 27.4 | 20.9 | Mid |
| 14 | PSMA6 | 25.1 | 110.3 | Mid |
| 6 | PSMB1 | 18.2 | 165.7 | Mid |
| 1 | PSMB2 | 42.3 | 36.2 | Mid |
| 9 | PSMB7 | 62.0 | 141.3 | Mid |
| 14 | PSMC1 | 16.1 | 172.2 | Mid |
| 17 | PSMD11 | 38.9 | 70.6 | Mid |
| 17 | PSMD12 | 26.1 | 24.8 | Mid |
| 17 | PSMD3 | 17.2 | 139.4 | Mid |
| 9 | PSMD5 | 27.0 | 23.9 | Mid |
| 18 | PSMG2 | 22.8 | 57.5 | Mid |
| 12 | PTPN11 | 91.2 | 68.9 | Mid |
| 2 | PUM2 | 78.8 | 80.7 | Mid |
| 12 | PWP1 | 26.7 | 51.8 | Mid |
| 21 | PWP2 | 23.9 | 2.7 | Mid |
| 3 | QRICH1 | 64.4 | 68.4 | Mid |
| 15 | RAB11A | 22.5 | 112.0 | Mid |
| 9 | RAB14 | 24.0 | 93.3 | Mid |
| 12 | RAB35 | 21.7 | 55.5 | Mid |
| 3 | RAB5A | 38.1 | 49.2 | Mid |
| 11 | RAB6A | 85.5 | 130.6 | Mid |
| 3 | RAB7A | 88.7 | 312.3 | Mid |
| 23 | RAB9A | 20.1 | 45.4 | Mid |
| 9 | RABL6 | 33.5 | 78.9 | Mid |
| 7 | RAC1 | 29.5 | 372.6 | Mid |
| 8 | RAD21 | 28.9 | 99.4 | Mid |
| 9 | RAD23B | 49.0 | 100.0 | Mid |
| 7 | RALA | 84.6 | 24.4 | Mid |
| 18 | RALBP1 | 62.6 | 34.6 | Mid |
| 20 | RALY | 89.5 | 65.6 | Mid |
| 16 | RANBP10 | 83.6 | 31.8 | Mid |
| 2 | RANBP2 | 66.3 | 37.7 | Mid |
| 6 | RANBP9 | 90.1 | 53.9 | Mid |
| 22 | RANGAP1 | 40.6 | 100.8 | Mid |
| 6 | RARS2 | 75.6 | 34.8 | Mid |
| 20 | RBCK1 | 22.9 | 116.3 | Mid |
| 10 | RBM17 | 28.5 | 70.9 | Mid |
| 14 | RBM25 | 62.9 | 29.5 | Mid |
| 11 | RBM4 | 29.8 | 46.9 | Mid |
| 22 | RBX1 | 21.7 | 114.3 | Mid |
| 9 | RC3H2 | 55.8 | 14.3 | Mid |
| 7 | RHEB | 53.9 | 84.1 | Mid |
| 3 | RNF168 | 35.0 | 20.0 | Mid |
| 9 | RNF20 | 29.5 | 36.3 | Mid |
| 17 | RPA1 | 69.6 | 41.3 | Mid |
| 1 | RPA2 | 23.2 | 55.6 | Mid |
| 3 | RPN1 | 30.9 | 142.3 | Mid |
| 20 | RPN2 | 62.6 | 230.7 | Mid |
| 16 | RRN3 | 34.3 | 38.6 | Mid |
| 15 | RSL24D1 | 15.7 | 103.2 | Mid |
| 12 | RSRC2 | 22.4 | 55.3 | Mid |
| 22 | RTCB | 24.7 | 117.1 | Mid |
| 15 | RTF1 | 66.5 | 39.8 | Mid |
| 3 | RUVBL1 | 42.9 | 17.5 | Mid |
| 10 | SAR1A | 20.3 | 45.1 | Mid |
| 12 | SARNP | 65.3 | 125.4 | Mid |
| 1 | SARS | 24.3 | 107.7 | Mid |
| 11 | SART1 | 18.5 | 58.3 | Mid |
| 12 | SART3 | 39.2 | 44.6 | Mid |
| 14 | SAV1 | 34.8 | 31.8 | Mid |
| 19 | SCAF1 | 16.5 | 54.2 | Mid |
| 12 | SCYL2 | 72.4 | 24.9 | Mid |
| 1 | SDF4 | 15.2 | 196.7 | Mid |
| 11 | SDHAF2 | 16.6 | 32.2 | Mid |
| 3 | SEC13 | 20.2 | 96.6 | Mid |
| 4 | SEC31A | 72.6 | 98.8 | Mid |
| 3 | SEC62 | 31.6 | 61.7 | Mid |
| 3 | SENP2 | 44.9 | 23.6 | Mid |
| 3 | SENP5 | 66.9 | 17.3 | Mid |
| 10 | SEPHS1 | 30.9 | 27.6 | Mid |
| 1 | SERBP1 | 22.6 | 79.9 | Mid |
| 3 | SETD5 | 80.4 | 58.7 | Mid |
| 1 | SF3A3 | 33.1 | 43.8 | Mid |
| 2 | SF3B1 | 43.1 | 173.0 | Mid |
| 11 | SF3B2 | 16.6 | 134.7 | Mid |
| 19 | SGTA | 28.6 | 78.3 | Mid |
| 19 | SH3GL1 | 40.2 | 155.1 | Mid |
| 5 | SKP1 | 20.6 | 72.2 | Mid |
| 5 | SLC30A5 | 37.1 | 25.2 | Mid |
| 4 | SLC30A9 | 97.0 | 35.9 | Mid |
| 6 | SLC35A1 | 39.4 | 51.0 | Mid |
| 19 | SLC35E1 | 22.6 | 23.0 | Mid |
| 18 | SLC39A6 | 20.9 | 63.5 | Mid |
| 2 | SLC4A1AP | 31.5 | 27.7 | Mid |
| 15 | SLTM | 54.6 | 76.7 | Mid |
| 5 | SLU7 | 17.5 | 39.4 | Mid |
| 18 | SMAD2 | 98.1 | 8.0 | Mid |
| 23 | SMARCA5 | 77.0 | 54.6 | Mid |
| 22 | SMARCB1 | 47.6 | 98.7 | Mid |
| 23 | SMC1A | 48.6 | 28.1 | Mid |
| 9 | SMU1 | 34.9 | 28.8 | Mid |
| 22 | SNAP29 | 32.2 | 23.9 | Mid |
| 22 | SNRPD3 | 16.9 | 91.2 | Mid |
| 15 | SNX1 | 48.4 | 57.7 | Mid |
| 11 | SNX19 | 40.6 | 36.7 | Mid |
| 1 | SNX27 | 86.9 | 19.6 | Mid |
| 6 | SNX3 | 49.8 | 261.8 | Mid |
| 21 | SON | 34.5 | 23.8 | Mid |
| 12 | SP1 | 36.3 | 38.6 | Mid |
| 17 | SP2 | 32.8 | 31.5 | Mid |
| 12 | SPRYD3 | 15.1 | 208.2 | Mid |
| 14 | SPTSSA | 29.3 | 67.7 | Mid |
| 22 | SREBF2 | 73.3 | 109.7 | Mid |
| 3 | SRPRB | 37.5 | 61.0 | Mid |
| 1 | SRRM1 | 30.2 | 106.6 | Mid |
| 1 | SRSF11 | 46.3 | 138.8 | Mid |
| 1 | SRSF4 | 34.4 | 145.8 | Mid |
| 3 | SSR3 | 15.1 | 77.5 | Mid |
| 1 | SSU72 | 33.2 | 60.7 | Mid |
| 22 | ST13 | 32.1 | 182.5 | Mid |
| 17 | STAT3 | 75.2 | 172.9 | Mid |
| 20 | STAU1 | 75.0 | 76.0 | Mid |
| 11 | STIP1 | 18.4 | 94.3 | Mid |
| 19 | STK11 | 22.6 | 33.9 | Mid |
| 6 | STK38 | 53.6 | 54.3 | Mid |
| 12 | STRAP | 21.1 | 143.4 | Mid |
| 19 | STRN4 | 27.0 | 58.2 | Mid |
| 11 | STX5 | 25.2 | 75.0 | Mid |
| 5 | SUB1 | 18.6 | 62.9 | Mid |
| 13 | SUGT1 | 35.6 | 18.1 | Mid |
| 2 | SUMO1 | 32.4 | 83.5 | Mid |
| 17 | SUPT6H | 40.0 | 76.2 | Mid |
| 6 | SYNCRIP | 29.4 | 46.7 | Mid |
| 22 | TAB1 | 32.1 | 45.6 | Mid |
| 7 | TAX1BP1 | 89.7 | 33.5 | Mid |
| 20 | TBC1D20 | 27.1 | 45.4 | Mid |
| 5 | TBCA | 85.2 | 80.4 | Mid |
| 12 | TBK1 | 50.1 | 27.1 | Mid |
| 16 | TCF25 | 37.8 | 84.4 | Mid |
| 3 | THOC7 | 30.1 | 80.2 | Mid |
| 1 | THRAP3 | 80.9 | 75.5 | Mid |
| 1 | TIMM17A | 15.2 | 30.3 | Mid |
| 10 | TM9SF3 | 69.0 | 74.8 | Mid |
| 12 | TMBIM6 | 23.1 | 314.0 | Mid |
| 1 | TMCO1 | 44.6 | 34.9 | Mid |
| 14 | TMED10 | 45.2 | 165.5 | Mid |
| 2 | TMEM127 | 15.8 | 52.3 | Mid |
| 4 | TMEM165 | 30.3 | 36.6 | Mid |
| 1 | TMEM183A | 16.7 | 40.7 | Mid |
| 7 | TMEM248 | 37.3 | 76.3 | Mid |
| 6 | TMEM30A | 32.0 | 131.2 | Mid |
| 1 | TMEM50A | 24.0 | 102.6 | Mid |
| 18 | TMX3 | 41.4 | 28.2 | Mid |
| 20 | TMX4 | 38.7 | 57.2 | Mid |
| 10 | TNKS2 | 67.1 | 41.8 | Mid |
| 11 | TOLLIP | 35.3 | 67.6 | Mid |
| 1 | TOMM20 | 19.6 | 191.9 | Mid |
| 13 | TPP2 | 82.2 | 22.3 | Mid |
| 8 | TRAM1 | 35.2 | 127.3 | Mid |
| 15 | TRIP4 | 67.5 | 20.4 | Mid |
| 20 | TRPC4AP | 90.4 | 98.4 | Mid |
| 5 | TTC1 | 56.4 | 68.6 | Mid |
| 5 | TTC37 | 91.1 | 45.0 | Mid |
| 11 | TUT1 | 16.6 | 27.5 | Mid |
| 1 | TXNDC12 | 36.0 | 33.7 | Mid |
| 2 | TXNDC9 | 17.4 | 17.7 | Mid |
| 18 | TXNL1 | 36.2 | 18.1 | Mid |
| 19 | U2AF2 | 20.7 | 156.8 | Mid |
| 23 | UBA1 | 24.3 | 137.8 | Mid |
| 19 | UBA2 | 41.5 | 89.5 | Mid |
| 9 | UBAC1 | 28.4 | 67.8 | Mid |
| 1 | UBAP2L | 50.0 | 55.6 | Mid |
| 5 | UBE2D2 | 67.3 | 80.6 | Mid |
| 2 | UBE2F | 74.0 | 27.7 | Mid |
| 17 | UBE2G1 | 97.5 | 36.4 | Mid |
| 16 | UBE2I | 17.9 | 67.0 | Mid |
| 6 | UBE2J1 | 26.3 | 34.8 | Mid |
| 4 | UBE2K | 84.8 | 34.1 | Mid |
| 17 | UBE2Z | 20.7 | 69.0 | Mid |
| 16 | UBFD1 | 16.9 | 28.2 | Mid |
| 15 | UBL7 | 15.2 | 60.9 | Mid |
| 3 | UBP1 | 52.1 | 45.0 | Mid |
| 9 | UBQLN1 | 48.3 | 79.3 | Mid |
| 1 | UBQLN4 | 18.4 | 44.5 | Mid |
| 6 | UFL1 | 35.5 | 28.1 | Mid |
| 2 | UGP2 | 49.7 | 210.2 | Mid |
| 16 | USB1 | 20.3 | 26.3 | Mid |
| 18 | USP14 | 55.3 | 41.5 | Mid |
| 17 | USP22 | 43.5 | 70.6 | Mid |
| 2 | USP39 | 33.2 | 34.2 | Mid |
| 3 | USP4 | 63.0 | 37.2 | Mid |
| 17 | UTP18 | 37.4 | 50.5 | Mid |
| 9 | VCP | 16.7 | 156.2 | Mid |
| 5 | VDAC1 | 33.3 | 187.3 | Mid |
| 17 | VEZF1 | 16.7 | 44.4 | Mid |
| 20 | VPS16 | 26.0 | 51.8 | Mid |
| 10 | VPS26A | 48.7 | 53.5 | Mid |
| 11 | VPS26B | 23.1 | 55.0 | Mid |
| 13 | VPS36 | 38.0 | 53.5 | Mid |
| 11 | VPS37C | 31.2 | 32.2 | Mid |
| 11 | VPS51 | 15.8 | 99.4 | Mid |
| 6 | VPS52 | 21.6 | 58.5 | Mid |
| 14 | VTI1B | 23.7 | 52.8 | Mid |
| 10 | WAC | 89.6 | 63.1 | Mid |
| 7 | WASL | 67.1 | 51.6 | Mid |
| 13 | WBP4 | 22.4 | 26.0 | Mid |
| 17 | WDR45B | 34.0 | 98.1 | Mid |
| 3 | WDR82 | 24.2 | 88.8 | Mid |
| 15 | WHAMM | 25.6 | 18.1 | Mid |
| 7 | WIPI2 | 43.7 | 32.2 | Mid |
| 6 | WRNIP1 | 20.3 | 38.3 | Mid |
| 12 | XPOT | 44.3 | 32.0 | Mid |
| 1 | YARS | 42.8 | 34.6 | Mid |
| 10 | YME1L1 | 44.3 | 44.0 | Mid |
| 4 | YTHDC1 | 39.7 | 46.5 | Mid |
| 20 | YTHDF1 | 20.8 | 57.7 | Mid |
| 20 | YWHAB | 22.8 | 217.4 | Mid |
| 17 | YWHAE | 55.7 | 220.8 | Mid |
| 2 | YWHAQ | 47.0 | 244.4 | Mid |
| 14 | YY1 | 40.3 | 38.5 | Mid |
| 1 | ZBTB17 | 34.3 | 32.2 | Mid |
| 1 | ZC3H11A | 58.5 | 2.0 | Mid |
| 2 | ZC3H15 | 23.2 | 54.3 | Mid |
| 16 | ZC3H18 | 61.6 | 29.8 | Mid |
| 10 | ZDHHC6 | 16.6 | 41.3 | Mid |
| 16 | ZDHHC7 | 37.1 | 73.4 | Mid |
| 19 | ZNF146 | 24.2 | 73.4 | Mid |
| 7 | ZNF777 | 29.6 | 23.3 | Mid |
| 11 | ZFP91 | 42.4 | 62.8 | Mid |
| 14 | ZFYVE21 | 17.9 | 111.2 | Mid |
| 1 | ZMPSTE24 | 36.1 | 43.6 | Mid |
| 10 | ZRANB1 | 45.3 | 49.5 | Mid |
| 1 | ZRANB2 | 18.0 | 90.0 | Mid |
| 23 | ZRSR2 | 32.8 | 38.0 | Mid |

| 6 | C6orf120 | 2.7 | 24.8 | Short |
| --- | --- | --- | --- | --- |
| 14 | C14orf119 | 5.0 | 42.7 | Short |
| 11 | C11orf98 | 2.4 | 23.7 | Short |
| 1 | C1orf43 | 13.9 | 147.0 | Short |
| 1 | C1orf174 | 11.1 | 29.6 | Short |
| 2 | AAMP | 6.1 | 29.6 | Short |
| 17 | AARSD1 | 13.9 | 63.9 | Short |
| 2 | ACTR1B | 8.1 | 172.8 | Short |
| 20 | ADRM1 | 6.8 | 163.1 | Short |
| 14 | AHSA1 | 11.4 | 143.8 | Short |
| 11 | AIP | 8.1 | 158.4 | Short |
| 1 | AKIRIN1 | 14.8 | 150.2 | Short |
| 9 | ANAPC2 | 13.8 | 83.1 | Short |
| 17 | ANKRD40 | 14.7 | 194.8 | Short |
| 3 | AP2M1 | 9.2 | 343.1 | Short |
| 1 | APEH | 9.6 | 88.5 | Short |
| 11 | APH1A | 3.6 | 157.7 | Short |
| 23 | ARAF | 10.7 | 73.0 | Short |
| 11 | ARFGAP2 | 12.6 | 120.0 | Short |
| 12 | ARL1 | 14.7 | 69.2 | Short |
| 16 | ARL6IP1 | 9.9 | 434.0 | Short |
| 23 | ARMCX3 | 5.1 | 81.6 | Short |
| 2 | ASNSD1 | 9.4 | 54.7 | Short |
| 6 | ATF6B | 12.9 | 138.2 | Short |
| 12 | ATG101 | 7.5 | 32.9 | Short |
| 21 | ATP5PF | 11.2 | 296.9 | Short |
| 5 | B4GALT7 | 10.2 | 45.9 | Short |
| 11 | BAD | 14.8 | 66.7 | Short |
| 9 | BAG1 | 12.2 | 62.2 | Short |
| 6 | BAG6 | 13.7 | 115.1 | Short |
| 11 | BANF1 | 1.9 | 155.3 | Short |
| 3 | BAP1 | 9.0 | 119.7 | Short |
| 9 | C9orf78 | 8.0 | 31.0 | Short |
| 16 | BCKDK | 6.5 | 58.5 | Short |
| 11 | BET1L | 4.5 | 63.5 | Short |
| 10 | BLOC1S2 | 13.4 | 44.7 | Short |
| 10 | BMI1 | 10.4 | 53.0 | Short |
| 5 | BOD1 | 9.5 | 90.9 | Short |
| 10 | BORCS7 | 10.7 | 64.8 | Short |
| 3 | BRK1 | 11.5 | 328.4 | Short |
| 11 | BRMS1 | 7.8 | 63.3 | Short |
| 5 | BTF3 | 7.1 | 1319.0 | Short |
| 7 | BUD31 | 11.0 | 54.7 | Short |
| 2 | BZW1 | 9.9 | 40.9 | Short |
| 14 | CALM1 | 11.2 | 1259.0 | Short |
| 19 | CALR | 5.9 | 1314.0 | Short |
| 19 | CCDC124 | 11.0 | 125.9 | Short |
| 19 | CCDC97 | 14.7 | 156.1 | Short |
| 16 | CD2BP2 | 4.5 | 65.9 | Short |
| 9 | CDC26 | 8.6 | 15.1 | Short |
| 19 | CDC34 | 10.3 | 125.4 | Short |
| 19 | CDC37 | 12.4 | 248.8 | Short |
| 12 | CDK4 | 4.6 | 30.7 | Short |
| 12 | CDKN1B | 5.0 | 193.4 | Short |
| 11 | CFL1 | 4.7 | 365.0 | Short |
| 10 | CHCHD1 | 1.6 | 37.7 | Short |
| 19 | CHMP2A | 3.5 | 134.8 | Short |
| 17 | CHMP6 | 8.3 | 27.4 | Short |
| 1 | CHTOP | 12.2 | 57.2 | Short |
| 19 | CLPP | 8.7 | 77.4 | Short |
| 3 | CNBP | 14.4 | 844.9 | Short |
| 2 | CNPPD1 | 5.9 | 107.7 | Short |
| 17 | COA3 | 1.1 | 196.4 | Short |
| 2 | COA5 | 9.2 | 56.2 | Short |
| 1 | COA6 | 10.6 | 40.2 | Short |
| 17 | COASY | 4.1 | 84.3 | Short |
| 7 | COPS6 | 3.2 | 170.3 | Short |
| 3 | COX17 | 8.1 | 66.0 | Short |
| 19 | CSNK1G2 | 2.0 | 76.5 | Short |
| 6 | CSNK2B | 4.0 | 426.7 | Short |
| 17 | CTDNEP1 | 8.5 | 266.6 | Short |
| 10 | CUEDC2 | 9.3 | 185.3 | Short |
| 6 | CUTA | 1.9 | 378.7 | Short |
| 19 | DCAF15 | 9.0 | 29.7 | Short |
| 9 | DCTN3 | 7.0 | 49.7 | Short |
| 16 | DCTPP1 | 6.4 | 36.5 | Short |
| 12 | DDX51 | 7.7 | 48.2 | Short |
| 19 | DHPS | 6.2 | 146.6 | Short |
| 9 | DNAJA1 | 14.6 | 137.8 | Short |
| 11 | DNAJC4 | 4.0 | 117.2 | Short |
| 10 | DNAJC9 | 5.3 | 29.3 | Short |
| 9 | DNLZ | 4.3 | 9.3 | Short |
| 16 | E2F4 | 6.8 | 99.1 | Short |
| 16 | E4F1 | 12.2 | 69.3 | Short |
| 1 | EBNA1BP2 | 8.1 | 34.1 | Short |
| 19 | EGLN2 | 9.2 | 67.6 | Short |
| 3 | EIF1B | 2.7 | 248.6 | Short |
| 12 | EIF2B1 | 13.3 | 56.6 | Short |
| 19 | EIF3G | 4.9 | 321.2 | Short |
| 1 | EIF3I | 9.2 | 200.1 | Short |
| 17 | EIF4A1 | 6.2 | 468.1 | Short |
| 17 | EIF4A3 | 12.7 | 92.8 | Short |
| 11 | EIF4G2 | 7.0 | 388.5 | Short |
| 17 | EIF5A | 4.9 | 405.8 | Short |
| 23 | ELK1 | 15.0 | 79.9 | Short |
| 19 | ELOF1 | 6.2 | 33.8 | Short |
| 15 | EMC4 | 5.1 | 86.0 | Short |
| 17 | EMC6 | 0.9 | 100.4 | Short |
| 23 | EMD | 2.3 | 236.6 | Short |
| 1 | ENSA | 8.4 | 109.0 | Short |
| 17 | ERAL1 | 6.1 | 88.6 | Short |
| 12 | ERP29 | 10.0 | 170.4 | Short |
| 8 | EXOSC4 | 2.0 | 25.1 | Short |
| 9 | FAM122A | 5.5 | 27.6 | Short |
| 23 | FAM50A | 6.5 | 128.6 | Short |
| 19 | FBL | 11.9 | 380.0 | Short |
| 9 | FBXW5 | 4.3 | 254.0 | Short |
| 11 | FIBP | 4.6 | 98.2 | Short |
| 7 | FIS1 | 5.6 | 231.1 | Short |
| 19 | FIZ1 | 8.1 | 13.8 | Short |
| 19 | FKBP8 | 11.8 | 501.7 | Short |
| 17 | FTSJ3 | 8.2 | 54.4 | Short |
| 17 | G6PC3 | 5.6 | 110.3 | Short |
| 16 | GABARAPL2 | 11.5 | 156.0 | Short |
| 7 | GATAD1 | 12.6 | 82.8 | Short |
| 20 | GID8 | 10.3 | 52.1 | Short |
| 16 | GNPTG | 12.2 | 102.4 | Short |
| 23 | GPKOW | 10.3 | 42.8 | Short |
| 1 | GPN2 | 14.2 | 27.7 | Short |
| 19 | GPX4 | 2.8 | 699.6 | Short |
| 19 | GSK3A | 12.4 | 79.9 | Short |
| 10 | GSTO1 | 12.6 | 133.3 | Short |
| 19 | GTF2F1 | 13.6 | 147.3 | Short |
| 13 | GTF3A | 11.2 | 77.5 | Short |
| 6 | GTF3C6 | 9.2 | 76.0 | Short |
| 1 | GUK1 | 8.9 | 151.6 | Short |
| 7 | H2AFV | 13.8 | 152.2 | Short |
| 17 | H3F3B | 6.9 | 1232.0 | Short |
| 1 | HAX1 | 3.4 | 179.3 | Short |
| 5 | HINT1 | 6.2 | 217.5 | Short |
| 12 | HNRNPA1 | 6.9 | 1092.5 | Short |
| 5 | HNRNPAB | 6.6 | 159.7 | Short |
| 4 | HNRNPDL | 7.7 | 308.8 | Short |
| 1 | HNRNPU | 14.3 | 129.3 | Short |
| 11 | HNRNPUL2 | 14.8 | 172.7 | Short |
| 16 | HSBP1 | 11.8 | 32.8 | Short |
| 6 | HSP90AB1 | 7.7 | 1823.0 | Short |
| 2 | HSPE1 | 3.5 | 107.2 | Short |
| 23 | HTATSF1 | 14.8 | 1231.6 | Short |
| 1 | ICMT | 14.8 | 54.5 | Short |
| 5 | IK | 14.7 | 144.4 | Short |
| 1 | ILF2 | 9.2 | 125.7 | Short |
| 19 | ILF3 | 6.5 | 112.2 | Short |
| 14 | ISCA2 | 1.9 | 18.8 | Short |
| 12 | ISCU | 6.9 | 227.3 | Short |
| 3 | JAGN1 | 3.8 | 40.8 | Short |
| 22 | JOSD1 | 14.9 | 82.4 | Short |
| 1 | JTB | 3.7 | 324.2 | Short |
| 16 | KAT8 | 13.7 | 102.1 | Short |
| 19 | KDELR1 | 9.0 | 298.2 | Short |
| 19 | KHSRP | 11.7 | 130.4 | Short |
| 19 | KXD1 | 11.6 | 80.3 | Short |
| 11 | LAMTOR1 | 6.1 | 111.1 | Short |
| 1 | LAMTOR5 | 6.6 | 147.9 | Short |
| 10 | LDB1 | 12.9 | 180.2 | Short |
| 6 | LEMD2 | 7.9 | 71.2 | Short |
| 19 | LENG1 | 4.1 | 28.2 | Short |
| 19 | LENG8 | 13.1 | ~300.0 | Short |
| 22 | LMF2 | 4.8 | 144.0 | Short |
| 8 | LSM1 | 13.4 | 40.4 | Short |
| 20 | LSM14B | 12.9 | 58.2 | Short |
| 1 | MAGOH | 11.7 | 33.6 | Short |
| 16 | MAZ | 4.6 | 58.7 | Short |
| 18 | MBD1 | 12.9 | 48.6 | Short |
| 12 | MCRS1 | 8.1 | 77.4 | Short |
| 6 | MEA1 | 1.7 | 145.0 | Short |
| 5 | MED10 | 6.6 | 62.4 | Short |
| 11 | MED19 | 8.5 | 24.0 | Short |
| 12 | MED21 | 8.2 | 31.3 | Short |
| 4 | MED28 | 9.9 | 38.1 | Short |
| 7 | MEPCE | 4.5 | 80.1 | Short |
| 12 | MLEC | 14.7 | 108.6 | Short |
| 12 | MLF2 | 4.9 | 223.0 | Short |
| 23 | MORF4L2 | 12.7 | 402.5 | Short |
| 11 | MRPL17 | 3.0 | 44.6 | Short |
| 6 | MRPL18 | 8.0 | 78.9 | Short |
| 1 | MRPL24 | 3.8 | 66.6 | Short |
| 16 | MRPL28 | 3.2 | 90.1 | Short |
| 5 | MRPL36 | 1.5 | 23.1 | Short |
| 17 | MRPL38 | 6.5 | 57.2 | Short |
| 9 | MRPL41 | 0.7 | 202.4 | Short |
| 10 | MRPL43 | 6.1 | 48.1 | Short |
| 2 | MRPL44 | 10.3 | 22.9 | Short |
| 11 | MRPL49 | 5.1 | 72.3 | Short |
| 12 | MRPL51 | 1.2 | 133.6 | Short |
| 13 | MRPL57 | 2.4 | 43.2 | Short |
| 1 | MRPL9 | 3.9 | 67.5 | Short |
| 6 | MRPS10 | 11.1 | 50.6 | Short |
| 1 | MRPS14 | 10.5 | 32.3 | Short |
| 10 | MRPS16 | 3.9 | 55.4 | Short |
| 7 | MRPS17 | 3.4 | 22.0 | Short |
| 9 | MRPS2 | 4.0 | 39.6 | Short |
| 5 | MRPS30 | 6.6 | 13.2 | Short |
| 1 | MRTO4 | 8.6 | 25.8 | Short |
| 17 | MSL1 | 14.3 | 115.6 | Short |
| 19 | MYDGF | 12.8 | 142.6 | Short |
| 12 | NACA | 13.1 | 186.3 | Short |
| 3 | NCBP2 | 7.2 | 60.2 | Short |
| 2 | NCL | 9.7 | 233.8 | Short |
| 10 | NDUFB8 | 6.1 | 305.0 | Short |
| 14 | NEDD8 | 5.8 | 73.2 | Short |
| 14 | NGDN | 8.5 | 27.9 | Short |
| 15 | NGRN | 6.6 | 181.7 | Short |
| 2 | NIFK | 9.9 | 31.8 | Short |
| 16 | NOB1 | 13.1 | 89.0 | Short |
| 6 | NOL7 | 5.6 | 91.6 | Short |
| 14 | NOP9 | 5.3 | 18.2 | Short |
| 2 | NRBP1 | 13.9 | 108.2 | Short |
| 5 | NSA2 | 9.9 | 206.1 | Short |
| 16 | NUDT16L1 | 2.2 | 51.3 | Short |
| 11 | NXF1 | 13.4 | 142.3 | Short |
| 2 | OST4 | 1.2 | 316.3 | Short |
| 11 | OTUB1 | 12.6 | 108.3 | Short |
| 12 | PA2G4 | 9.6 | 102.9 | Short |
| 14 | PABPN1 | 6.0 | 288.4 | Short |
| 19 | PAF1 | 5.6 | 79.6 | Short |
| 23 | PBDC1 | 5.3 | 46.3 | Short |
| 6 | PBX2 | 5.5 | 119.5 | Short |
| 2 | PCBP1 | 1.8 | 297.8 | Short |
| 20 | PCIF1 | 13.3 | 79.9 | Short |
| 7 | PDAP1 | 14.0 | 108.1 | Short |
| 23 | PDZD11 | 3.6 | 59.2 | Short |
| 12 | PGAM5 | 11.9 | 19.9 | Short |
| 22 | PHF5A | 9.0 | 40.3 | Short |
| 14 | PIGH | 11.0 | 28.5 | Short |
| 19 | PIN1 | 14.5 | 132.6 | Short |
| 1 | PITHD1 | 9.9 | 137.3 | Short |
| 9 | PMPCA | 13.1 | 50.3 | Short |
| 1 | PNRC2 | 3.7 | 121.6 | Short |
| 7 | POLD2 | 8.9 | 68.7 | Short |
| 9 | POLE3 | 3.5 | 81.3 | Short |
| 16 | POLR2C | 9.4 | 82.5 | Short |
| 7 | POLR2J | 5.8 | 48.8 | Short |
| 8 | POLR2K | 3.4 | 57.1 | Short |
| 7 | POP7 | 1.5 | 66.6 | Short |
| 15 | PPIB | 7.3 | 567.0 | Short |
| 4 | PPID | 14.3 | 41.2 | Short |
| 6 | PPP1R11 | 3.2 | 56.0 | Short |
| 1 | PPP1R15B | 8.5 | 42.3 | Short |
| 11 | PRDX5 | 3.7 | 560.3 | Short |
| 2 | PREB | 3.9 | 107.2 | Short |
| 1 | PRPF38A | 13.8 | 28.6 | Short |
| 20 | PSMA7 | 6.7 | 211.7 | Short |
| 1 | PSMB4 | 2.4 | 410.5 | Short |
| 17 | PSMB6 | 2.4 | 228.8 | Short |
| 11 | PSMC3 | 7.7 | 199.9 | Short |
| 17 | PSMC5 | 4.6 | 232.3 | Short |
| 23 | PSMD10 | 7.4 | 82.5 | Short |
| 1 | PSMD4 | 12.8 | 212.3 | Short |
| 3 | PSMD6 | 13.1 | 79.9 | Short |
| 16 | PSMD7 | 9.5 | 90.1 | Short |
| 19 | PSMD8 | 9.3 | 119.7 | Short |
| 17 | PSME3 | 10.6 | 46.8 | Short |
| 7 | PSMG3 | 2.7 | 40.8 | Short |
| 17 | PTRH2 | 10.2 | 5.5 | Short |
| 8 | PUF60 | 13.0 | 112.8 | Short |
| 7 | PURB | 9.1 | 51.2 | Short |
| 8 | R3HCC1 | 8.2 | 46.5 | Short |
| 19 | RAB11B | 14.1 | 257.6 | Short |
| 11 | RAB1B | 8.9 | 235.7 | Short |
| 1 | RABGGTB | 8.9 | 62.8 | Short |
| 19 | RAD23A | 7.8 | 210.3 | Short |
| 22 | RANBP1 | 9.7 | 49.0 | Short |
| 11 | RBM14 | 13.3 | 64.8 | Short |
| 19 | RBM42 | 8.6 | 130.4 | Short |
| 1 | RBM8A | 6.0 | 42.4 | Short |
| 23 | RBMX2 | 11.4 | 19.8 | Short |
| 1 | RER1 | 13.7 | 80.3 | Short |
| 9 | RGP1 | 4.0 | 18.4 | Short |
| 7 | RHBDD2 | 9.9 | 377.5 | Short |
| 16 | RHOT2 | 6.1 | 231.9 | Short |
| 6 | RING1 | 4.2 | 161.9 | Short |
| 23 | RNF113A | 1.3 | 47.0 | Short |
| 8 | RNF139 | 13.9 | 42.1 | Short |
| 17 | RNF167 | 4.9 | 152.4 | Short |
| 2 | RNF181 | 2.0 | 103.3 | Short |
| 16 | RNPS1 | 14.8 | 97.3 | Short |
| 3 | RPL15 | 3.7 | 418.2 | Short |
| 1 | RPL22 | 14.6 | 502.2 | Short |
| 3 | RPL35A | 5.7 | 539.7 | Short |
| 15 | RPL4 | 5.5 | 2217.0 | Short |
| 6 | RPL7L1 | 7.1 | 50.2 | Short |
| 22 | RPS19BP1 | 3.8 | 156.0 | Short |
| 9 | RRAGA | 1.7 | 174.1 | Short |
| 6 | RRP36 | 8.0 | 36.8 | Short |
| 22 | RRP7A | 11.5 | 26.3 | Short |
| 3 | RRP9 | 8.5 | 32.2 | Short |
| 13 | SAP18 | 8.6 | 191.2 | Short |
| 7 | SBDS | 7.9 | 563.0 | Short |
| 11 | SCYL1 | 13.6 | 105.5 | Short |
| 9 | SEC61B | 8.3 | 144.4 | Short |
| 3 | SERP1 | 5.2 | 87.5 | Short |
| 11 | SF1 | 13.9 | 252.7 | Short |
| 19 | SF3A2 | 11.9 | 212.2 | Short |
| 1 | SF3B4 | 4.9 | 88.0 | Short |
| 6 | SF3B5 | 0.7 | 224.1 | Short |
| 2 | SF3B6 | 8.7 | 148.9 | Short |
| 1 | SFPQ | 9.5 | 224.6 | Short |
| 16 | SH2B1 | 10.5 | 63.6 | Short |
| 18 | SHARPIN | 5.6 | 69.0 | Short |
| 17 | SLC25A11 | 3.0 | 141.5 | Short |
| 10 | SLC25A28 | 10.0 | 90.5 | Short |
| 12 | SLC25A3 | 8.4 | 169.3 | Short |
| 23 | SLC25A5 | 3.1 | 506.2 | Short |
| 5 | SLC35A4 | 4.3 | 69.2 | Short |
| 14 | SLIRP | 9.5 | 38.1 | Short |
| 22 | SMDT1 | 4.6 | 112.6 | Short |
| 1 | SMIM12 | 9.5 | 16.2 | Short |
| 11 | SMPD1 | 4.6 | 63.0 | Short |
| 2 | SMYD5 | 13.0 | 19.9 | Short |
| 1 | SNAPIN | 3.2 | 39.5 | Short |
| 17 | SNF8 | 14.7 | 35.4 | Short |
| 12 | SNRNP35 | 8.3 | 18.2 | Short |
| 19 | SNRNP70 | 6.5 | 638.5 | Short |
| 15 | SNRPA | 13.7 | 36.4 | Short |
| 20 | SNRPB | 9.2 | 170.5 | Short |
| 23 | SNX12 | 9.2 | 56.9 | Short |
| 21 | SOD1 | 9.3 | 347.7 | Short |
| 17 | SPAG7 | 8.6 | 68.7 | Short |
| 16 | SPG7 | 11.8 | 47.7 | Short |
| 1 | SRM | 5.4 | 152.0 | Short |
| 15 | SRP14 | 3.5 | 332.0 | Short |
| 1 | SRP9 | 12.7 | 147.5 | Short |
| 17 | SRSF1 | 6.4 | 188.3 | Short |
| 1 | SRSF10 | 11.4 | 54.4 | Short |
| 17 | SRSF2 | 3.3 | 178.7 | Short |
| 6 | SRSF3 | 10.2 | 146.8 | Short |
| 20 | SRSF6 | 5.7 | 204.5 | Short |
| 12 | SRSF9 | 8.1 | 167.1 | Short |
| 7 | SSBP1 | 12.2 | 71.3 | Short |
| 9 | SSNA1 | 1.8 | 125.4 | Short |
| 1 | SSR2 | 11.9 | 344.4 | Short |
| 11 | SSRP1 | 9.9 | 151.5 | Short |
| 16 | STUB1 | 2.7 | 136.1 | Short |
| 16 | STX4 | 7.1 | 54.5 | Short |
| 9 | SURF4 | 14.6 | 125.6 | Short |
| 1 | SYF2 | 10.3 | 73.8 | Short |
| 4 | TADA2B | 14.5 | 30.9 | Short |
| 3 | TADA3 | 13.1 | 77.9 | Short |
| 11 | TAF10 | 1.4 | 99.6 | Short |
| 16 | TAOK2 | 14.5 | 48.2 | Short |
| 1 | TARDBP | 12.9 | 114.9 | Short |
| 19 | TBCB | 11.0 | 134.5 | Short |
| 6 | TCP1 | 11.2 | 130.8 | Short |
| 16 | TERF2IP | 9.7 | 680.2 | Short |
| 2 | TEX261 | 8.9 | 103.3 | Short |
| 16 | THAP11 | 1.9 | 44.1 | Short |
| 23 | TIMM17B | 4.7 | 39.1 | Short |
| 17 | TIMM22 | 5.0 | 17.6 | Short |
| 19 | TMED1 | 3.9 | 35.5 | Short |
| 7 | TMED4 | 3.1 | 105.0 | Short |
| 5 | TMED9 | 3.9 | 198.2 | Short |
| 17 | TMEM101 | 3.8 | 39.0 | Short |
| 19 | TMEM147 | 1.9 | 169.6 | Short |
| 9 | TMEM203 | 1.6 | 52.2 | Short |
| 16 | TMEM219 | 11.0 | 90.9 | Short |
| 20 | TMEM230 | 13.6 | 82.8 | Short |
| 14 | TMEM251 | 2.1 | 24.9 | Short |
| 17 | TMEM256 | 1.2 | 115.8 | Short |
| 11 | TMEM258 | 3.5 | 59.4 | Short |
| 19 | TMEM259 | 11.5 | 270.4 | Short |
| 3 | TMEM42 | 3.8 | 57.8 | Short |
| 7 | TMEM60 | 4.7 | 27.0 | Short |
| 22 | TOMM22 | 2.8 | 47.5 | Short |
| 6 | TOMM70 | 3.1 | 300.1 | Short |
| 9 | TOR1A | 11.2 | 25.9 | Short |
| 9 | TOR1B | 8.1 | 21.0 | Short |
| 12 | TPI1 | 3.5 | 466.6 | Short |
| 1 | TRAPPC3 | 13.0 | 40.6 | Short |
| 19 | TRIM28 | 6.3 | 166.0 | Short |
| 11 | TRMT112 | 0.9 | 161.8 | Short |
| 2 | TSN | 12.3 | 43.5 | Short |
| 6 | TSPYL1 | 5.3 | 244.0 | Short |
| 17 | TSR1 | 14.7 | 21.0 | Short |
| 16 | TSR3 | 2.6 | 87.9 | Short |
| 8 | TSTA3 | 5.0 | 89.1 | Short |
| 17 | TUBG1 | 5.9 | 51.2 | Short |
| 3 | TUSC2 | 3.3 | 42.3 | Short |
| 22 | TXN2 | 14.6 | 98.4 | Short |
| 21 | U2AF1 | 14.6 | 1.0 | Short |
| 1 | UBE2Q1 | 10.1 | 42.8 | Short |
| 23 | UBL4A | 2.9 | 47.2 | Short |
| 19 | UBL5 | 2.2 | 399.4 | Short |
| 11 | UBXN1 | 2.6 | 288.4 | Short |
| 1 | UFC1 | 5.1 | 106.6 | Short |
| 13 | UFM1 | 13.2 | 49.7 | Short |
| 1 | UROD | 3.5 | 49.6 | Short |
| 4 | UTP3 | 2.1 | 26.2 | Short |
| 17 | VPS25 | 6.2 | 47.7 | Short |
| 12 | VPS29 | 10.6 | 67.5 | Short |
| 6 | WDR46 | 10.4 | 46.4 | Short |
| 5 | WDR55 | 6.2 | 18.2 | Short |
| 19 | WDR83OS | 1.6 | 94.8 | Short |
| 11 | YIF1A | 4.6 | 95.4 | Short |
| 6 | YIPF3 | 5.2 | 121.5 | Short |
| 7 | YKT6 | 13.3 | 61.2 | Short |
| 4 | ZNF330 | 13.8 | 52.9 | Short |
| 1 | ZNF593 | 1.0 | 0.1 | Short |
| 8 | ZNF706 | 9.0 | 54.8 | Short |
| 19 | ZNF865 | 5.0 | 27.6 | Short |
| 11 | ZFPL1 | 4.2 | 47.9 | Short |
| 5 | ZMAT2 | 6.2 | 136.5 | Short |
| 9 | ZMYND19 | 8.4 | 29.7 | Short |
